# Reproducible Ethanol-Induced Precipitation and Systematic Characterization of an α-D-Glucopyranose-Rich Solid from Khalas Date Fruit (*Phoenix dactylifera* L.)

**DOI:** 10.3390/foods15203632

**Published:** 2026-10-13

**Authors:** Reem A. Al Alawi, Younis Baqi

**Affiliations:** Department of Chemistry, College of Science, Sultan Qaboos University, Al Khod, P.O. Box 36, Muscat 123, Oman; r.alalawi2@squ.edu.om

**Keywords:** date palm, D-glucopyranose, mutarotation, natural product, *Phoenix dactylifera* L.

## Abstract

In an attempt to prepare ethanolic extracts of date fruit (*Phoenix dactylifera* L.) from the *Khalas* variety, a white solid precipitate was reproducibly obtained from three independent batches. A systematic analytical approach established that the isolated solid consisted predominantly of α-D-glucopyranose together with a small amount of D-fructose. TLC indicated monosaccharide behavior, LC–MS confirmed the presence of hexose species, and NMR spectroscopy established α-D-glucopyranose as the predominant component while providing spectroscopic support for a minor D-fructose component. Following identification of these two hexoses, polarimetric analysis based on their opposite specific rotations yielded an estimated composition of 93.8% D-glucose and 6.2% D-fructose under the applied binary glucose–fructose model. The α-anomeric proton (H-1) appeared as a doublet at δ 5.20 ppm (*J* = 3.5 Hz), consistent with an axial–equatorial relationship between H-1 and H-2. Mutarotation in aqueous solution shifted the equilibrium toward the thermodynamically more stable β-anomer, as evidenced by the decrease in the relative integral of the α-anomeric doublet at δ 5.20 ppm and the corresponding increase in the β-anomeric doublet at δ 4.62 ppm (*J* = 8.0 Hz), consistent with a diaxial relationship between H-1 and H-2. The present findings demonstrate the reproducible direct ethanol-induced isolation of an α-D-glucopyranose-rich solid from Khalas date fruit without prior aqueous extraction, together with anomer-specific characterization of the isolated material and its subsequent mutarotation in aqueous solution.

## 1. Introduction

**Figure 1:**
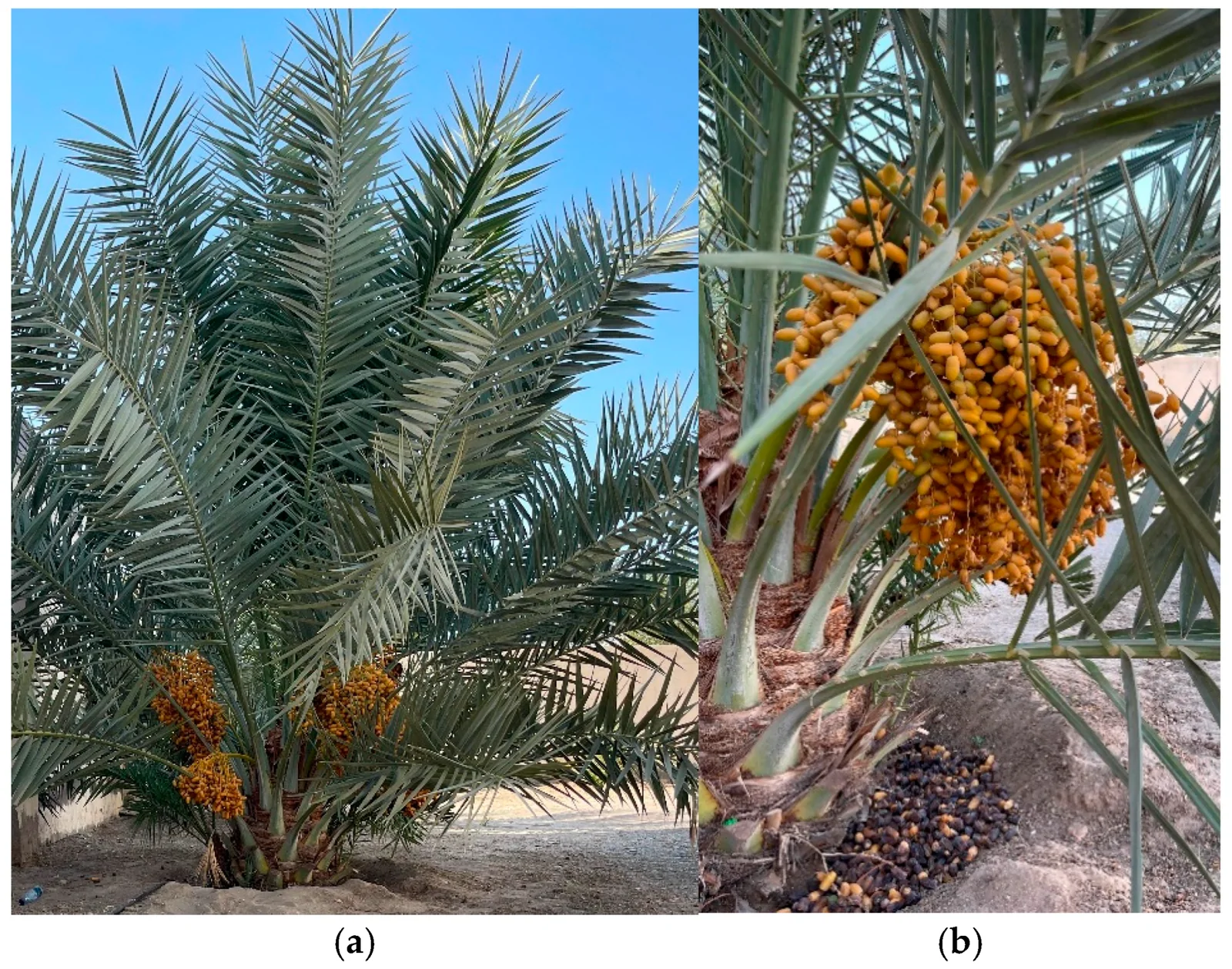
*Phoenix dactylifera* L.; (**a**) Date palm tree, (**b**) Date fruit.

The date palm tree (*Phoenix dactylifera* L.) is a flowering plant species in the Arecaceae family, which is well-known for the production of sweet fruit called the date fruit (Figure 1). The species is widely cultivated in arid and semi-arid regions, particularly the Middle East and North Africa (MENA), the Indian subcontinent, the Horn of Africa, China, South Asia, Australia, South West Europe, and California in the United States [1,2,3]. Dates are often described as the “bread of the desert” because of their importance as a staple food in the Middle East, North Africa, and other regions, where they serve as a natural source of energy and provide a wide range of essential nutrients [4].

Date fruit contains bioactive secondary metabolites, including flavonoids, phenolic acids, and carotenoids, which contribute to its reported biological activities [4,5,6,7,8,9,10]. Many plant secondary metabolites occur as glycosides; therefore, characterization of the monosaccharide (glycone) components is relevant to understanding glycosidic constituents in date fruit [11]. Although the sugar composition of date fruit has been widely investigated, most previous studies have focused on the identification and quantification of sugars within crude extracts rather than on their direct isolation as solids. Previous NMR studies of date sugars either isolated glucose from aqueous date-fruit extracts or reconstituted dried date extracts in D_2_O-containing aqueous buffer before spectral analysis, conditions that permit α/β interconversion by mutarotation [12,13,14]. In contrast, the present procedure uses absolute ethanol for extraction and precipitation, with no aqueous step before collection of the isolated solid, allowing the anomeric character of the precipitated glucose-rich fraction to be examined before subsequent aqueous mutarotation. The solubility behavior of D-glucose and D-fructose in ethanol/water mixtures has been extensively investigated [15,16,17] and provides relevant background for interpreting the present ethanol-induced precipitation. However, these studies primarily address sugar solubility and phase behavior in defined solvent systems, whereas the present study examines the reproducible direct precipitation of a glucose-rich fraction from the complex Khalas date-fruit matrix using absolute ethanol.

**Figure 2:**
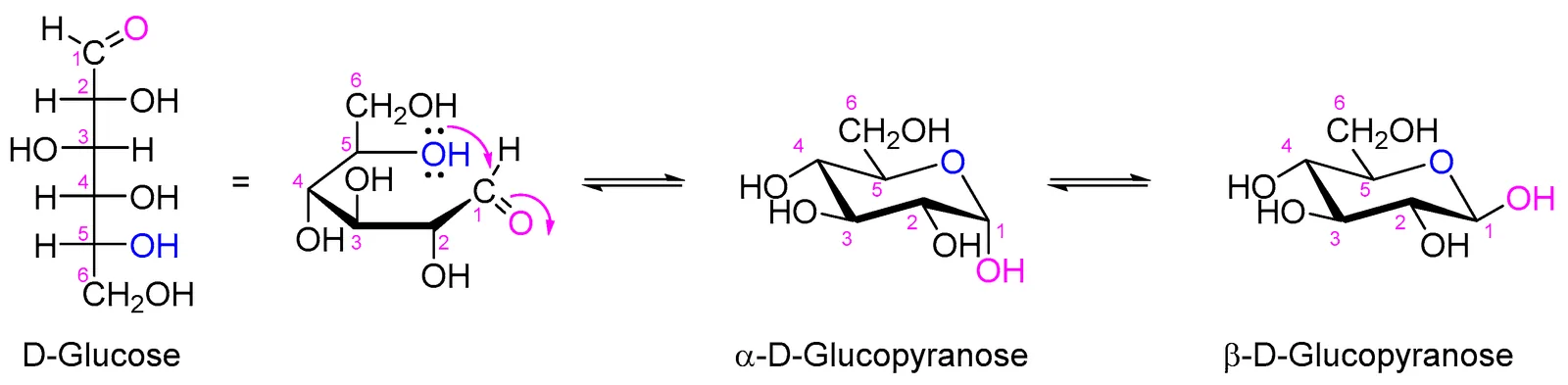
Structural representation of D-glucose and its pyranose α- and *β*-anomer.

The monosaccharide glucose occurs naturally in the D-form and exists in the cyclic hemiacetal form, generating two naturally occurring anomers designated *α*- and *β*-anomers (Figure 2). D-Glucose is a simple sugar that serves as the primary energy substrate for cellular respiration [18]. It is one of the most abundant monosaccharides in nature, serving as a primary energy source, a source of other monosaccharides, and a structural foundation for complex carbohydrates such as starch, glycogen, and cellulose, and playing a vital role in biological systems [19]. Knowing the monosaccharides (the glycone) existing in a plant will be a step forward in identifying the glycosides that exist in that particular plant, especially when multiple sugar units are attached to the natural product (the aglycone) [11]. In aqueous solutions, D-glucopyranose primarily exists in its stable cyclic pyranose form, which is more energetically favorable than the open-chain configuration [20]. This stability arises from favorable intramolecular interactions and steric factors that are less favorable in the open-chain form [20].

The chemical structure of D-glucopyranose consists of a six-membered pyranose ring formed through an intramolecular reaction between the hydroxyl group on carbon-5 (C5) and the aldehyde group on carbon-1 (C1). This reaction results in the formation of a hemiacetal and introduces an anomeric carbon at C1. The orientation of the hydroxyl group on this anomeric carbon determines whether the sugar adopts the alpha (α) or beta (β) anomeric form [21]. In the α-anomer, the hydroxyl group is axial on C1, whereas in the β-anomer, the hydroxyl group is equatorial, a configuration that is more stable due to reduced steric hindrance [22]. The dynamic nature of this behavior is critical to numerous biological and chemical processes involving glucose [23].

Although the solubility of individual sugars in ethanol/water systems is well established, the present work addresses their behavior within the complex date-fruit matrix by examining the reproducible ethanol-induced precipitation and direct isolation of an α-D-glucopyranose-rich fraction from the edible flesh of the Khalas variety of *Phoenix dactylifera* L. The absence of an aqueous extraction step before isolation enabled anomer-specific NMR characterization immediately after dissolution of the isolated fraction in DMSO-*d*_6_, revealing a pronounced predominance of the α-anomer; subsequent measurements in D_2_O demonstrated its mutarotation toward the β-anomer. The isolated fraction was systematically characterized using complementary chromatographic, spectroscopic, mass-spectrometric, and polarimetric techniques, with particular attention to its anomeric behavior and the reproducibility of the isolation procedure across independent batches.

## 2. Materials and Methods

### 2.1. General

All chemicals and solvents were purchased from Sigma-Aldrich Chemie GmbH (Taufkirchen, Germany) and were used as received. Analytical thin-layer chromatography (TLC) was performed on pre-coated Whatman^®^ LK6F silica gel 60 Å plates (Whatman, Maidstone, UK; layer thickness: 250 µm). Compounds were visualized by spraying the TLC plate with *p*-anisaldehyde stain solution (PAA) and then heating it at 105–110 °C. ^1^H and ^13^C NMR spectra were obtained on a Bruker Avance spectrometer (Bruker BioSpin GmbH, Rheinstetten, Germany) operating at 500 MHz for ^1^H NMR and 126 MHz for ^13^C NMR. DMSO-*d*_6_ or D_2_O was used as a solvent. Chemical shifts (δ) are reported in parts per million (ppm) relative to the residual solvent signals, that is, DMSO-*d*_6_ (δ*_H_* = 2.50 ppm, δ*_c_* = 39.52 ppm) and D_2_O (δ*_H_* = 4.79 ppm). Coupling constants (*J*) are reported in Hertz (Hz), and spin multiplicities are given as s (singlet), d (doublet), t (triplet), and m (multiplet). LC–MS analyses were performed using an Applied Biosystems API 2000 LC–MS system (Applied Biosystems/MDS SCIEX, Concord, ON, Canada) coupled to an Agilent 1100 HPLC system (Agilent Technologies, Waldbronn, Germany). Optical rotations were measured with a Polartronic M100 digital polarimeter (Schmidt + Haensch, Berlin, Germany) at 20 °C, using the sodium D line (λ = 589 nm).

### 2.2. Plant Materials

*Khalas* date fruits at the Tamer stage (dried) were purchased from the local market in Muscat, Oman, in September 2016, September 2017, and September 2021. Moisture content and °Brix were not determined. The initial quantitative glucose and fructose contents of the individual batches were also not determined. The variety was identified based on farmers’ and local people’s experience, as it is one of the most widely distributed date fruit varieties in Oman. Only ripe, undamaged, and unspoiled fruits were selected to eliminate contamination and ensure consistent outcomes in the study.

### 2.3. Extraction Procedure

**Figure 3:**
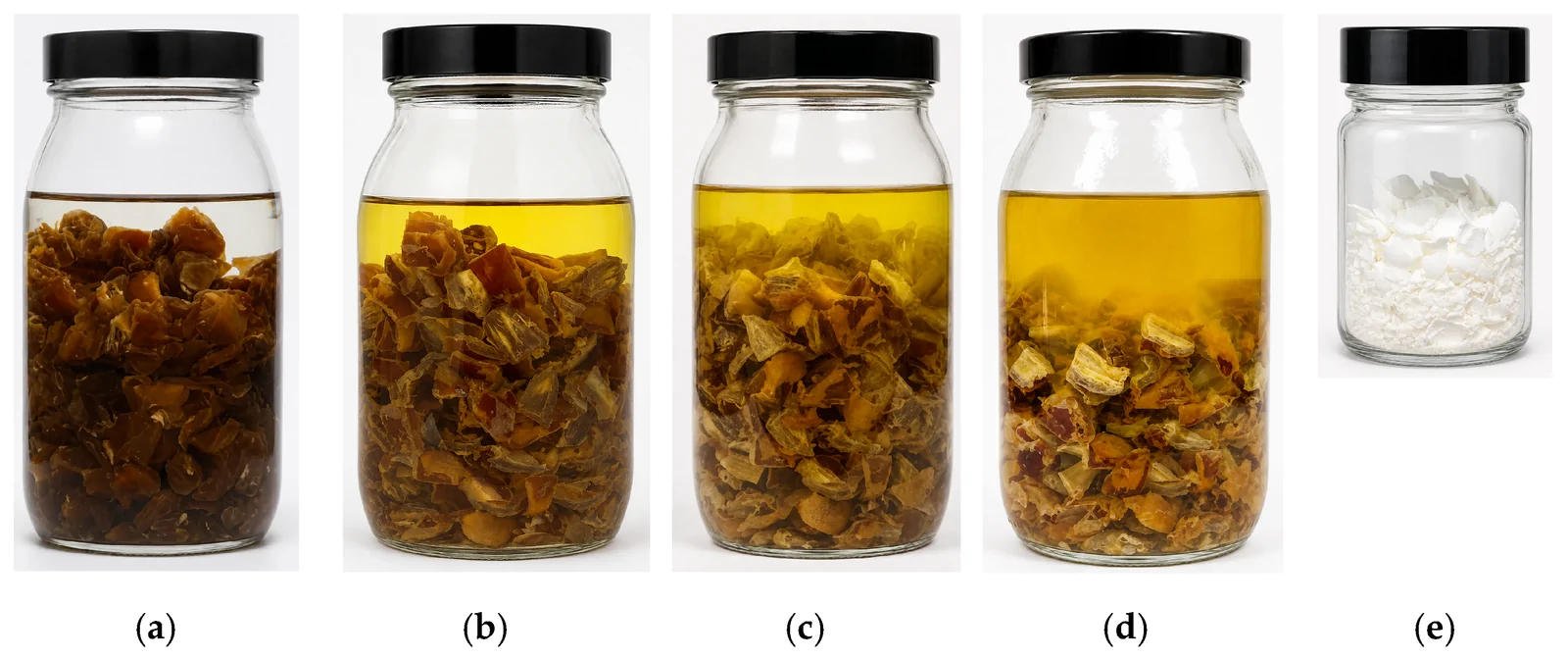
The pictures illustrate the changes in the color of the ethanolic crude extract during soaking and of the isolated white solid; (**a**) Upon soaking in ethanol, (**b**) After one day of soaking, (**c**) After two days of soaking, (**d**) After three days of soaking, (**e**) The isolated *α*-D-glucopyranose-rich white solid.

For each independent batch, the pits were removed from Khalas date fruit, and the edible flesh (2.0 kg) was cut into approximately 12 pieces per fruit. The date-fruit flesh was divided among ten 500 mL screw-cap glass bottles, with 200 g placed in each bottle. Absolute ethanol (300 mL) was added to each bottle, giving a total ethanol volume of 3.0 L per batch. The bottles were tightly closed and stored in the dark at room temperature for 3 days. During this period, the bottles were shaken manually approximately five times per day to facilitate the transfer of soluble components into the ethanol. A white layer gradually formed on the fruit pieces, while the solvent developed a yellow color (Figure 3a–d). After 3 days, the contents were filtered under reduced pressure using a Büchner funnel. The combined ethanolic filtrate was left undisturbed in the dark at room temperature for 18 h, during which a white solid precipitated. The solid was collected by suction filtration and washed with ethyl acetate (3 × 50 mL). The washed fraction was dried in an oven at 50 °C before weighing. The isolated fraction (Figure 3e) was then stored at −20 °C until further analysis. The complete extraction and precipitation procedure was performed independently for each of the three date-fruit batches. Importantly, no aqueous extraction or dissolution step was introduced before collection of the precipitated solid, thereby minimizing the opportunity for aqueous mutarotation prior to isolation.

### 2.4. Chromatographic Analysis

#### 2.4.1. Preparation of *p*-Anisaldehyde Stain

*p*-Anisaldehyde stain solution was prepared by mixing *p*-anisaldehyde (3.7 mL), acetic acid (1.5 mL), and concentrated sulfuric acid (5 mL) in ethanol (135 mL). The solution was kept at room temperature in a dark place.

#### 2.4.2. Thin-Layer Chromatography (TLC) Profiling

The isolated fraction was chromatographed alongside authentic standards of natural monosaccharides, including D-glucose, D-fructose, D-mannose, D-galactose, and D-ribose. The TLC plate was air-dried and developed in a TLC chamber pre-saturated with a dichloromethane/methanol/water (65:27:8, *v*/*v*/*v*) mobile phase. After development of the TLC, the plate was air-dried, visualized under UV, sprayed with *p*-anisaldehyde stain solution, and heated on a hotplate at 105–110 °C. The monosaccharide reaction was monitored over time, R*_f_*, and color.

#### 2.4.3. LC–MS Analysis

LC–MS analyses were performed in both positive and negative electrospray ionization (ESI) modes. Samples were prepared at a concentration of 0.5 mg mL^−1^ in H_2_O/MeOH (1:1, *v*/*v*) containing 2 mM ammonium acetate, and 10 μL was injected onto a Phenomenex Luna C18 column (50 × 2.00 mm, 3 μm). Elution was performed using a water/methanol gradient containing 2 mM ammonium acetate, from 90:10 to 0:100 over 30 min at a flow rate of 250 μL min^−1^, with the gradient initiated after 10 min, as previously described [24].

### 2.5. Reproducibility and Statistical Analysis

The reproducibility of the precipitation and isolation procedure was evaluated using three independent *Khalas* date-fruit batches collected at different time points. The isolation yield was calculated as the mass of the dried isolated fraction divided by the initial date-fruit mass × 100. Results are presented as the mean ± sample standard deviation for three independent batches (n = 3). The corresponding NMR and LC–MS profiles were compared to assess the consistency of the isolated fractions. Detailed time-dependent polarimetric analysis was performed on a representative fraction from one batch after comparable isolation yields and consistent analytical profiles had been obtained for the three batches.

### 2.6. Optical Rotation

The optical rotation at the light wavelength of 589 nm was measured using a Polartronic M100 digital polarimeter (Schmidt + Haensch, Germany) equipped with a standard 1 dm cylindrical cell. The light source was a sodium lamp, and measurements were performed at the sodium D line (λ = 589 nm). All measurements were carried out at 20.0 ± 0.1 °C. The isolated fraction was dissolved in distilled water at a concentration of 1.0 g/100 mL, and the solution was transferred into the measurement cell immediately after preparation. Specific optical rotation values ${\left[\alpha \right]}_{D}^{20}$ are reported as degrees·mL·g^−1^·dm^−1^, and are calculated according to the equation:

$${\left[\alpha \right]}_{D}^{20}=\frac{\alpha }{c\times l}$$

where *α* is the observed rotation (degrees), *c* is the concentration (g/100 mL), and *l* is the path length (dm).

## 3. Results and Discussion

### 3.1. Reproducibility of the Isolation Procedure Across Independent Batches

The ethanol-maceration and precipitation procedure was independently applied to three Khalas date-fruit batches collected in September 2016, September 2017, and September 2021. Each batch was processed independently under the same experimental conditions using 2.0 kg of date-fruit flesh. A white fraction was consistently obtained from all three batches. The isolated masses and percentage yields were comparable despite the five-year collection period, indicating that the precipitation was reproducible and was not restricted to a single batch (Table 1).

### 3.2. Isolation of the α-D-Glucopyranose-Rich Fraction

During maceration of date fruit flesh in ethanol, the solvent gradually turned yellow, and a white layer formed on the surface of the fruit pieces, indicating that soluble compounds were successfully transferred into the ethanolic phase. After filtration, a white solid precipitated from the ethanolic filtrate upon standing at room temperature for 18 h. This behavior is consistent with the low solubility of glucose in ethanol [15].

The precipitated solids obtained from the three independent batches were washed with ethyl acetate, yielding 4.013, 3.897, and 4.145 g, corresponding to 0.2007, 0.1949, and 0.2073% (*w*/*w*), respectively. The mean isolated mass was 4.018 ± 0.124 g, corresponding to a mean yield of 0.2009 ± 0.0062% (*w*/*w*; n = 3). The relatively low isolated yield reflects the selective nature of the precipitation procedure; the objective of the present study was to characterize the isolated fraction and its anomeric composition rather than to maximize sugar recovery. The representative isolated fraction had a melting point of 141–142 °C, which was slightly lower than the reported value of 146 °C for α-D-glucopyranose. The melting point was considered only as a supporting physical characteristic and not as evidence of solid-state phase identity.

### 3.3. TLC Profiling

**Figure 4:**
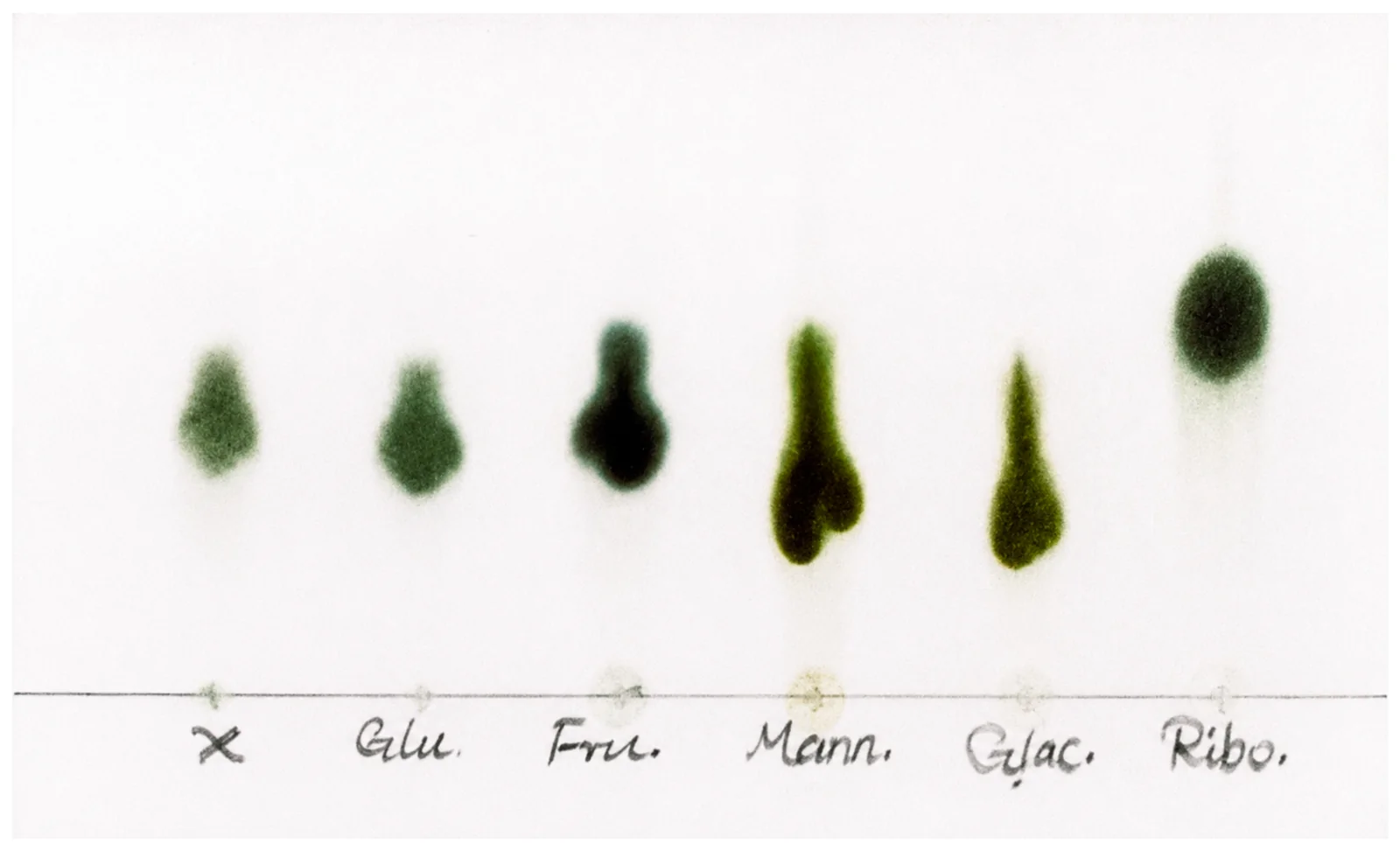
TLC picture of the isolated fraction and authentic monosaccharide standards. Lanes description; X; the isolated fraction, Glu; D-glucose, Fru; D-fructose, Mann; D-mannose, Glac; D-galactose, and Ribo; D-ribose.

Thin layer chromatography (TLC) is recognized as a rapid, simple, and cost-effective analytical technique for separating a wide range of monosaccharide samples based on their polarity and interactions with the silica stationary phase. For a preliminary investigation, TLC was used to compare the isolated fraction with five authentic monosaccharides: D-glucose, D-fructose, D-mannose, D-galactose, and D-ribose. As shown in Figure 4, the isolated fraction (first spot (X)) exhibited a single spot under the applied TLC conditions. Its Rf value and color most closely corresponded to those of D-glucose; however, the close Rf values of D-glucose and D-fructose do not allow reliable differentiation between them by this TLC system. Notably, D-ribose showed higher mobility, whereas D-mannose and D-galactose migrated differently. Thus, TLC provided preliminary qualitative support for D-glucose as the major component and was not used to determine purity or composition. LC–MS was subsequently used to confirm the presence of hexose species, whereas NMR spectroscopy was used to identify D-glucose as the predominant component and to provide support for the minor D-fructose component; their relative proportions were estimated by polarimetric analysis. The TLC results are summarized in Table 2.

### 3.4. Liquid Chromatography-Mass Spectrometry (LC–MS)

LC–MS was used to confirm the presence of hexose species in the isolated solid. Because D-glucose and D-fructose are constitutional isomers with the same molecular formula and molecular mass, LC–MS was used to confirm their molecular class rather than to distinguish between the two monosaccharides. Extracted-ion chromatograms (EICs) at *m*/*z* 179, 198, and 359 each showed a single sharp peak at RT ≈ 0.30 min, indicating minimal retention of the highly polar hexose species under the applied reversed-phase conditions. Integration of the corresponding peak accounted for 100% of the detected area within each extracted-ion trace. No additional peaks associated with the monitored hexose-related ions were detected under the applied conditions; however, this does not provide evidence of glucose/fructose separation, sample purity, or overall sample homogeneity.

**Figure 5:**
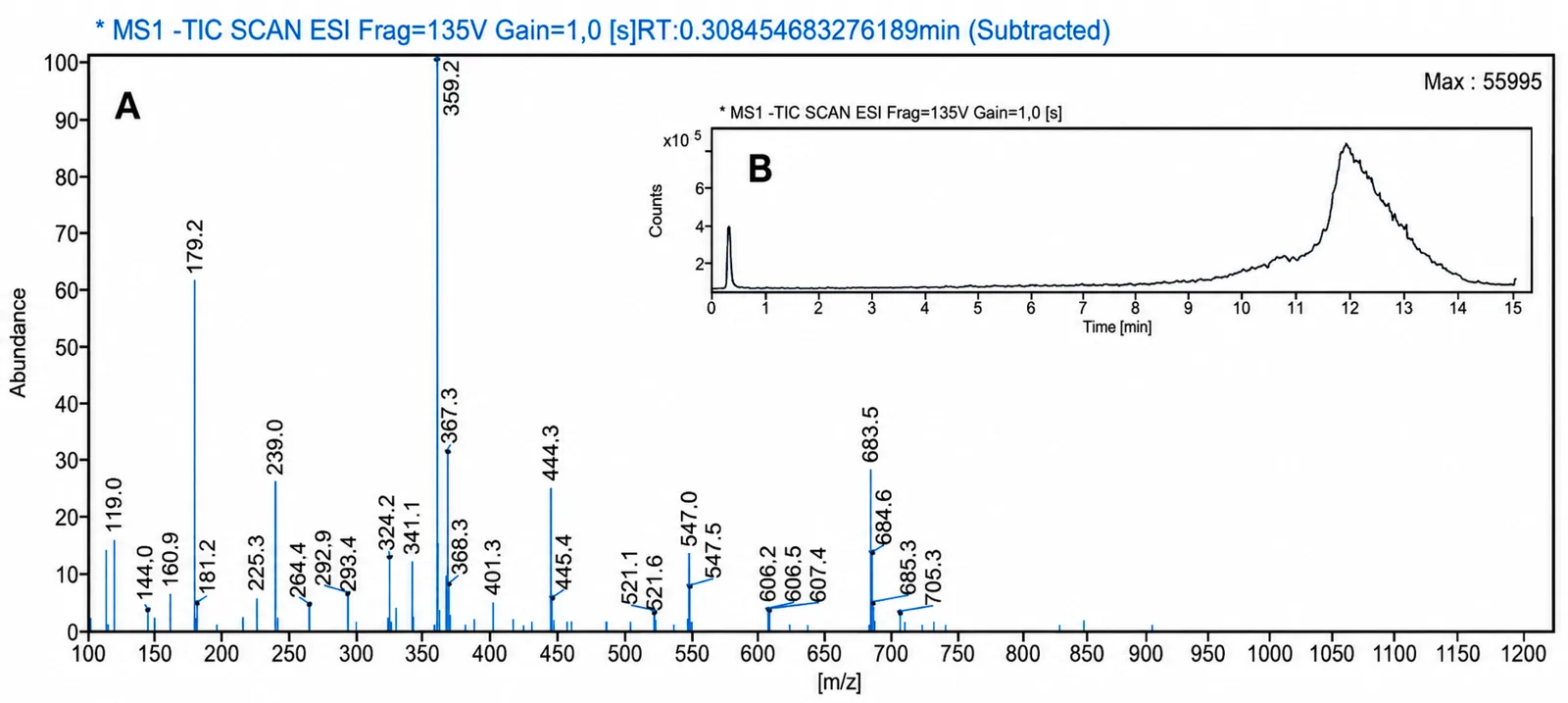
Negative mode LC–MS analysis; (**A**) ESI mass spectrum. (**B**) Total ion chromatogram.

**Figure 6:**
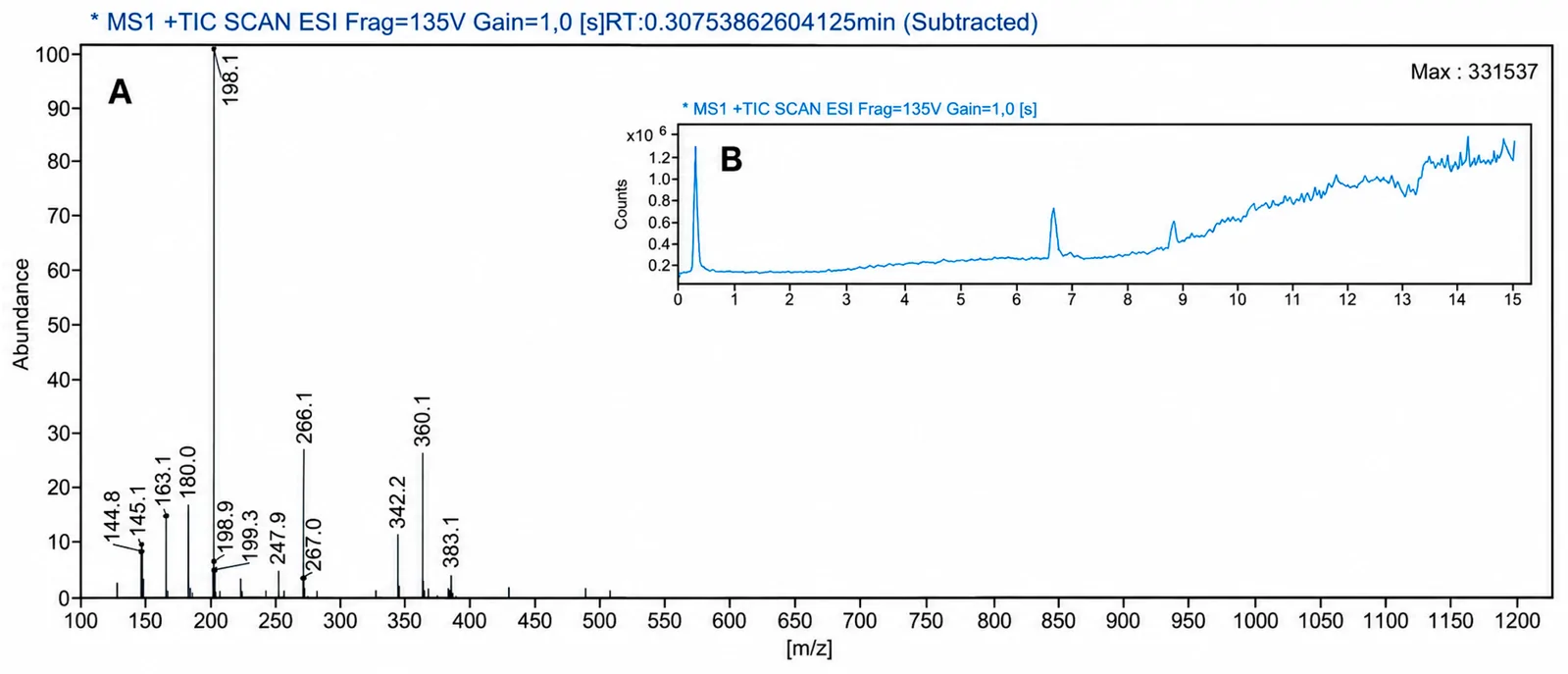
Positive mode LC–MS analysis; (**A**) ESI mass spectrum. (**B**) Total ion chromatogram.

The observation of the deprotonated monomer [M–H]^−^ at *m*/*z* 179.2 as a prominent signal (60% relative abundance) in negative mode (Figure 5) is consistent with the expected mass of deprotonated glucose and fructose (C_6_H_12_O_6_, MW = 180.0 Da). Because both compounds are constitutional isomers, the mass spectrum cannot distinguish between them. The most intense peak at *m*/*z* 359.2 is attributable to the [2M–H]^−^ dimer. In positive ESI mode, the base peak is at *m*/*z* 198.1, which corresponds to the ammonium adduct [M+NH_4_]^+^ of hexose (Figure 6). The signal at *m*/*z* 180.0 is consistent with loss of water from the ammonium adduct [M+NH_4_−H_2_O]^+^. As previously reported, glucose is not conveniently protonated to form [M+H]^+^ ions.

As a result, LC–MS analysis of glucose and other monosaccharides generally depends on generating more stable adduct ions rather than protonated glucose [25,26]. The ions at *m*/*z* 163 and 145 are consistent with dehydration fragments of protonated hexose, [M+H–H_2_O]^+^ and [M+H–2H_2_O]^+^, respectively. Collectively, the LC–MS results confirmed that the isolated solid contained hexose species. Identification of D-glucose as the predominant component and D-fructose as the minor component was therefore based on the complementary NMR results, while their relative proportions were estimated primarily from the polarimetric data.

### 3.5. NMR Characterization

The isolated fractions obtained from the three independent batches were compared by ^1^H, ^13^C, and DEPT-135 NMR spectroscopy in DMSO-*d*_6_ (Figures S4–S15 in the Supplementary Information). Detailed time-dependent NMR characterization of a representative fraction was subsequently performed in DMSO-*d*_6_ and D_2_O immediately after dissolution (t_0_) and after 24 h (t_24_). The NMR spectra established α-D-glucose as the predominant component and revealed weak additional signals consistent with a minor D-fructose component. The three batches showed consistent glucose-dominated NMR profiles, whereas the weak minor signals were interpreted qualitatively and were not used for quantitative determination. These results provided spectroscopic support for the presence of the two hexose components indicated by the complementary analytical findings. The use of DMSO-*d*_6_ was particularly beneficial because it retains the hydroxyl proton resonances, whereas D_2_O allowed efficient monitoring of the time-dependent α-to-β anomeric conversion during mutarotation. Because no aqueous extraction or dissolution step preceded isolation, the pronounced α-anomer predominance observed immediately after dissolution in DMSO-*d*_6_ is particularly informative of the anomeric character of the isolated fraction before subsequent exposure to aqueous conditions.

#### 3.5.1. Measurement of ^1^H NMR in Deuterated Dimethyl Sulfoxide (DMSO-d_6_)

**Figure 7:**
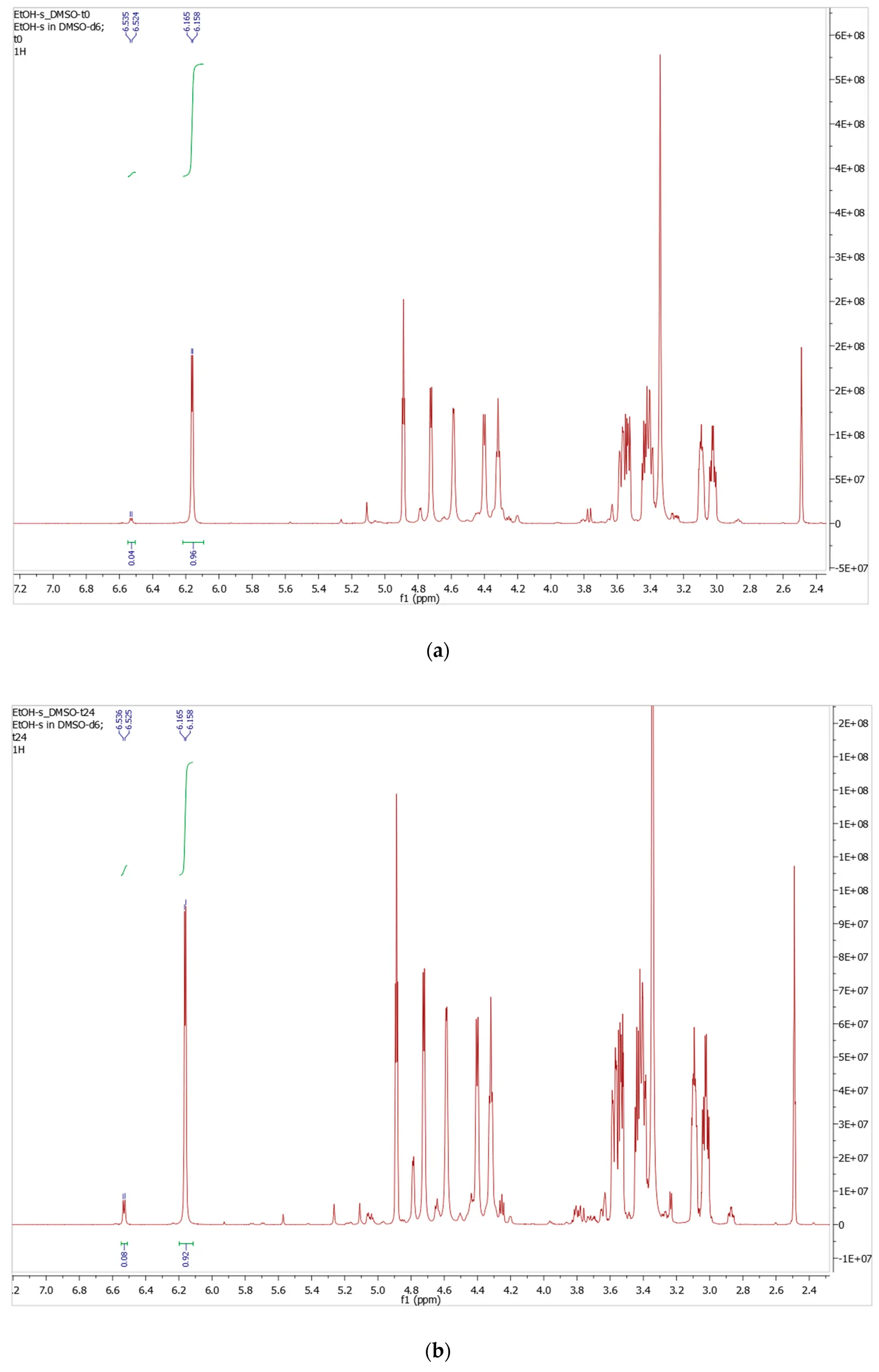
^1^H NMR spectra (DMSO-*d*_6_) of the isolated α-D-glucopyranose-rich fraction; (**a**) At time zero (t_0_), (**b**) After 24 h (t_24_).

The 1H NMR spectrum recorded immediately after dissolution (t_0_) in DMSO-*d*_6_ was dominated by a doublet at δ 6.16 ppm (*J* = 3.5 Hz) and a minor doublet at δ 6.53 ppm (*J* = 5.5 Hz), assigned to the anomeric hydroxyl protons (OH) of α- and β-D-glucopyranose, respectively (Figure 7). These assignments are consistent with previously published data [27,28]. Integration of the two anomeric hydroxyl proton signals yielded an α:β ratio of 96:4, indicating that the α-anomer was predominant immediately after dissolution. This pronounced α-anomer predominance therefore reflects the anomeric composition of the freshly dissolved isolated fraction prior to substantial mutarotation. The same NMR tube was kept for 24 h, and the NMR spectrum was measured again (t_24_); this time, the α:β ratio shifted to 92:8, reflecting partial progress toward mutarotation equilibrium between the two anomers of D-glucose. The relatively small change to the initial ratio suggests that mutarotation is slow due to the use of DMSO-*d*_6_. It has been reported that in anhydrous DMSO-*d*_6_, D-glucose mutarotation is expected to be strongly retarded because the aprotic solvent does not provide the efficient hydrogen-bonded proton-transfer network required for glucose ring opening [29,30,31].

^1^H NMR (DMSO-*d*_6_) δ ppm: 6.53 (d, *J* = 5.5 Hz, 1H, C1-OH, β-anomer), 6.16 (d, *J* = 3.5 Hz, 1H, C1-OH, α-anomer), 4.41–4.89 (m, 5H, overlapping anomeric H-1 and hydroxyl proton resonances of OH-2, OH-3, OH-4, and OH-6), 3.40–3.62 (m, 6H, H-2–H-6).

#### 3.5.2. Measurement of ^13^C NMR and DEPT-135 Spectra in Deuterated Dimethyl Sulfoxide (DMSO-d_6_)

**Figure 8:**
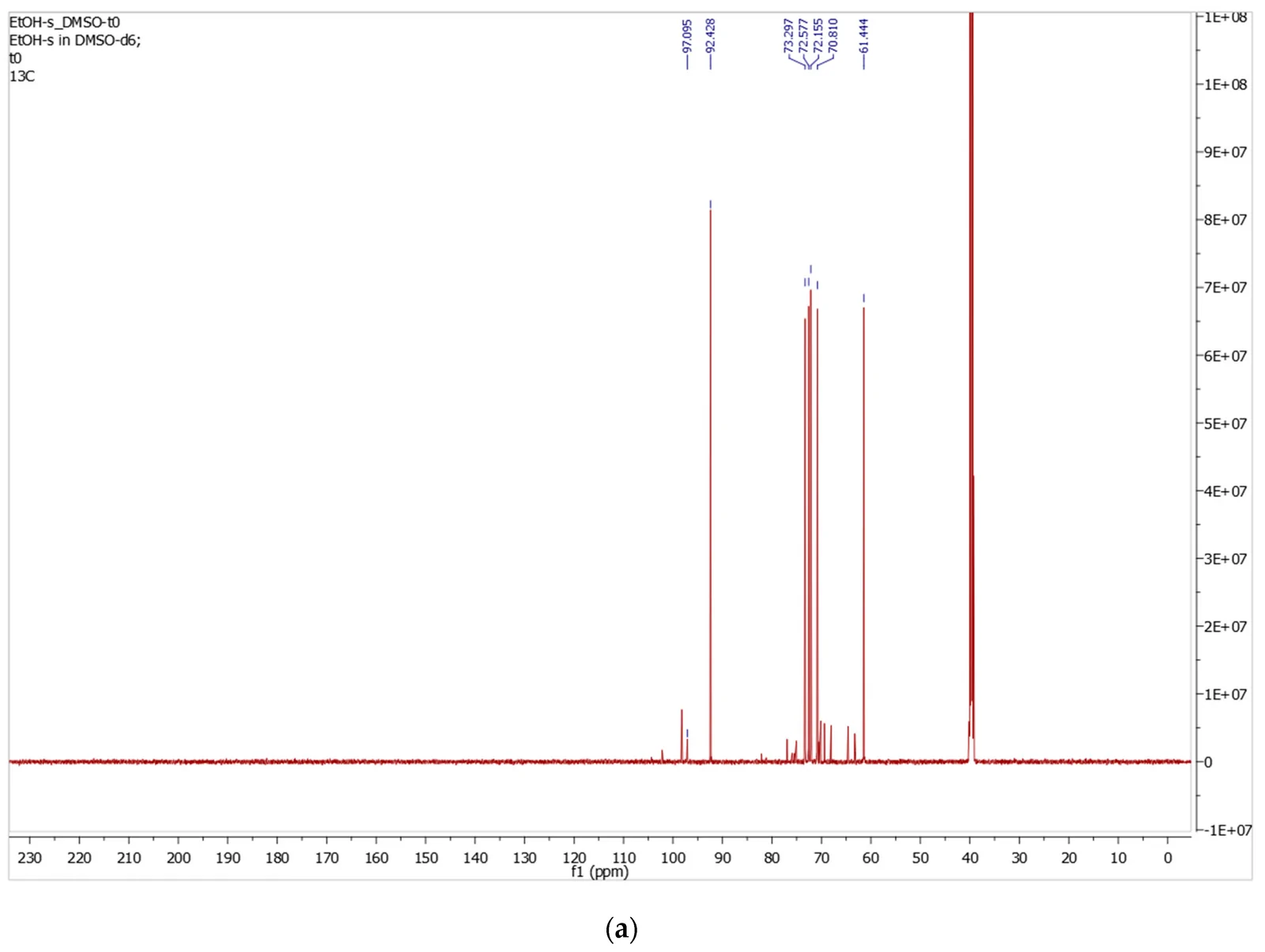

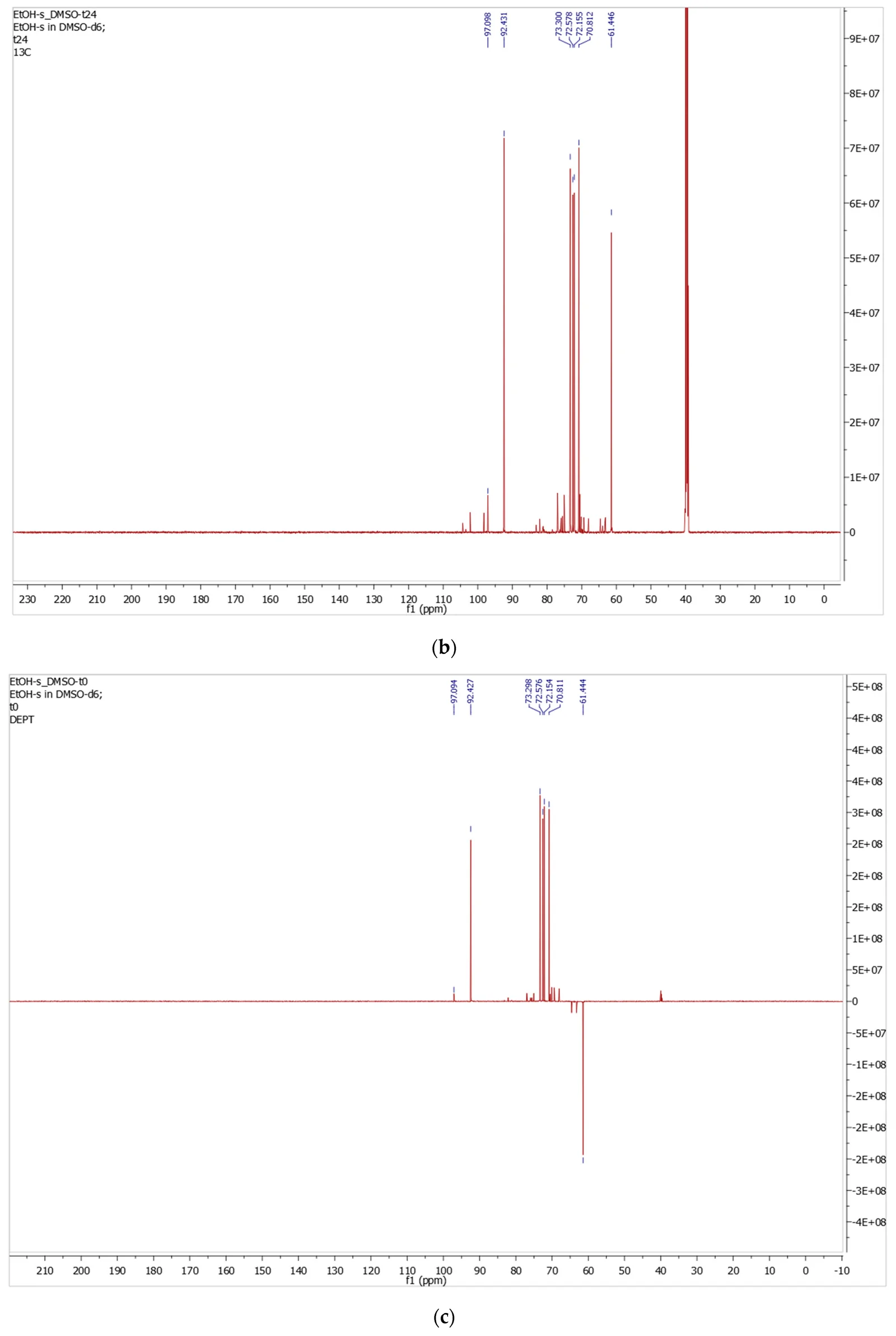

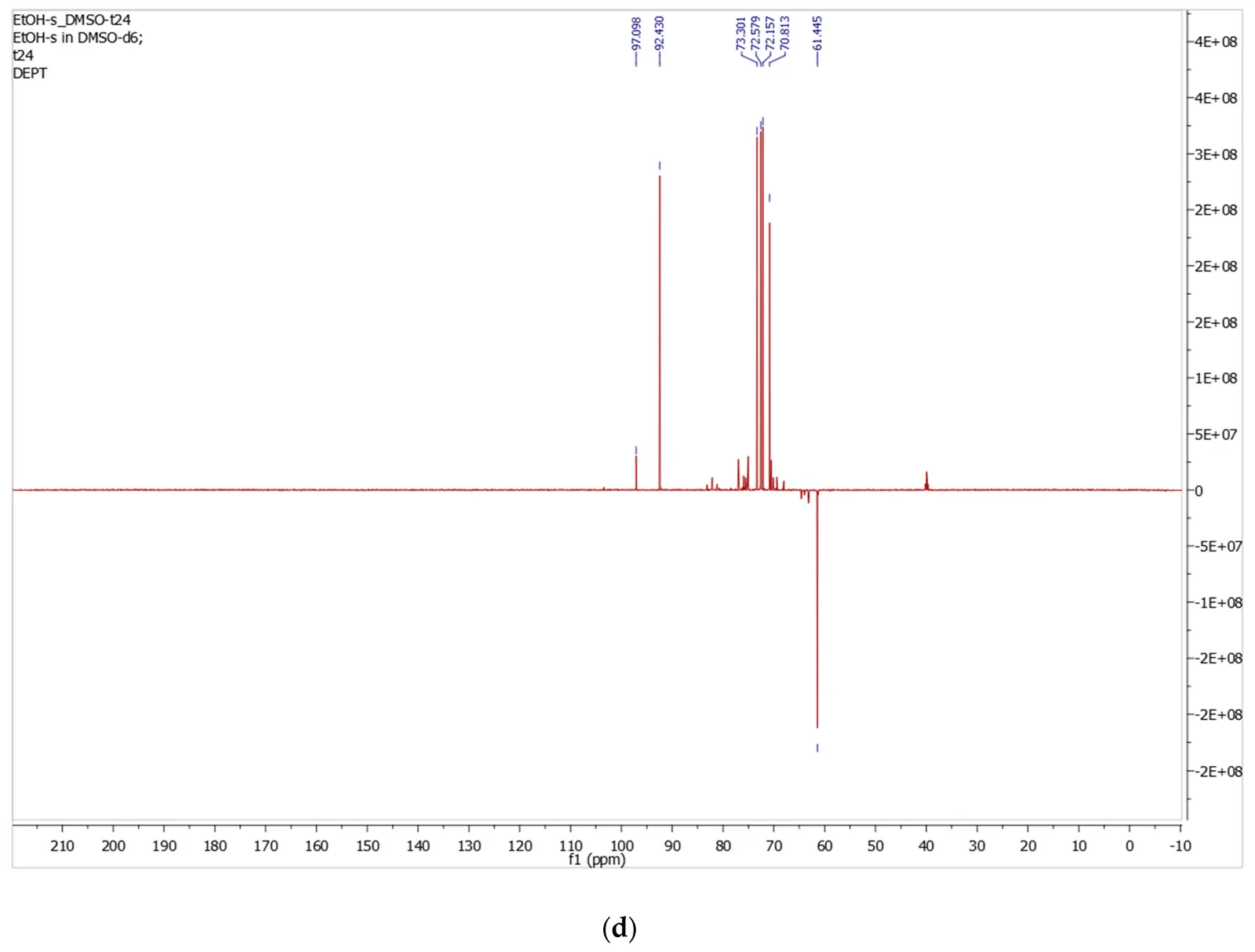
^13^C NMR and DEPT-135 spectra (DMSO-*d*_6_) of the isolated α-D-glucopyranose-rich fraction; (**a**) ^13^C NMR at time zero (t_0_), (**b**) ^13^C NMR after 24 h (t_24_), (**c**) DEPT-135 NMR at time zero (t_0_), (**d**) DEPT-135 NMR after 24 h (t_24_).

The ^13^C NMR spectrum in DMSO-*d*_6_ (Figure 8a) displays an anomeric carbon signal at δ 92.4 ppm, assigned to C-1 of α-D-glucopyranose, alongside a cluster of ring carbon resonances in the region δ 71–73 ppm (C-2, C-3, C-4, and C-5) and the primary hydroxymethyl carbon C-6 at δ 61.4 ppm. An additional, weaker signal is observed at δ 97.1 ppm, attributable to C-1 of the β-D-glucopyranose anomer, consistent with the minor β-anomer proportion established by ^1^H NMR integration.

After 24 h (t_24_), the ^13^C NMR spectrum showed a slight increase in the intensity of the β-anomeric carbon signal at δ 97.1 ppm, consistent with limited α-to-β mutarotation in DMSO-*d*_6_ (Figure 8b).

DEPT-135 NMR was subsequently used to support the carbon multiplicity assignments (Figure 8c,d).

The anomeric C-1 and ring carbons C-2 through C-5 appear in the positive (CH) signals, whereas C-6 gives a negative (CH_2_) signal at δ 61.4 ppm, as expected for the primary hydroxymethyl group. In addition, two weak negative CH_2_ signals were observed in the δ 63–65 ppm region, consistent with the two hydroxymethyl carbons of a minor D-fructose component. These signals were distinct from the predominant D-glucopyranose resonances, and their relatively low intensities supported the presence of D-fructose as a minor constituent of the isolated solid.

After 24 h (t24), the DEPT-135 spectrum showed a slight increase in the β-anomeric carbon signal at δ 97.1 ppm, consistent with limited α-to-β mutarotation in DMSO-*d*_6_ (Figure 8d).

^13^C NMR (DMSO-*d*_6_) δ (ppm): 97.1 (CH, C-1β), 92.4 (CH, C-1α), 73.3, 72.6, 72.2, 70.8 (4 CH, C-2, C-3, C-4, C-5), 61.4 (CH_2_, C-6).

#### 3.5.3. ^1^H NMR Spectra in Deuterated Water (D_2_O)

**Figure 9:**
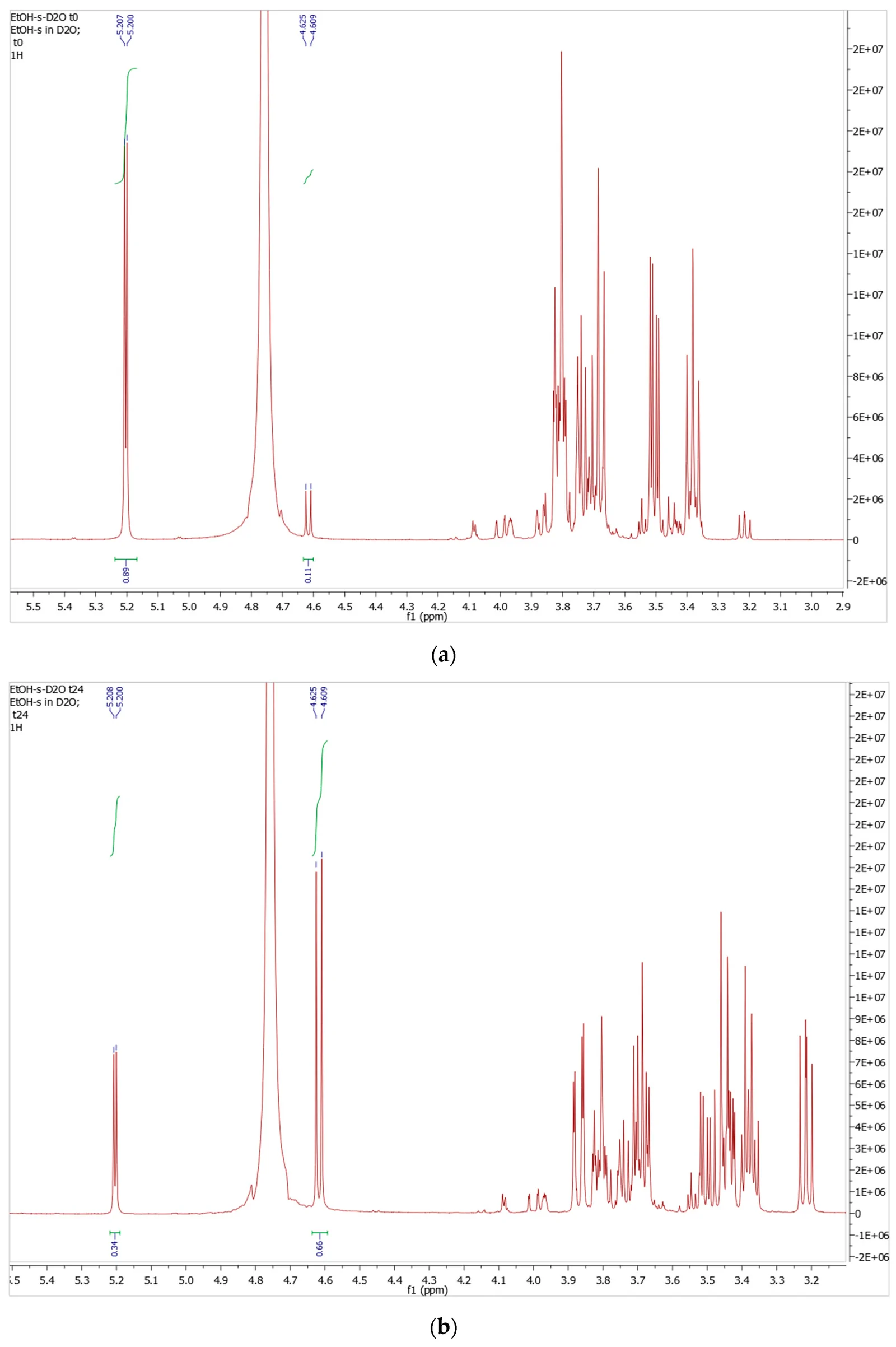
^1^H NMR spectra in water (D_2_O) of the isolated α-D-glucopyranose-rich fraction; (**a**) At time zero (t_0_), (**b**) After 24 h (t_24_).

The time-dependent ^1^H NMR analysis in deuterated water (D_2_O) clearly demonstrated the mutarotation of α-D-glucopyranose toward its thermodynamic equilibrium state. As shown in Figure 9a,b, two well-defined anomeric doublets at δ 5.207 ppm (H-1α, *J* = 3.5 Hz) and δ 4.625 ppm (H-1β, *J* = 8.0 Hz) at the initial time point (t_0_) provided an α:β ratio of 89:11, indicating a strong predominance of the α-anomer immediately after dissolution, consistent with the α-rich composition observed in DMSO-*d*_6_. After standing for 24 h at room temperature, the anomeric ratio shifted significantly to α:β = 34:66, reflecting substantial approach to thermodynamic equilibrium. This ratio agreed closely with the well-established equilibrium value of α:β ≈ 36:64 at 25 °C in aqueous solution, confirming that the system had approached equilibrium [32].

In aqueous solution, the β-anomer is more stable because all hydroxyl groups and the C6 hydroxy methyl group are in equatorial position, minimizing steric strain. Although the anomeric effect favors the α-anomer, this effect is not strong in aqueous solution since the β-form is more stabilized by interaction with the solvent [33]. The much greater extent of α-to-β conversion in D_2_O than in DMSO-*d*_6_ is consistent with more rapid mutarotation in the protic aqueous medium, which facilitates proton transfer during ring opening and closure [31]. The ring protons (H-2 to H-6) appear as overlapping signals (multiplet) between δ 3.20 and 3.90 ppm. The complexity of this region arises predominantly from the overlapping resonances of α- and β-D-glucopyranose, together with contributions from the minor D-fructose component, whose proton signals also occur within the carbohydrate region. Although the overlapping fructose signals were not used for direct quantitative integration, they were consistent with the presence of the minor D-fructose component supported by the complementary ^13^C NMR and DEPT-135 spectra.

At 0 h: ^1^H NMR (500 MHz, D_2_O) δ (ppm): 5.20 (d, *J* = 3.50 Hz, 1H, H-1α), 4.62 (d, *J* = 8.0 Hz, 1H, H-1β), 3.20–3.90 (m, 6H, H-2–H-6). At 24 h: ^1^H NMR (D_2_O) δ (ppm): 5.20 (d, *J* = 4.0 Hz, 1H, H-1α), 4.62 (d, *J* = 8.0 Hz, 1H, H-1β), 3.20–3.90 (m, 6H, H-2–H-6).

#### 3.5.4. ^13^C NMR and DEPT-135 NMR in Deuterated Water (D_2_O)

In the ^13^C NMR spectrum recorded in D_2_O, two anomeric carbon signals are observed at δ 98.6 ppm and δ 94.8 ppm at both t_0_ and t_24_, corresponding to C-1 of the β- and α-D-glucopyranose, respectively. Compared to DMSO-*d*_6_, both peaks are shifted downfield due to stronger hydrogen bonding in water. The ring carbons C-2 through C-5 appear in the region δ 72–79 ppm, and C-6 hydroxymethyl carbons resonate at δ 63.33 ppm.

**Figure 10:**
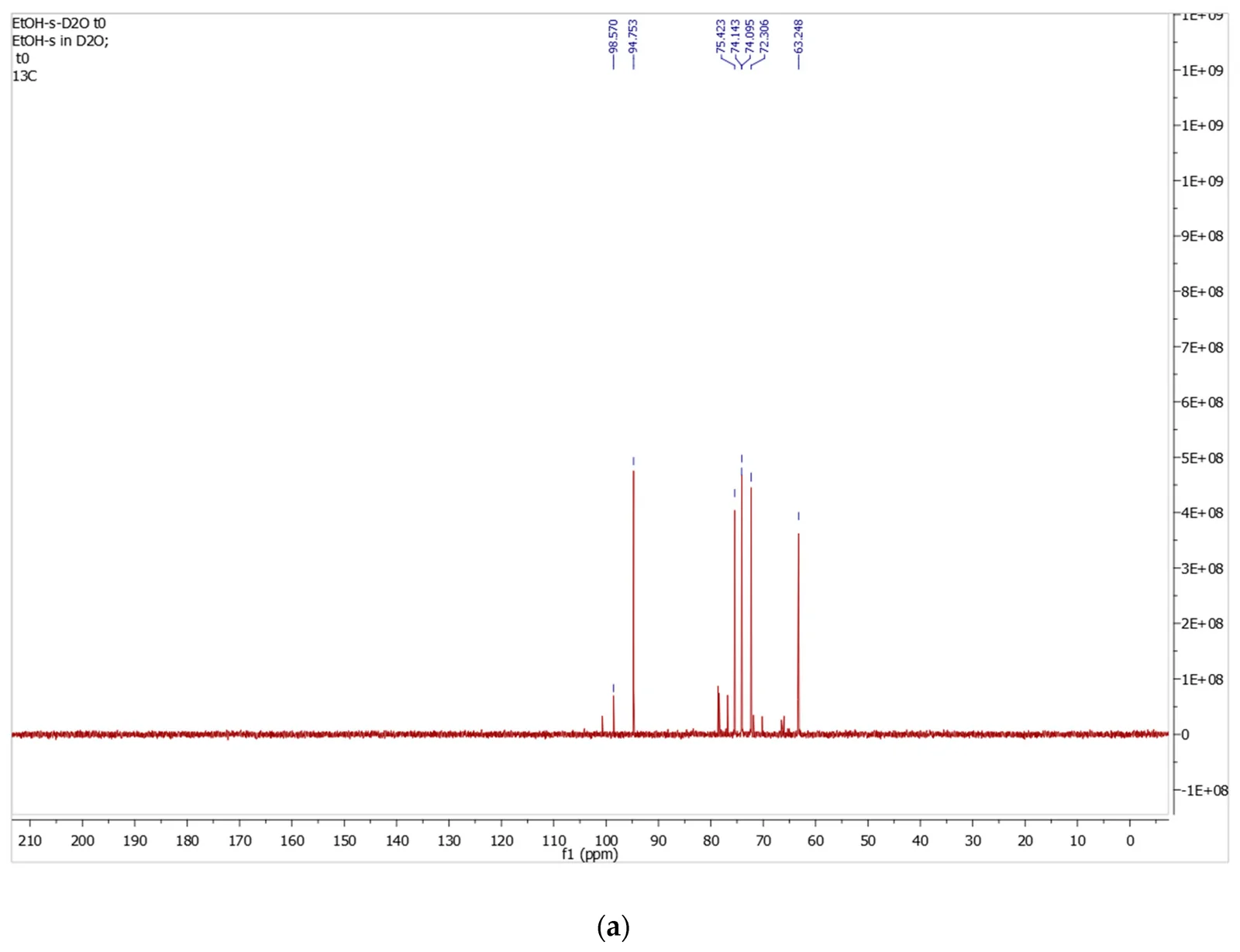

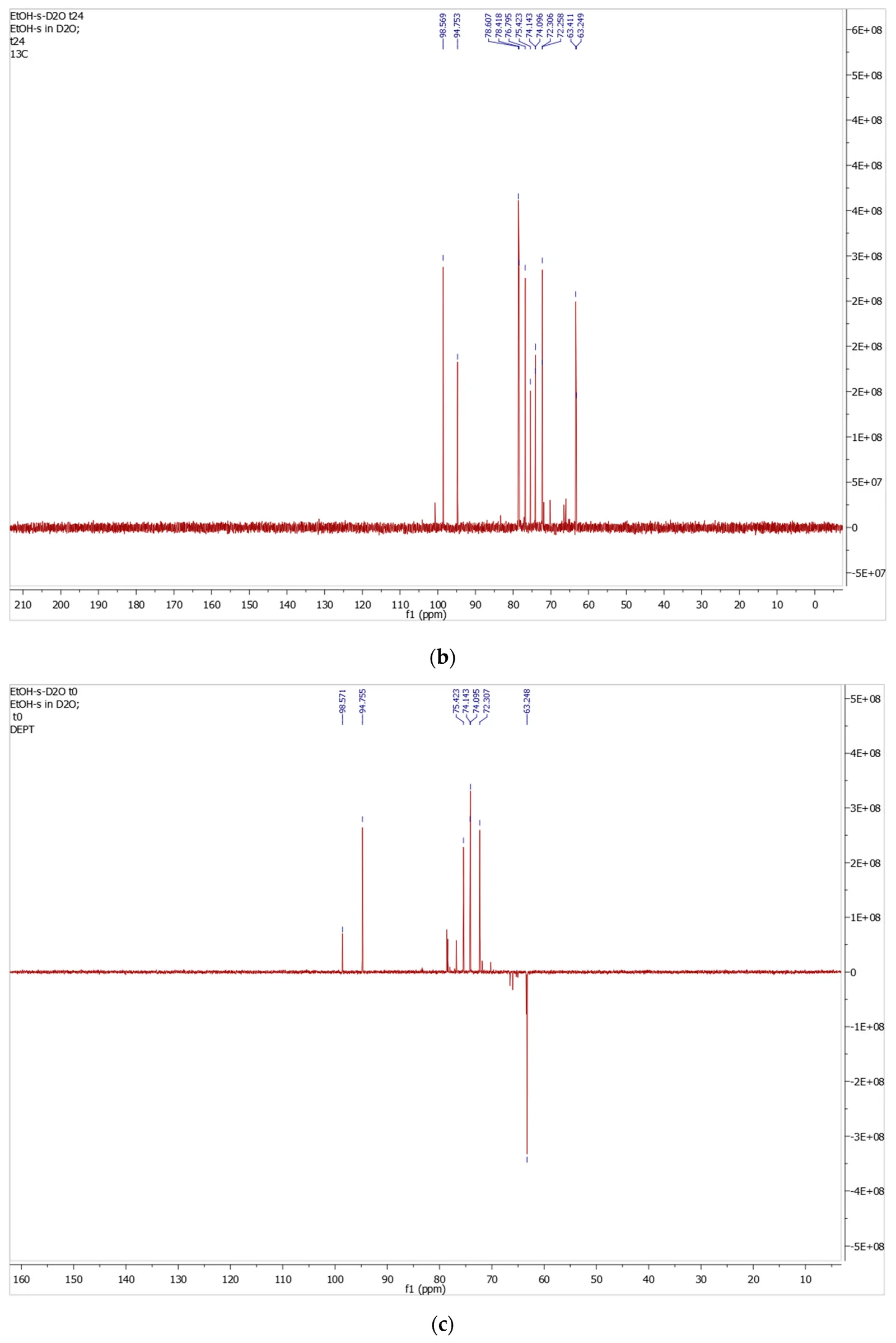

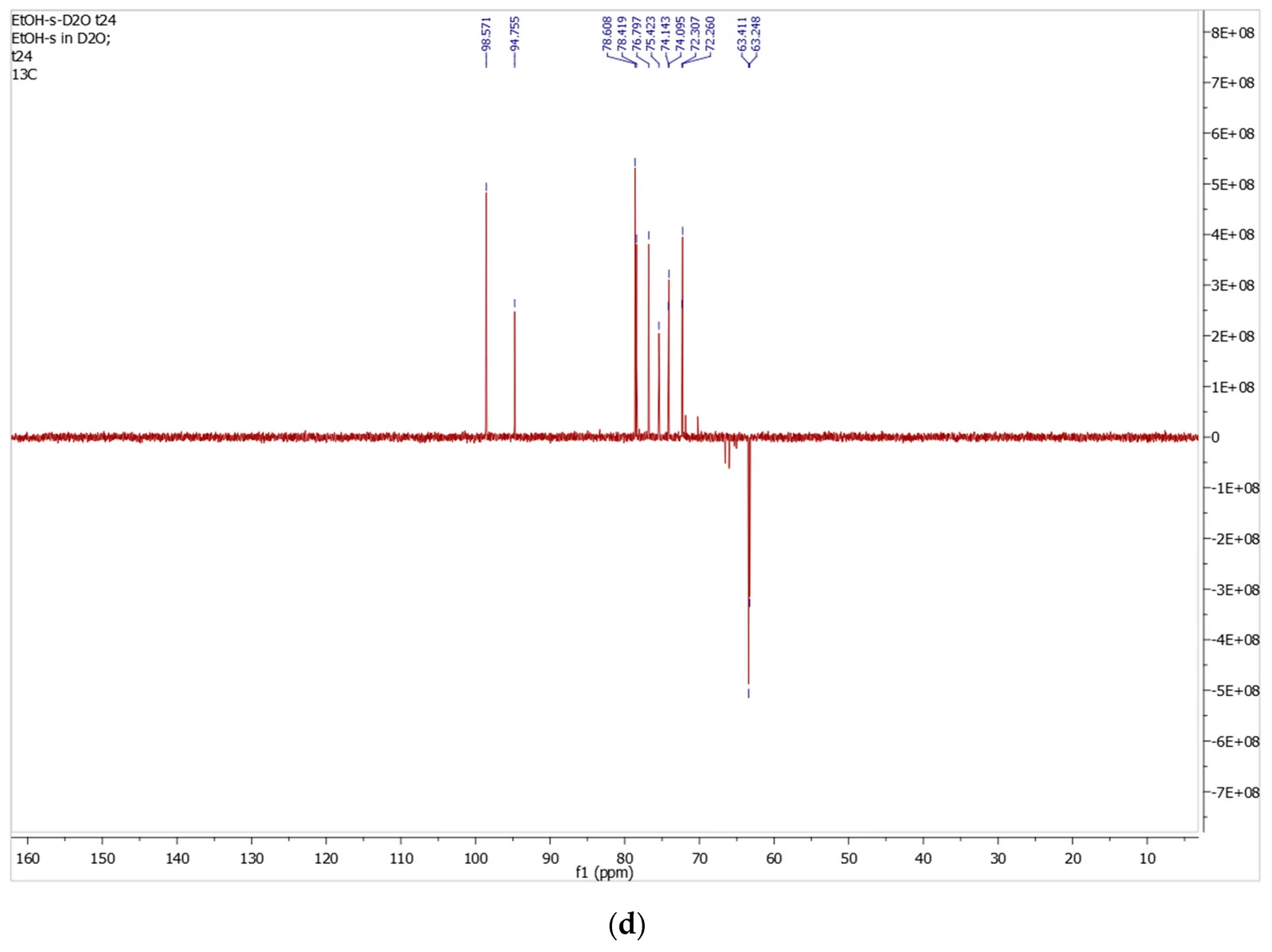
^13^C NMR and DEPT-135 spectra (D_2_O) of the isolated α-D-glucopyranose-rich fraction; (**a**) ^13^C NMR at time zero (t_0_), (**b**) ^13^C NMR after 24 h (t_24_), (**c**) DEPT-135 NMR at time zero (t_0_), (**d**) DEPT-135 NMR after 24 h (t_24_).

After 24 h (t_24_), the ^13^C NMR spectrum showed a marked increase in the β-anomeric carbon signal at δ 98.6 ppm (Figure 10b), consistent with the α:β ratio of 34:66 determined from the corresponding ^1^H NMR spectrum (Figure 10a).

The DEPT-135 NMR analysis supports the multiplicity assignments, with C-1 and C-2 to C-5 appearing as positive CH signals, while C-6 appears as a negative CH_2_ signal at δ 63.2 ppm.

After 24 h (t_24_), the DEPT-135 spectrum showed an increased β-anomeric carbon signal at δ 98.6 ppm, supporting the increased β-anomer proportion observed in the corresponding ^1^H and ^13^C NMR spectra (Figure 10d).

^13^C NMR (D_2_O) δ (ppm): 98.6 (CH, C-1β), 94.8 (CH, C-1α), 75.4, 74.14, 74.10, 72.3 (4CH, C-2, C-3, C-4, and C-5), 63.33 (CH_2_, C6).

### 3.6. Specific Rotation and Compositional Analysis

Following the identification of D-glucose as the predominant component and the detection of a minor D-fructose component by NMR spectroscopy, polarimetric analysis was used to estimate their relative proportions. The isolated solid was compared with a commercial α-D-glucose standard under identical experimental conditions of concentration, temperature, wavelength, and optical path length.

The commercial α-D-glucose standard showed an initial specific rotation of +106.5°, which decreased to an equilibrium value of +51.8° after 18 h. This value closely agreed with the reported equilibrium specific rotation of D-glucose in aqueous solution (+52.7°) [34], confirming proper instrument performance and the suitability of the experimental conditions. This comparison also provided a direct reference for interpreting the mutarotation behavior of the isolated fraction. Under the same conditions, the isolated solid showed an initial specific rotation of +92.3°, which progressively decreased to a stable equilibrium value of +43.8° after 18 h. The time-dependent decrease confirmed mutarotation and supported the presence of a free anomeric center characteristic of an aldopyranose structure. The lower equilibrium rotation relative to pure D-glucose was consistent with a contribution from the minor levorotatory D-fructose component, as supported by the NMR spectra and shown in Table 3.

Because NMR spectroscopy established D-glucose as the predominant component and provided spectroscopic support for a minor D-fructose component, the lower equilibrium rotation was evaluated using a binary glucose–fructose model. The application of this model was also consistent with the well-established occurrence of glucose and fructose as major reducing sugars in date fruit. The strong negative specific rotation of D-fructose (−92.4° at 20 °C) [35] offsets the positive equilibrium specific rotation of D-glucose (+52.7°) [34].

Accordingly, the relative proportions of the two identified hexoses were calculated from the observed equilibrium specific rotation using the following linear combination:

[α]*_obs_* = *x*([*α*]*_glucose_*) + (1 − *x*) ([*α*]*_fructose_*)

43.8 = 52.7 *x* + (−92.4) (1 − *x*)

43.8 = 52.7 *x* − 92.4 + 92.4 *x*

43.8 = 145.1*x* − 92.4

136.2 = 145.1 *x*

*x* = 136.2/145.1

*x* = 0.938

The calculated value of *x* = 0.938 corresponded to an estimated composition of 93.8% D-glucose and 6.2% D-fructose, equivalent to an approximate D-glucose/D-fructose ratio of 15.1:1 under the applied binary model. Accordingly, these proportions should be regarded as model-based estimates rather than an independent absolute compositional determination. Thus, LC–MS confirmed the presence of hexose species, whereas identification of the major and minor components was based primarily on NMR spectroscopy and their relative proportions were estimated from the polarimetric data. The estimated composition was also consistent with the substantially greater intensities of the D-glucose NMR signals relative to the weak signals attributed to the minor D-fructose component and with the known occurrence of glucose and fructose in date fruit [3].

From a food-analysis perspective, this simple ethanol-based procedure provides a direct approach for obtaining a glucose-rich fraction from the complex date-fruit matrix without chromatographic purification. Further quantitative sugar-separation and solid-state analyses would be useful to define its composition, recovery, and potential applications more comprehensively.

## 4. Conclusions

In this study, a white α-D-glucopyranose-rich fraction was precipitated and isolated from ethanolic extracts of *Khalas* date fruit without chromatographic purification. The isolation procedure was reproduced using three independent date-fruit batches collected over a five-year period. The mean isolated yield was 0.2009 ± 0.0062% (*w*/*w*), demonstrating limited batch-to-batch variation and supporting the reproducibility of the precipitation-based isolation procedure. This value represents the yield of the selectively precipitated fraction and should not be interpreted as the total sugar extraction efficiency or glucose recovery from the date fruit. The isolated fraction was characterized using melting-point determination, TLC, polarimetric mutarotation analysis, ^1^H and ^13^C NMR spectroscopy, DEPT-135 NMR, and LC–MS. The combined analytical results established α-D-glucose as the predominant monosaccharide and confirmed its expected mutarotation toward the β-anomer in aqueous solution. LC–MS confirmed the presence of hexose species but, as expected for constitutional isomers, did not independently distinguish D-glucose from D-fructose. NMR spectroscopy identified D-glucose as the predominant component and provided spectroscopic support for a minor D-fructose component. Following identification of these two hexoses, polarimetric analysis based on their opposite specific rotations yielded a model-based estimate of 93.8% D-glucose and 6.2% D-fructose under the applied binary glucose–fructose model. Overall, the present findings demonstrate the reproducible direct ethanol-induced isolation of an α-D-glucopyranose-rich solid from Khalas date fruit without prior aqueous extraction, together with anomer-specific NMR characterization showing a pronounced predominance of the α-anomer in the isolated material and its subsequent mutarotation toward the β-anomer in aqueous solution.

## Figures and Tables

**Table 1:** Reproducibility of precipitation-based isolation across three independent *Khalas* date fruit batches.

Batch	Collection Date	Date Fruit Mass (g)	Isolated Fraction (g)	Yield (% *w*/*w*)
1	September 2016	2000	4.013	0.2007
2	September 2017	2000	3.897	0.1949
3	September 2021	2000	4.145	0.2073
Mean ± SD	—	—	4.018 ± 0.124	0.2009 ± 0.0062

Note: Yield was calculated as the mass of the dried isolated fraction divided by the initial date-fruit mass × 100. The mean and sample standard deviation were calculated from three independent batches (n = 3). The NMR profiles were consistent with a glucose-rich fraction, while LC–MS confirmed the presence of hexose species.

**Table 2:** TLC comparison of the isolated fraction and standard monosaccharides based on *R_f_* values and color development after *p*-anisaldehyde staining.

Lane	Compound	*R_f_* Value	Color After PAA Staining	Comment
X	Isolated fraction	0.36	Light green	*R_f_* and color are most consistent with D-glucose
Glu	D-Glucose	0.35	Green	Closest match to compound X in both *R_f_* and color
Fru	D-Fructose	0.37	Dark green	Similar *R_f_* to X, but clearly darker color development
Mann	D-Mannose	0.31	Dark yellowish-green	Lower *R_f_* and different color from compound X
Galc	D-Galactose	0.25	Yellowish-green	Considerably lower *R_f_* and different color from compound X
Ribo	D-Ribose	0.52	Green	Considerably higher *R_f_* than compound X

R*_f_* = distance traveled by the center of the analyte spot divided by the distance traveled by the solvent front, both measured from the baseline. The estimated uncertainty in the R*_f_* values was approximately ±0.02. PAA: *p*-anisaldehyde staining reagent.

**Table 3:** Specific rotation of commercial D-glucose and the isolated fraction under identical experimental conditions.

Sample	Time (h)	Observed Rotation α	Specific Rotation ${\left[\alpha \right]}_{D}^{20}$
Commercial D-glucose	0	+1.065°	+106.5°
Commercial D-glucose	18	+0.518°	+51.8°
Isolated solid	0	+0.923°	+92.3°
Isolated solid	18	+0.438°	+43.8°

Measurements were performed in H_2_O at 20 °C using a 1 dm polarimeter tube at 589 nm. Sample concentration was 1.0 g/100 mL (1% *w*/*v*).

## Data Availability

The data supporting the findings of this study are provided within the article. Additional data are available from the corresponding author upon reasonable request.

## Supplementary Materials

The following supporting information can be downloaded at https://www.mdpi.com/article/10.3390/foods15203632/s1: Figures S1–S32, LC–MS and NMR spectra and polarimeter images; Table S1, optical rotation measurements during mutarotation; Table S2, batch-to-batch reproducibility.
