# Supplementary material for: Reproducible Ethanol-Induced Precipitation and Systematic Characterization of an α-D-Glucopyranose-Rich Solid from Khalas Date Fruit (Phoenix dactylifera L.)
Source: Foods. 2026 Oct 13;15(20):3632. doi: 10.3390/foods15203632 (PMC13703318; doi:10.3390/foods15203632)
Supplement: Supplementary file 1 [file foods-15-03632-s001.zip › Foods SI 13.09.2026.pdf]

Supplementary Material

for

Reproducible Spontaneous Isolation and Systematic Characterization of an α-D-Glucopyranose-Rich Crystalline Solid from *Khalas* Date Fruit (*Phoenix dactylifera* L.)

Reem A. Al Alawi[1] and Younis Baqi[1,*]

[1] Department of Chemistry, College of Science, Sultan Qaboos University, Al Khod, P.O. Box 36, Muscat 123, Oman (R.A.A.A.: r.alalawi2@squ.edu.om)

*** Correspondence:** baqi@squ.edu.om; Tel.: +968-2414-1473

Table of contents

**Contents**	**Page**
**Figure S1.** LC–MS Spectrum of the isolated α-D-glucopyranose-rich fraction **(Batch 1).**	S5
**Figure S2.** LC–MS Spectrum of the isolated α-D-glucopyranose-rich fraction **(Batch 2).**	S6-S7
**Figure S3.** LC–MS Spectrum of the isolated α-D-glucopyranose-rich fraction **(Batch 3).**	S8-S9
**Figure S4.** ^{1}H-NMR Spectrum (DMSO-d_6) of the isolated α-D-glucopyranose-rich fraction **(Batch 1).**	S10
**Figure S5.** Expanded ^{1}H NMR Spectrum (DMSO-d_6) of the isolated α-D-glucopyranose-rich fraction **(Batch 1).**	S11
**Figure S6.** ^{13}C NMR Spectrum (DMSO-d_6) of the isolated α-D-glucopyranose-rich fraction **(Batch 1).**	S12

**Figure S7.** DEPT-135 Spectrum (DMSO-d_6) of the isolated α-D-glucopyranose-rich fraction **(Batch 1).**	S13
**Figure S8.** ^{1}H-NMR Spectrum (DMSO-d_6) of the isolated α-D-glucopyranose-rich fraction **(Batch 2).**	S14
**Figure S9.** Expanded ^{1}H NMR Spectrum (DMSO-d_6) of the isolated α-D-glucopyranose-rich fraction **(Batch 2).**	S15
**Figure S10.** ^{13}C NMR Spectrum (DMSO-d_6) of the isolated α-D-glucopyranose-rich fraction **(Batch 2).**	S16
**Figure S11.** DEPT-135 Spectrum (DMSO-d_6) of the isolated α-D-glucopyranose-rich fraction **(Batch 2).**	S17
**Figure S12.** ^{1}H-NMR Spectrum (DMSO-d_6) of the isolated α-D-glucopyranose-rich fraction **(Batch 3).**	S18
**Figure S13.** Expanded ^{1}H NMR Spectrum (DMSO-d_6) of the isolated α-D-glucopyranose-rich fraction **(Batch 3).**	S19
**Figure S14.** ^{13}C NMR Spectrum (DMSO-d_6) of the isolated α-D-glucopyranose-rich fraction **(Batch 3).**	S20
**Figure S15.** DEPT-135 Spectrum (DMSO-d_6) of the isolated α-D-glucopyranose-rich fraction **(Batch 3).**	S21
**Figure S16.** ^{1}H-NMR Spectrum (DMSO-d_6) of the isolated α-D-glucopyranose-rich fraction at time zero (t_0).	S22
**Figure S17.** Expanded ^{1}H NMR Spectrum (DMSO-d_6) of the isolated α-D-glucopyranose-rich fraction at time zero (t_0).	S23
**Figure S18.** ^{13}C NMR Spectrum (DMSO-d_6) of the isolated α-D-glucopyranose-rich fraction at time zero (t_0).	S24

**Figure S19.** DEPT-135 Spectrum (DMSO-d_6) of the isolated α-D-glucopyranose-rich fraction at time zero (t_0).	S25
**Figure S20.** ^{1}H-NMR Spectrum (DMSO-d_6) of the isolated α-D-glucopyranose-rich fraction after 24 hours (t_{24}).	S26
**Figure S21.** Expanded ^{1}H NMR Spectrum (DMSO-d_6) of the isolated α-D-glucopyranose-rich fraction after 24 hours (t_{24}).	S27
**Figure S22.** ^{13}C NMR Spectrum (DMSO-d_6) of the isolated α-D-glucopyranose-rich fraction after 24 hours (t_{24}).	S28
**Figure S23.** DEPT-135 Spectrum (DMSO-d_6) of the isolated α-D-glucopyranose-rich fraction after 24 hours (t_{24}).	S29
**Figure S24.** ^{1}H-NMR Spectrum (D_2O) of the isolated α-D-glucopyranose-rich fraction at time zero (t_0).	S30
**Figure S25.** Expanded ^{1}H NMR Spectrum (D_2O) of the isolated α-D-glucopyranose-rich fraction at time zero (t_0).	S31
**Figure S26.** ^{13}C NMR Spectrum (D_2O) of the isolated α-D-glucopyranose-rich fraction at time zero (t_0).	S32
**Figure S27.** DEPT-135 Spectrum (D_2O) of the isolated α-D-glucopyranose-rich fraction at time zero (t_0)	S33
**Figure S28.** ^{1}H-NMR Spectrum (D_2O) of the isolated α-D-glucopyranose-rich fraction after 24 hours (t_{24}).	S34
**Figure S29.** Expanded ^{1}H NMR Spectrum (D_2O) of the isolated α-D-glucopyranose-rich fraction after 24 hours (t_{24}).	S35
**Figure S30.** ^{13}C NMR Spectrum (D_2O) of the isolated α-D-glucopyranose-rich fraction after 24 hours (t_{24}).	S36

**Figure S31.** DEPT-135 Spectrum (D_2O) of the isolated α-D-glucopyranose-rich fraction after 24 hours (t_{24}).	S37
**Table S1.** Optical rotation measurements of the isolated α-D-glucopyranose-rich fraction recorded over time during mutarotation in water at 20 °C.	S38
**Figure S32.** Representative polarimeter display images recorded immediately after dissolution (0 h), (1 h 50 min), (3 h) and after 18 h to monitor changes in optical rotation during mutarotation under the experimental conditions described in the main manuscript.	S39
**Table S2.** Reproducibility of the isolated α-D-glucopyranose-rich fraction across independent experimental batches.	S40

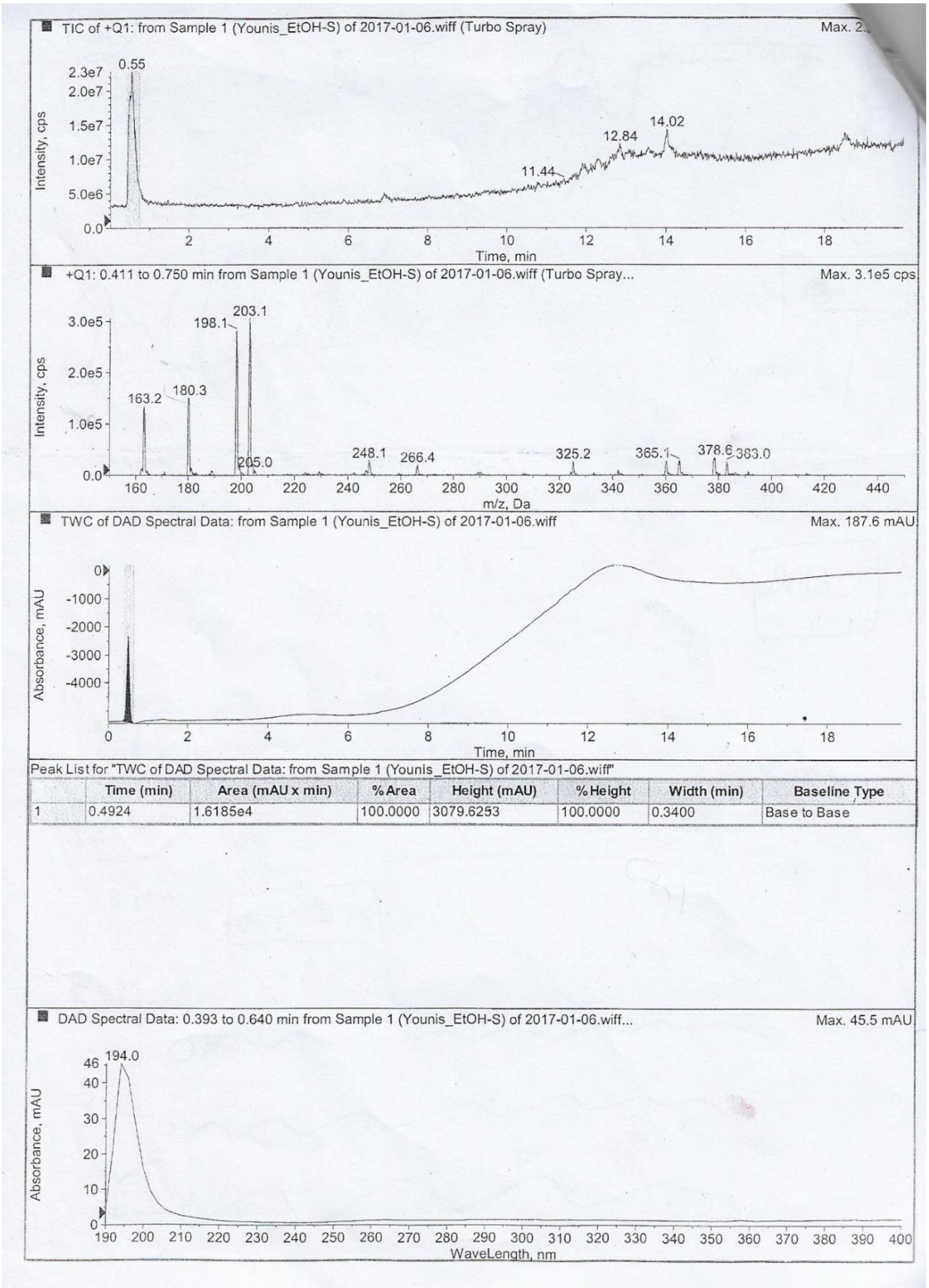

	Time (min)	Area (mAU x min)	%Area	Height (mAU)	%Height	Width (min)	Baseline Type
1	0.4924	1.6185e4	100.0000	3079.6253	100.0000	0.3400	Base to Base

Figure S1. LC–MS Spectrum of the isolated α-D-glucopyranose-rich fraction (**Batch 1**).

**Sequence Name:**	SingleSample		
**Data file:**	EtOH-WS.dx	**Project Name:**	Single Quad
**Sample name:**	EtOH-WS	**Operator :**	SYSTEM
**Instrument:**	Single Quad	**Acquired on:**	2022-02-14 15:04:48+01:00
**Inj. volume:**	2.000	**Location:**	P1-D11
**Acq. method:**	00-new_wasser-acn_standard_100-1500_BD-20min.amx		
**Processing method:**	MS_standard_plot.pmx		

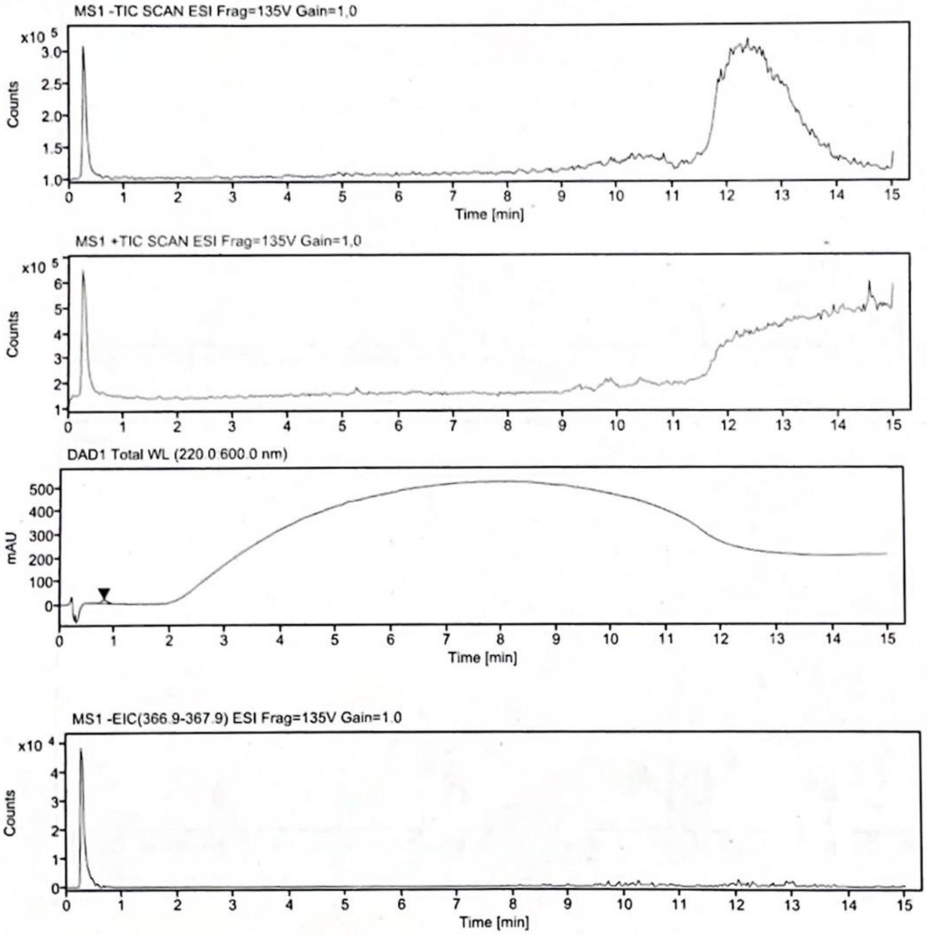

Signal: DAD1 Total WL (220.0:600.0 nm)

RT [min]	Peak MS Base Peak m/z	Area	Area%	Max Peak%	Height
0.807		115.2264	100.0000	100.000	14.697
	Sum	115.2264			

Signal: MS1 +TIC SCAN ESI Frag=135V Gain=1,0

RT [min]	Peak MS Base Peak m/z	Area	Area%	Max Peak%	Height
0.270	198.200	3107223.3643	100.0000	100.000	534342.906
	Sum	3107223.3643			

Signal: MS1 -TIC SCAN ESI Frag=135V Gain=1,0

RT [min]	Peak MS Base Peak m/z	Area	Area%	Max Peak%	Height
0.281	367.200	989414.6288	100.0000	100.000	211132.496
	Sum	989414.6288			

MS1 +TIC SCAN ESI Frag=135V Gain=1,0RT:0.270274480832501min (Subtracted)

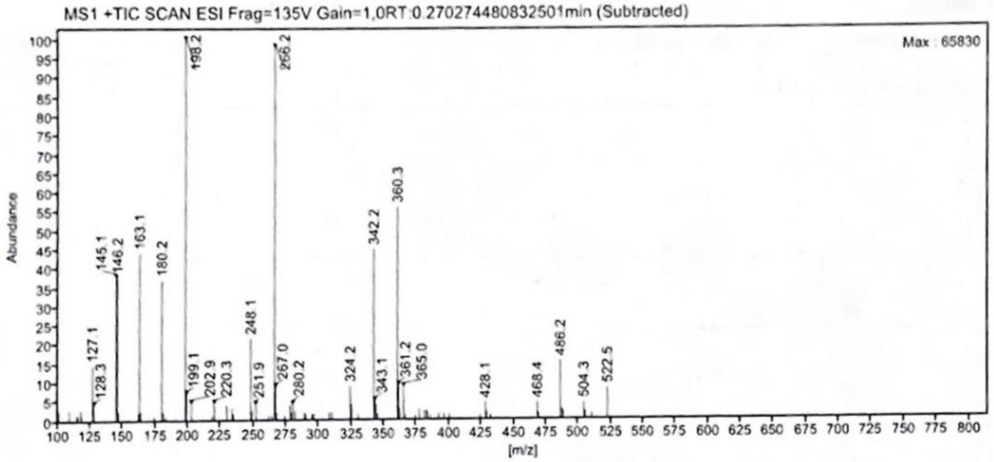

MS1 -TIC SCAN ESI Frag=135V Gain=1,0RT:0.281371333716057min (Subtracted)

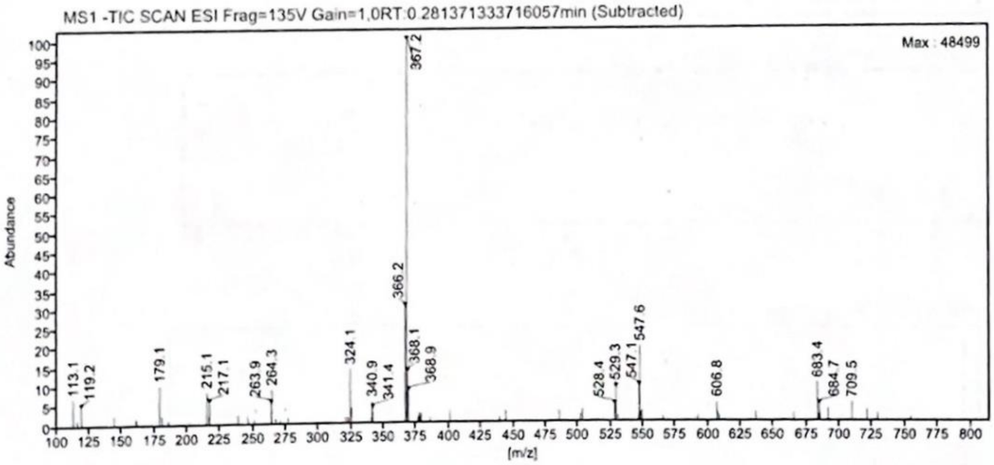

D:\CDSProjects\Default\0-standardplot+EIC.rdl [Rev. 5213] | Printed: 2022-02-15 06:47:45+01:00 | Page 2 of 2

Figure S2. LC–MS Spectrum of the isolated α-D-glucopyranose-rich fraction (**Batch 2**).

**Sequence Name:**	SingleSample		
**Data file:**	EtOH-s.dx	**Project Name:**	Single Quad
**Sample name:**	EtOH-s	**Operator :**	SYSTEM
**Instrument:**	Single Quad	**Acquired on:**	2025-09-10 12:48:15+02:00
**Inj. volume:**	2.000	**Location:**	P2-E9
**Acq. method:**	00-new_wasser-acn_standard_100-1000_BD-20min.amx		
**Processing method:**	MS_standard_plot.pmx		

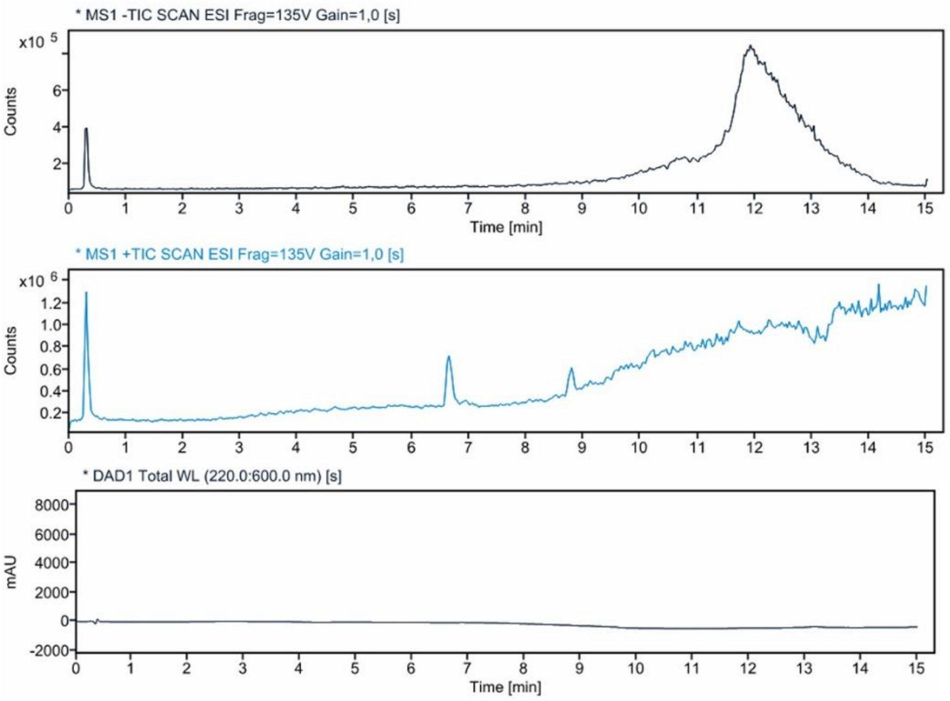

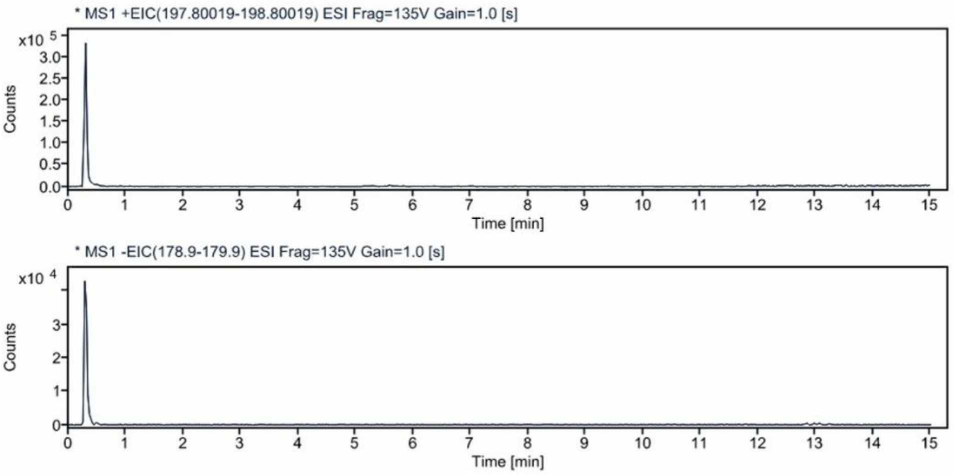

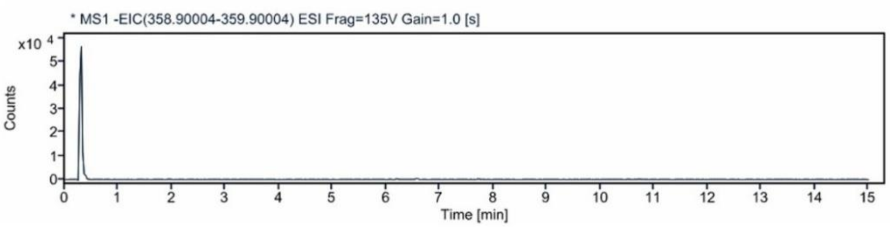

Signal: * MS1 +TIC SCAN ESI Frag=135V Gain=1,0 [s]

RT [min]	Peak MS Base Peak m/z	Area	Area%	Max Peak%	Height
0.308	198.100	5236399.1062	100.0000	100.000	1160114.703
	Sum	5236399.1062			

Signal: * MS1 -TIC SCAN ESI Frag=135V Gain=1,0 [s]

RT [min]	Peak MS Base Peak m/z	Area	Area%	Max Peak%	Height
0.308	359.200	1521754.2120	100.0000	100.000	361292.171
	Sum	1521754.2120			

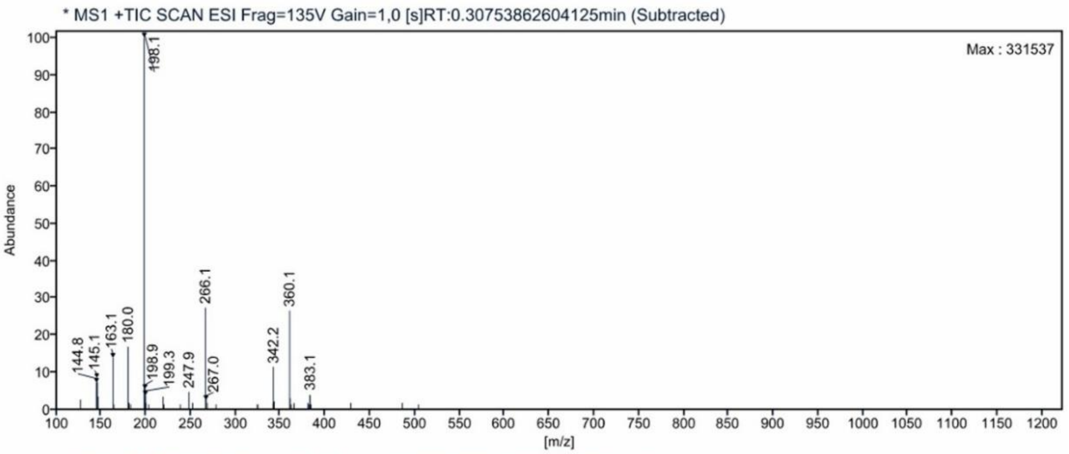

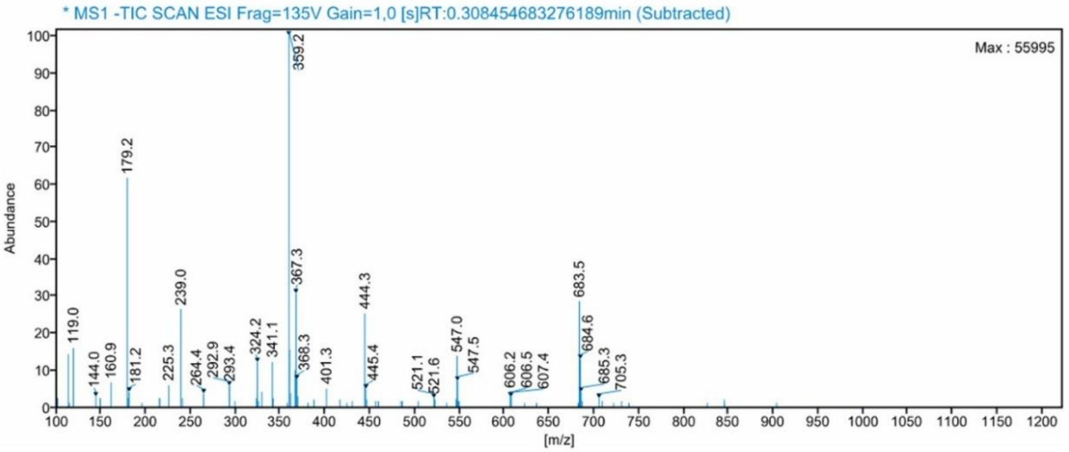

Figure S3. LC–MS Spectrum of the isolated α-D-glucopyranose-rich fraction (**Batch 3**).

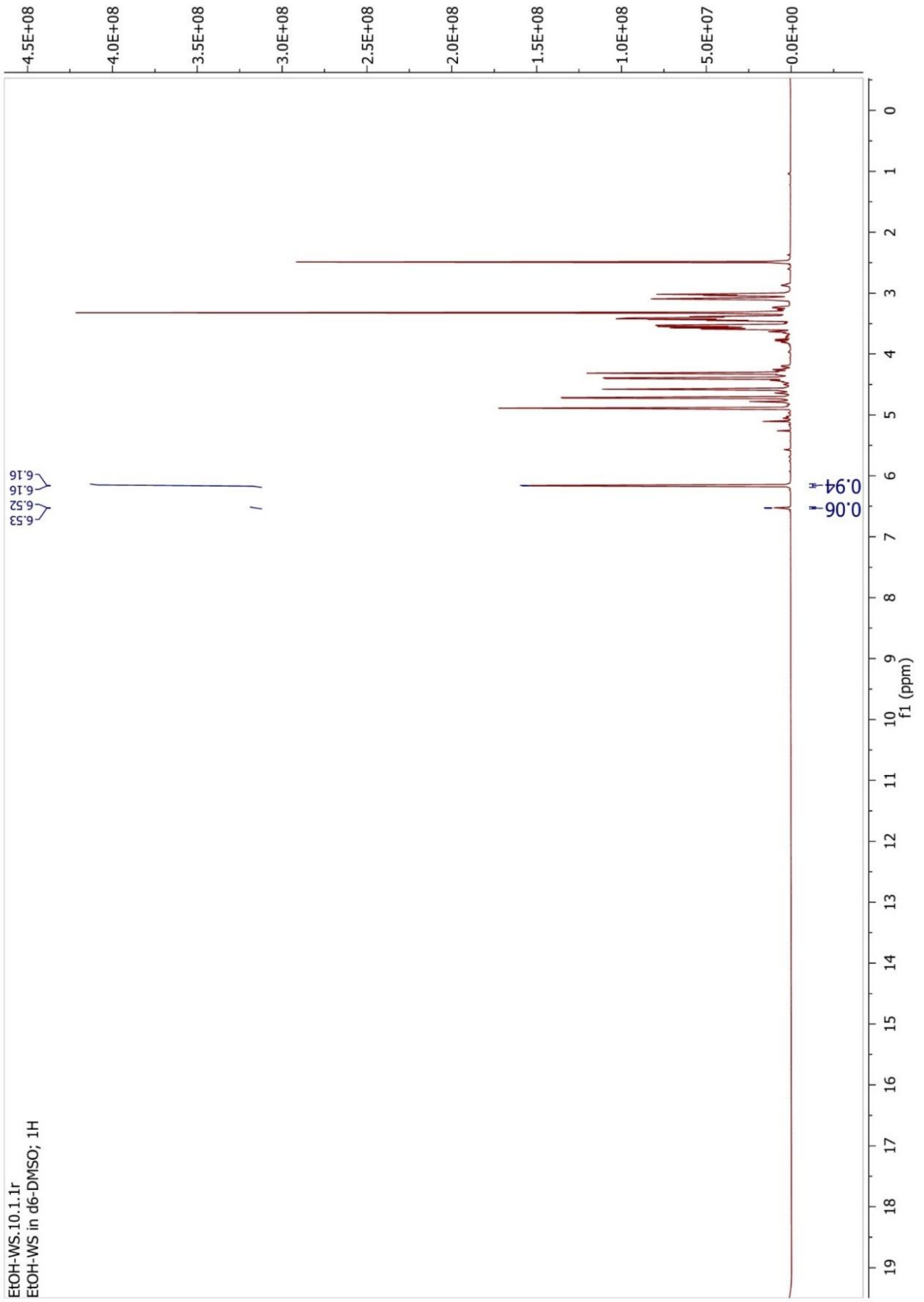

Figure S4. ^{1}H-NMR Spectrum (DMSO-d_6) of the isolated α-D-glucopyranose-rich fraction (**Batch 1**).

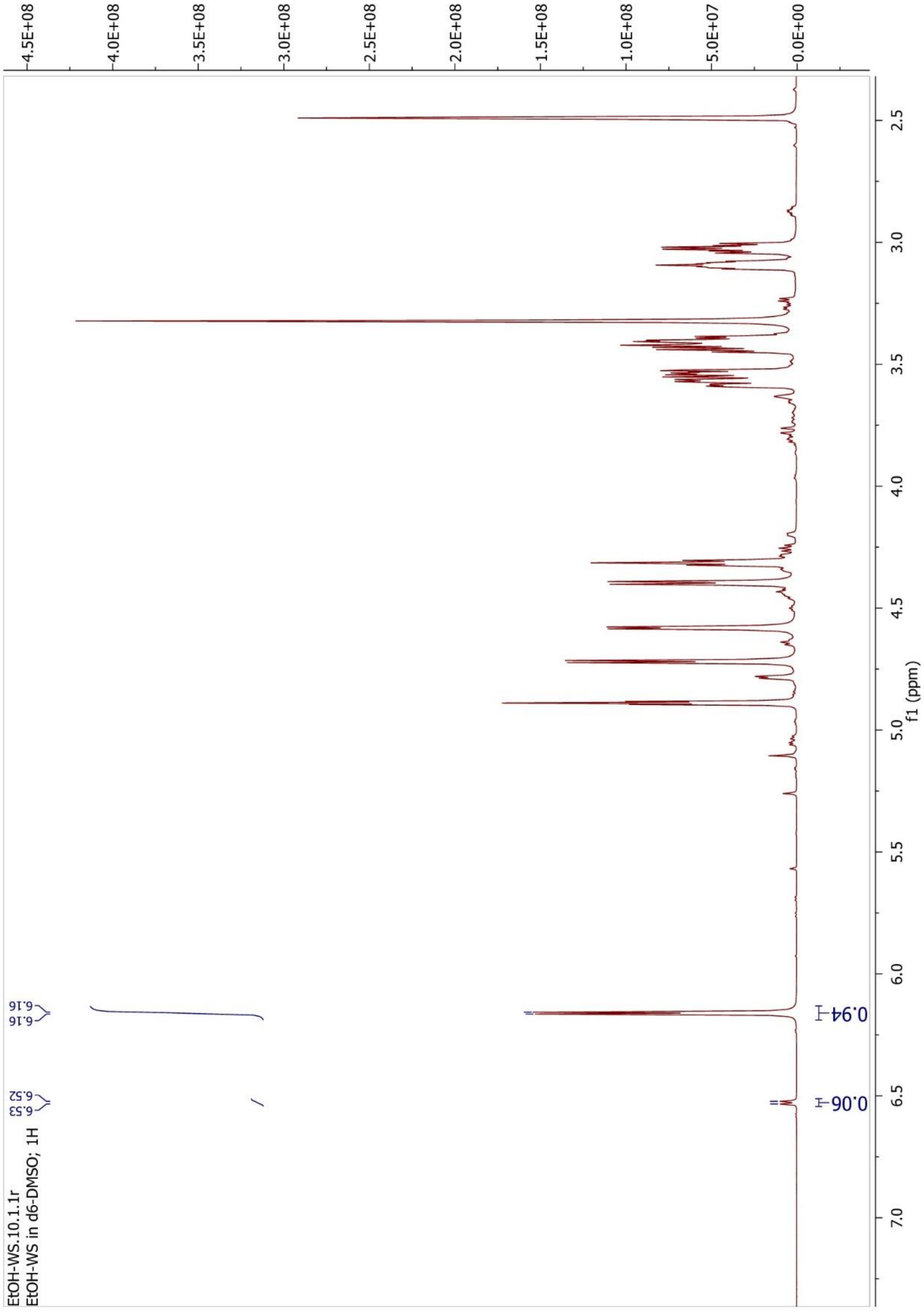

Figure S5. Expanded ^{1}H NMR Spectrum (DMSO-d_6) of the isolated α-D-glucopyranose-rich fraction (**Batch 1**).

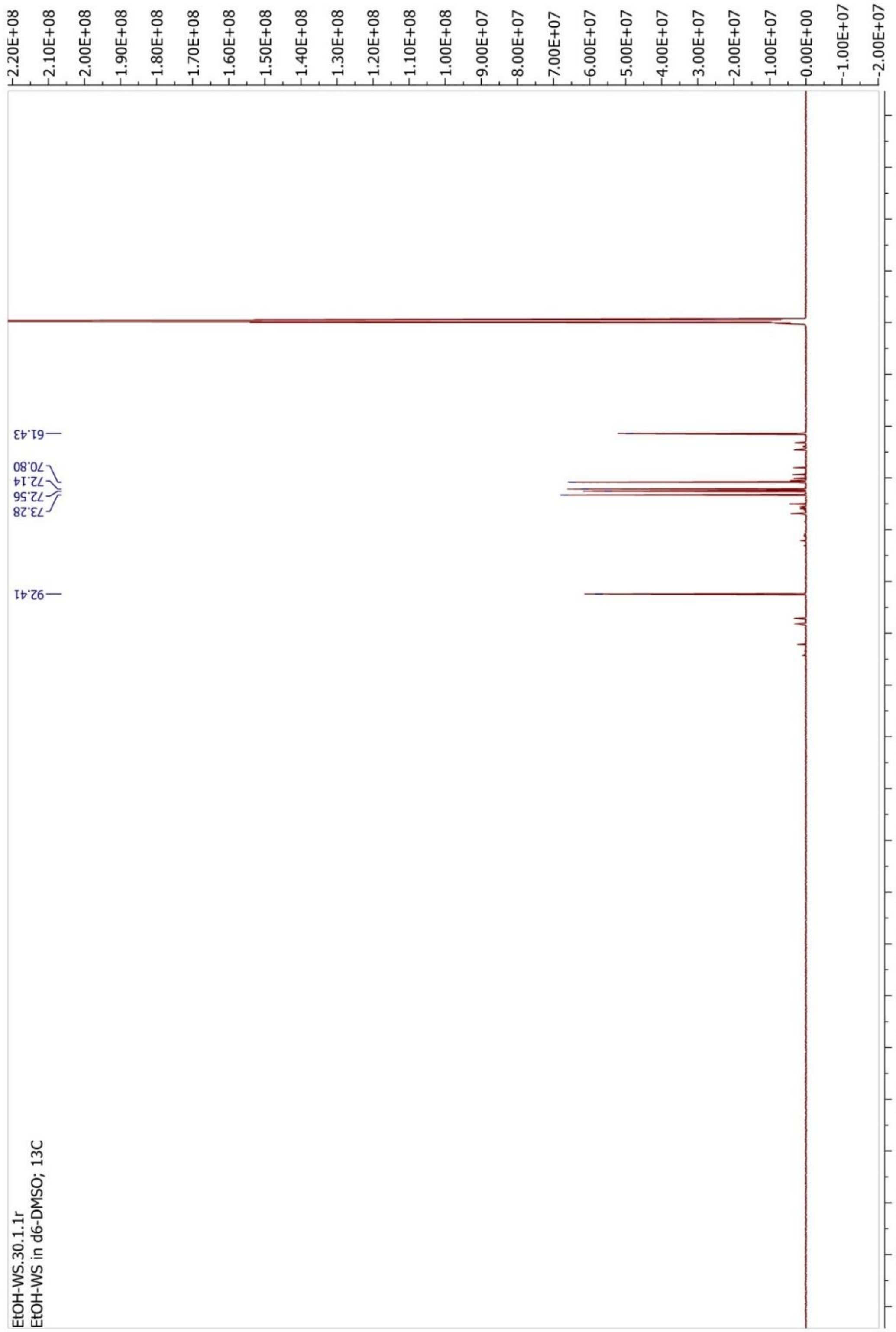

Figure S6. ^{13}C NMR Spectrum (DMSO-d_6) of the isolated α-D-glucopyranose-rich fraction (**Batch 1**).

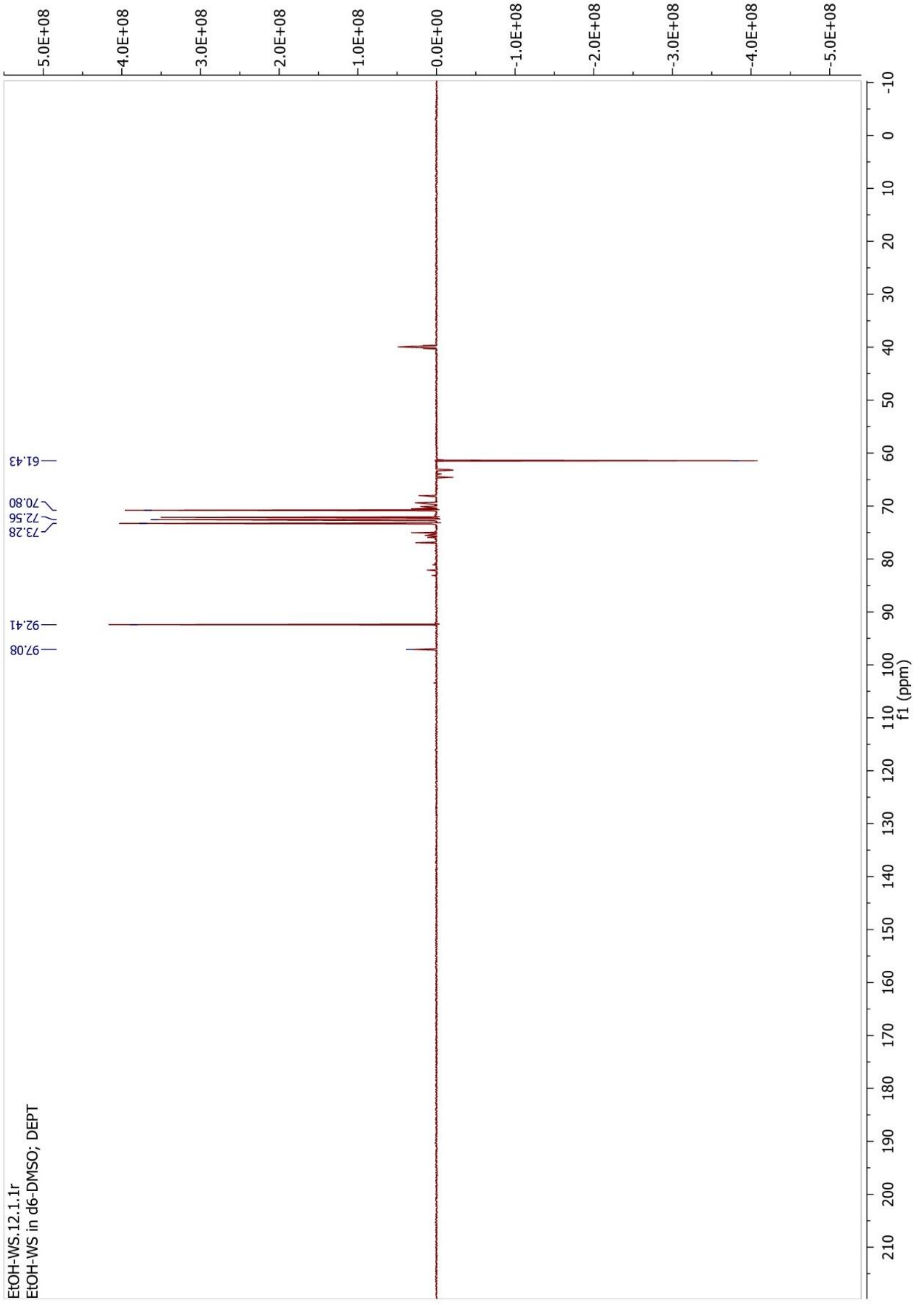

Figure S7. DEPT-135 Spectrum (DMSO-d_6) of the isolated α-D-glucopyranose-rich fraction (**Batch 1**).

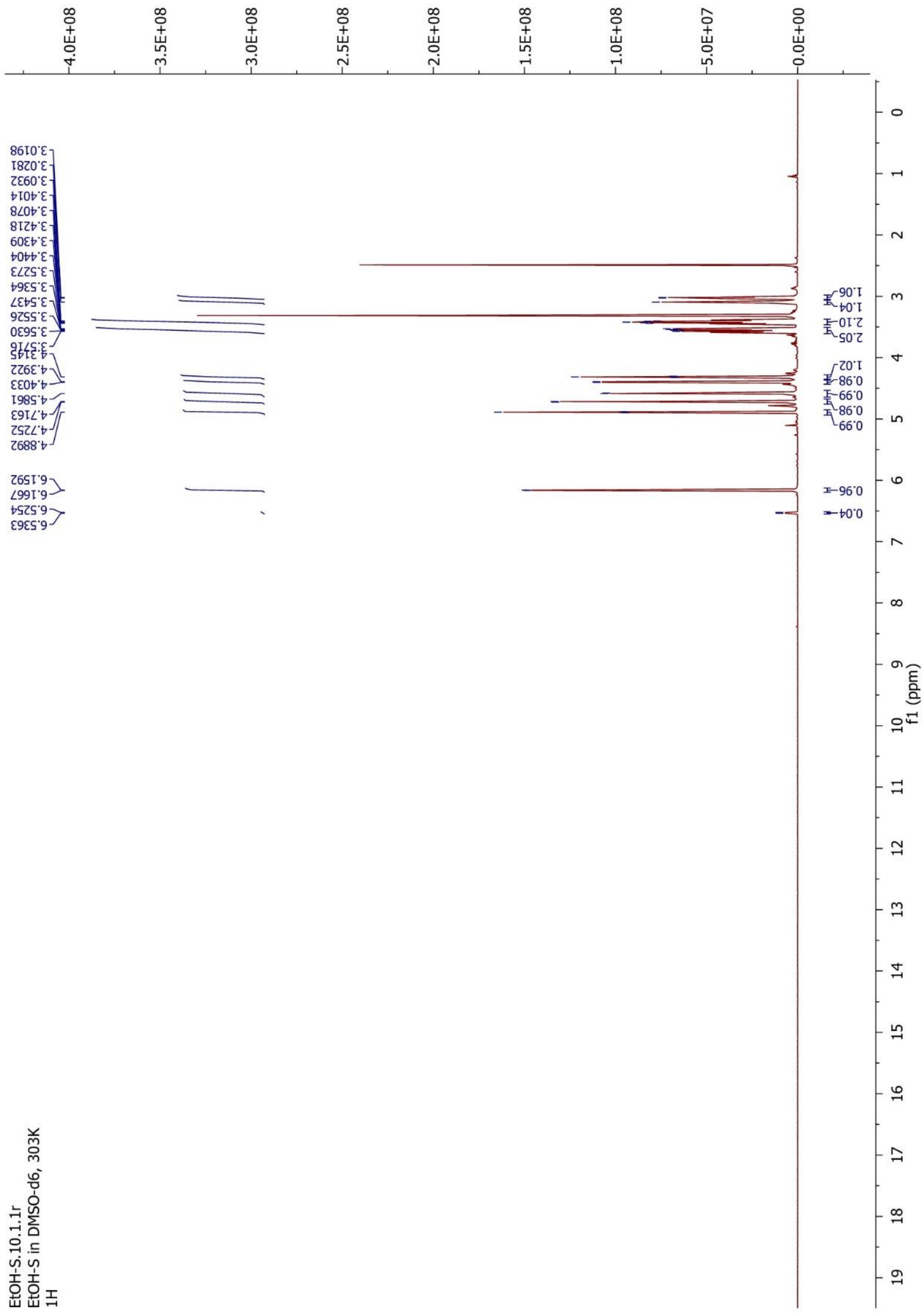

Figure S8. ^{1}H-NMR Spectrum (DMSO-d_6) of the isolated α-D-glucopyranose-rich fraction (**Batch 2**).

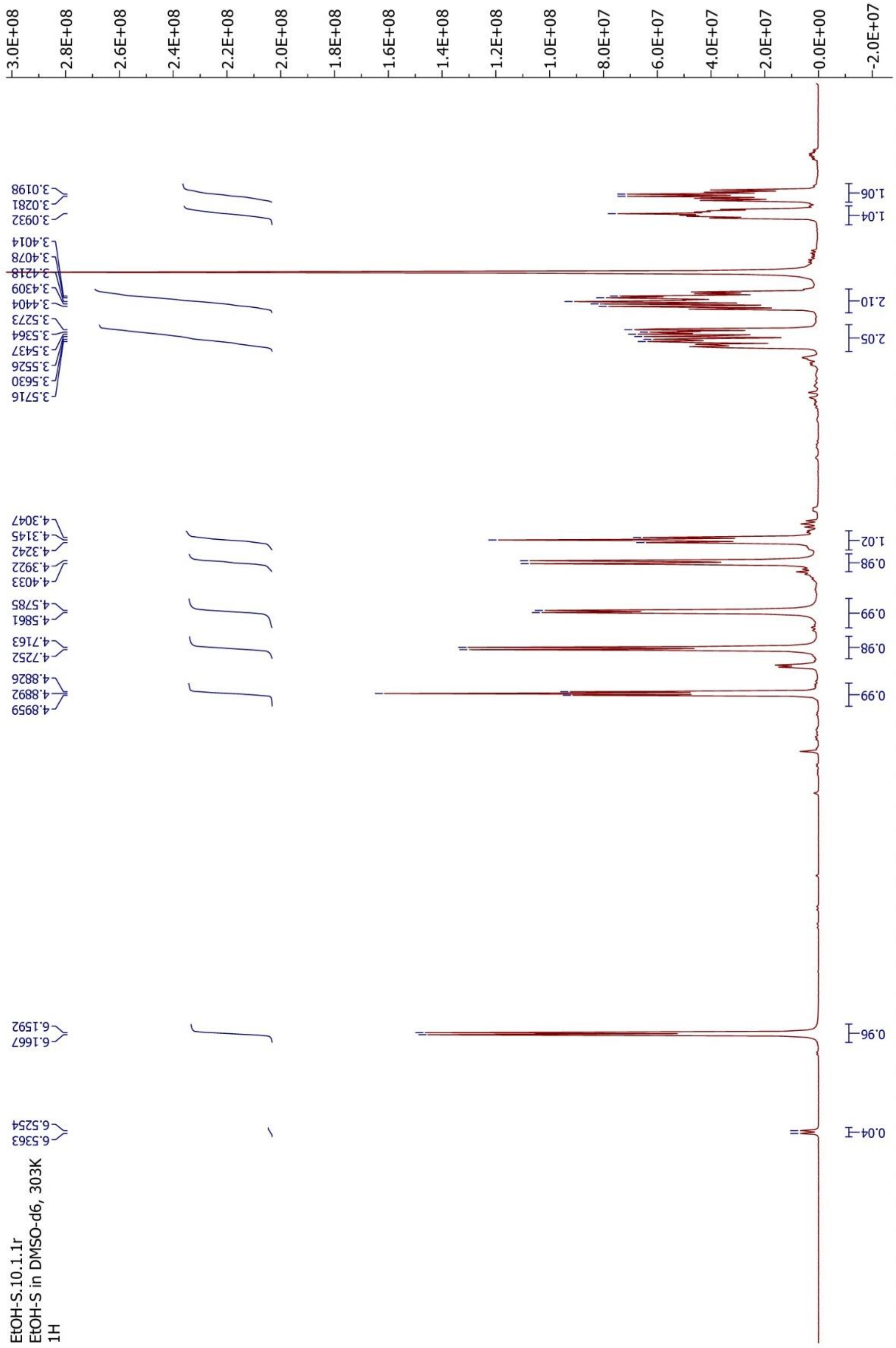

Figure S9. Expanded ^{1}H NMR Spectrum (DMSO-d_6) of the isolated α-D-glucopyranose-rich fraction (**Batch 2**).

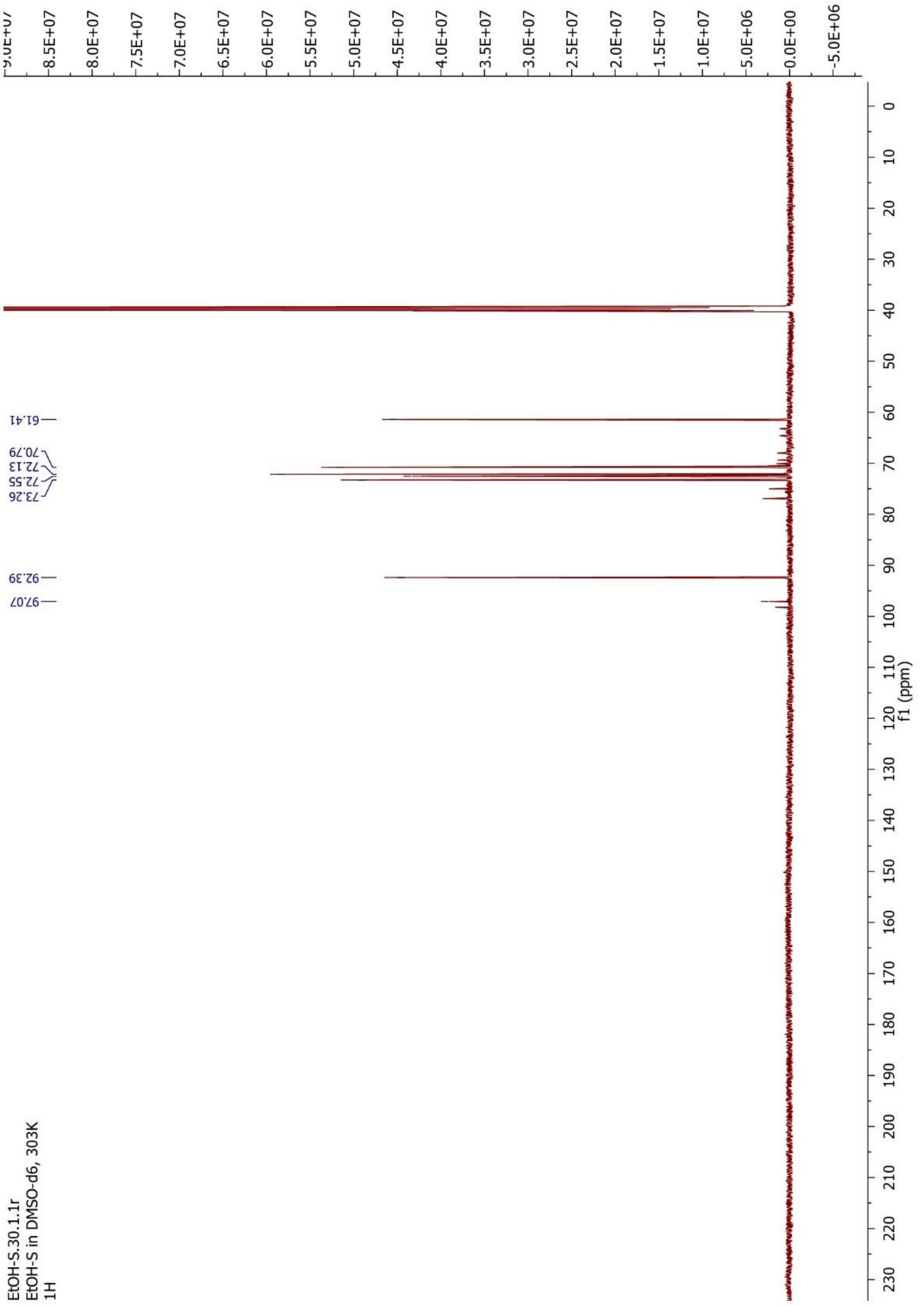

Figure S10. ^{13}C NMR Spectrum (DMSO-d_6) of the isolated α-D-glucopyranose-rich fraction (**Batch 2**).

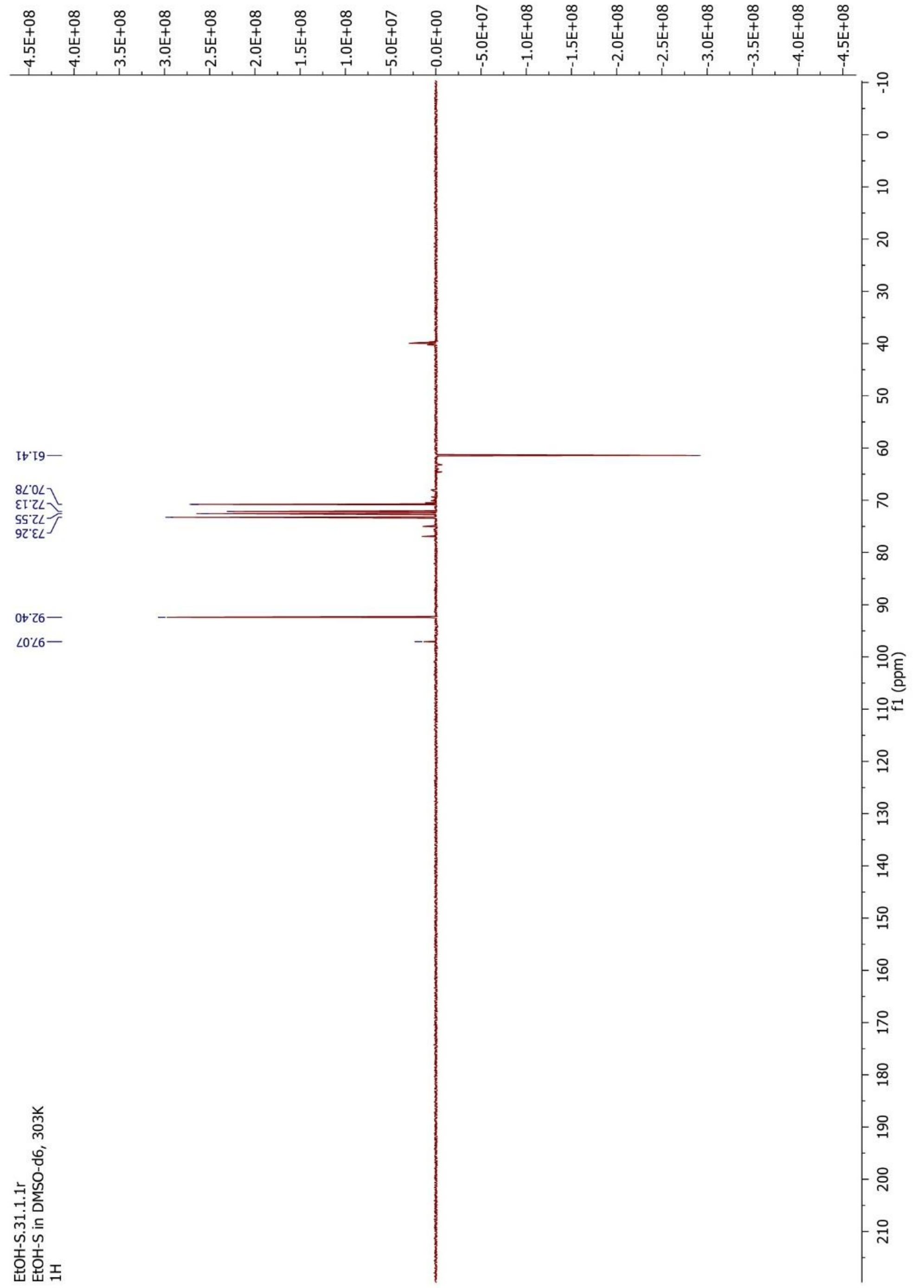

Figure S11. DEPT-135 Spectrum (DMSO-d_6) of the isolated α-D-glucopyranose-rich fraction (**Batch 2**).

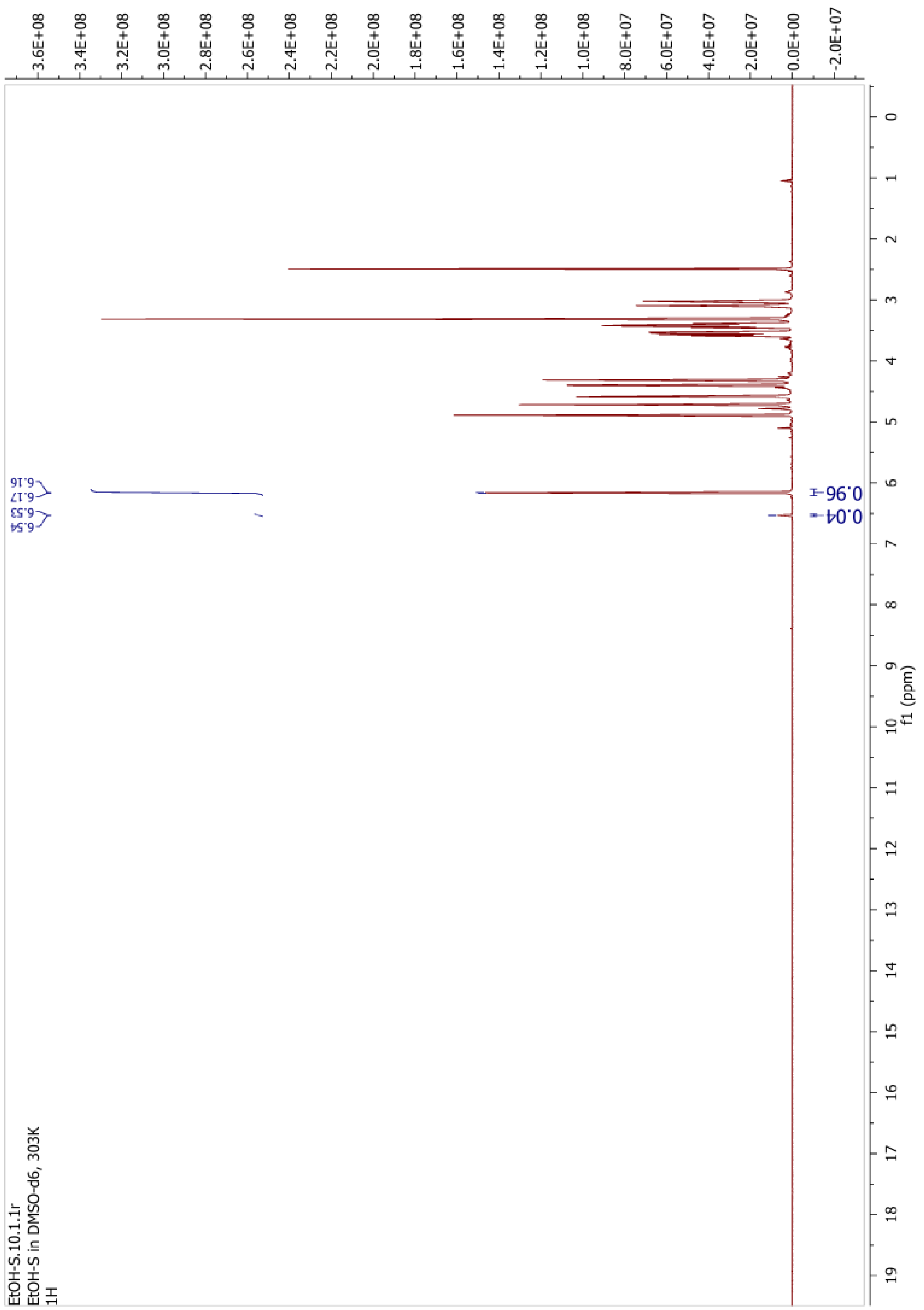

Figure S12. ^{1}H-NMR Spectrum (DMSO-d_6) of the isolated α-D-glucopyranose-rich fraction (**Batch 3**).

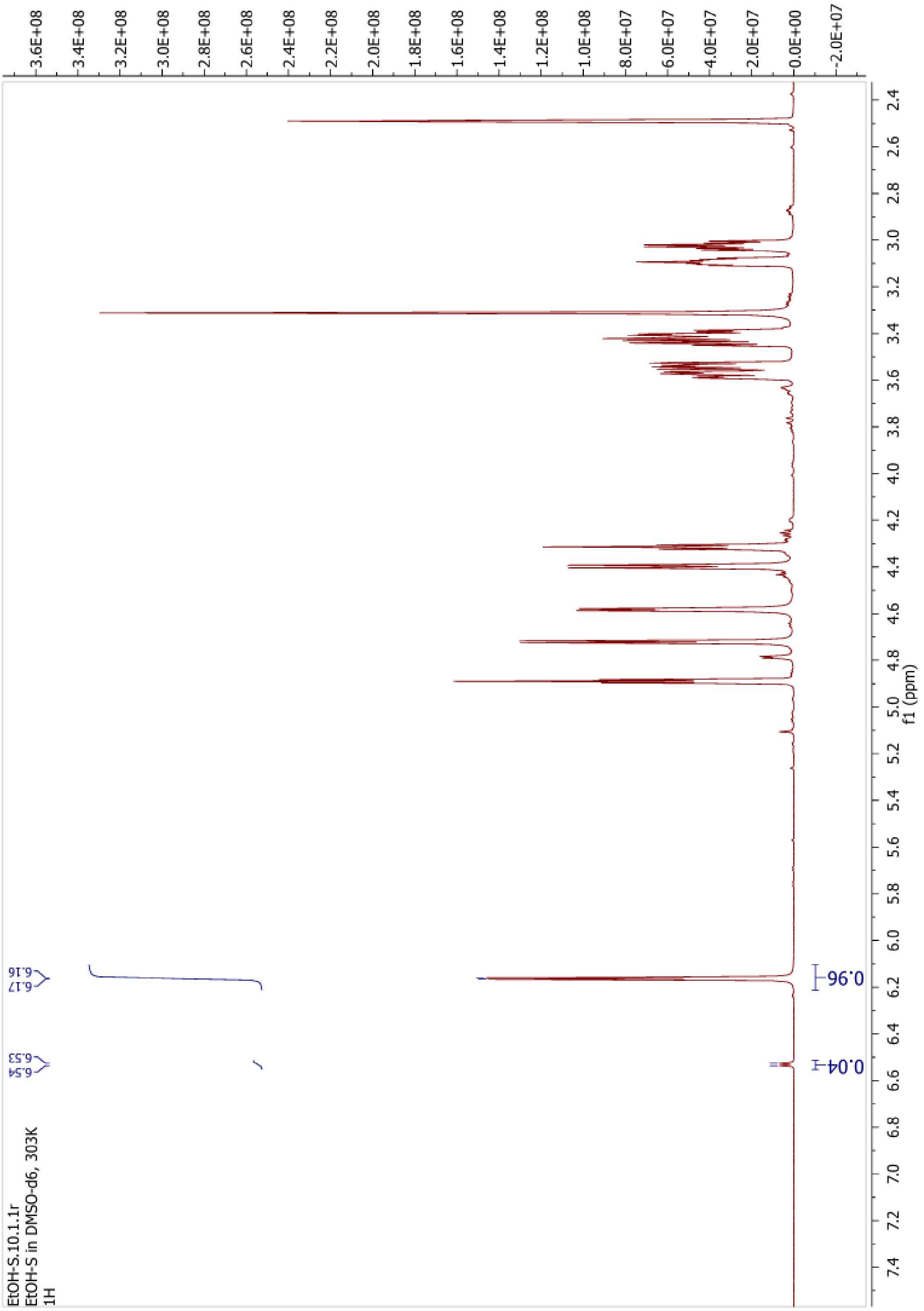

Figure S13. Expanded ^{1}H NMR Spectrum (DMSO-d_6) of the isolated α-D-glucopyranose-rich fraction (**Batch 3**).

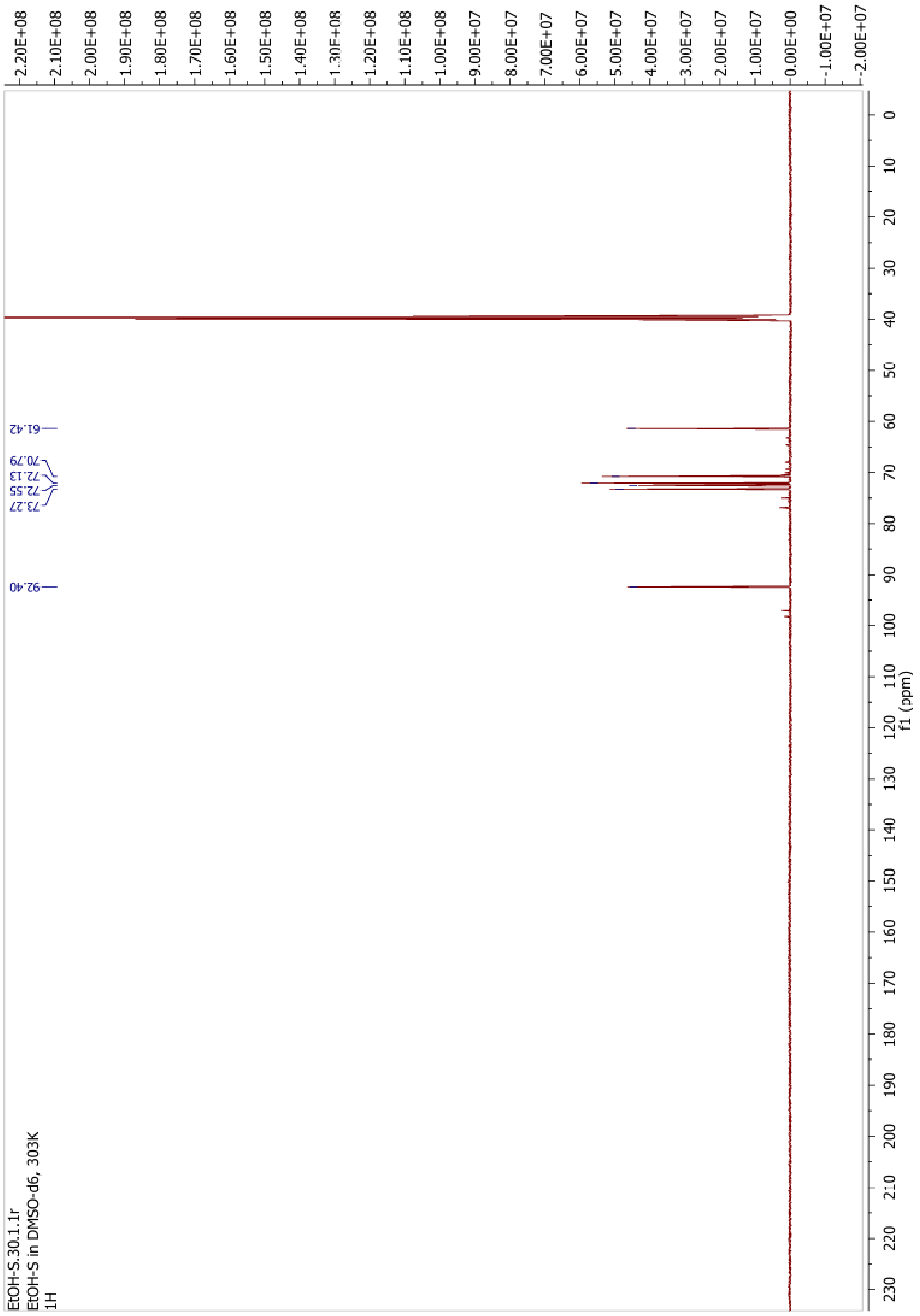

Figure S14. ^{13}C NMR Spectrum (DMSO-d_6) of the isolated α-D-glucopyranose-rich fraction (**Batch 3**).

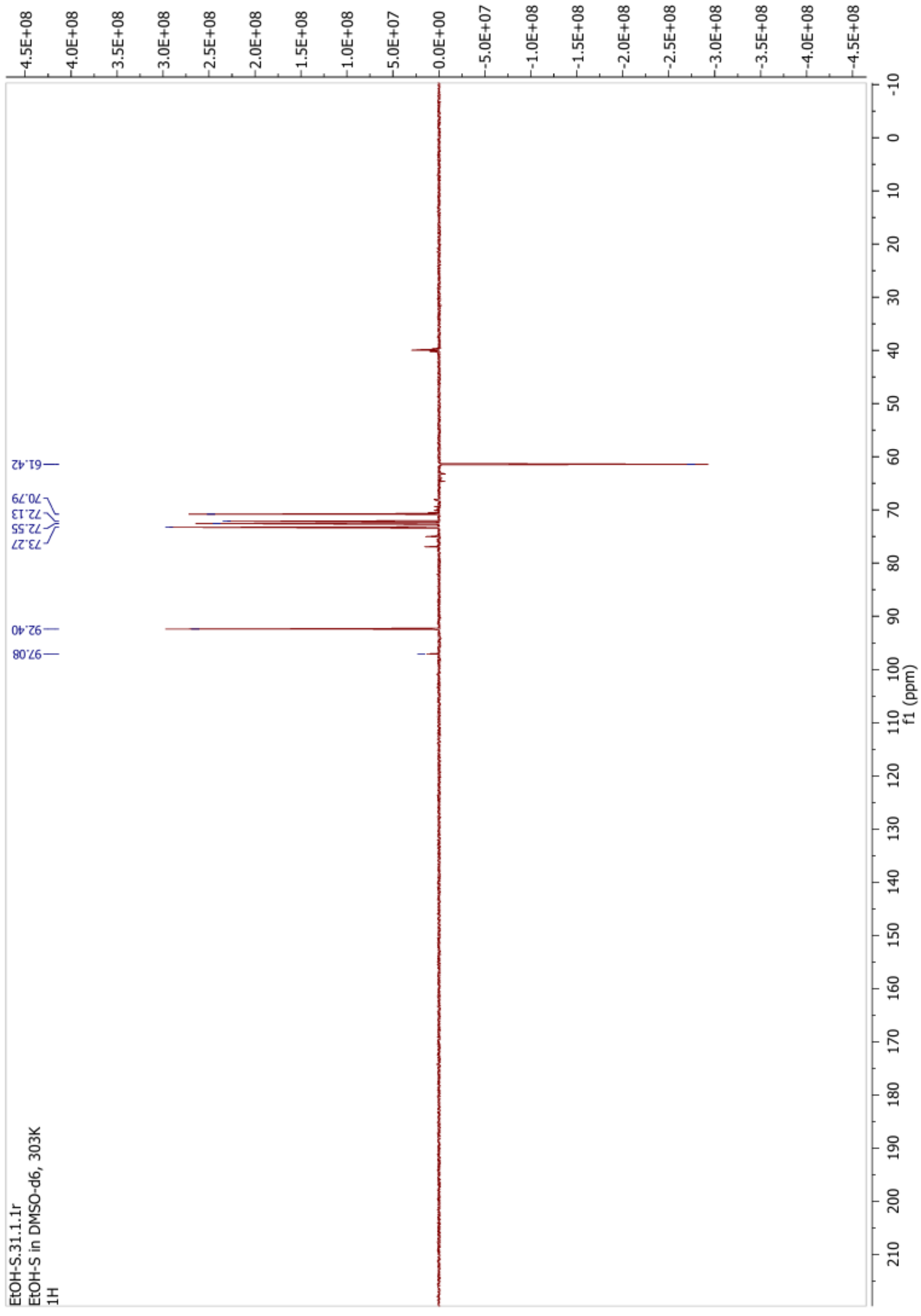

Figure S15. DEPT-135 Spectrum (DMSO-d_6) of the isolated α-D-glucopyranose-rich fraction (**Batch 3**).

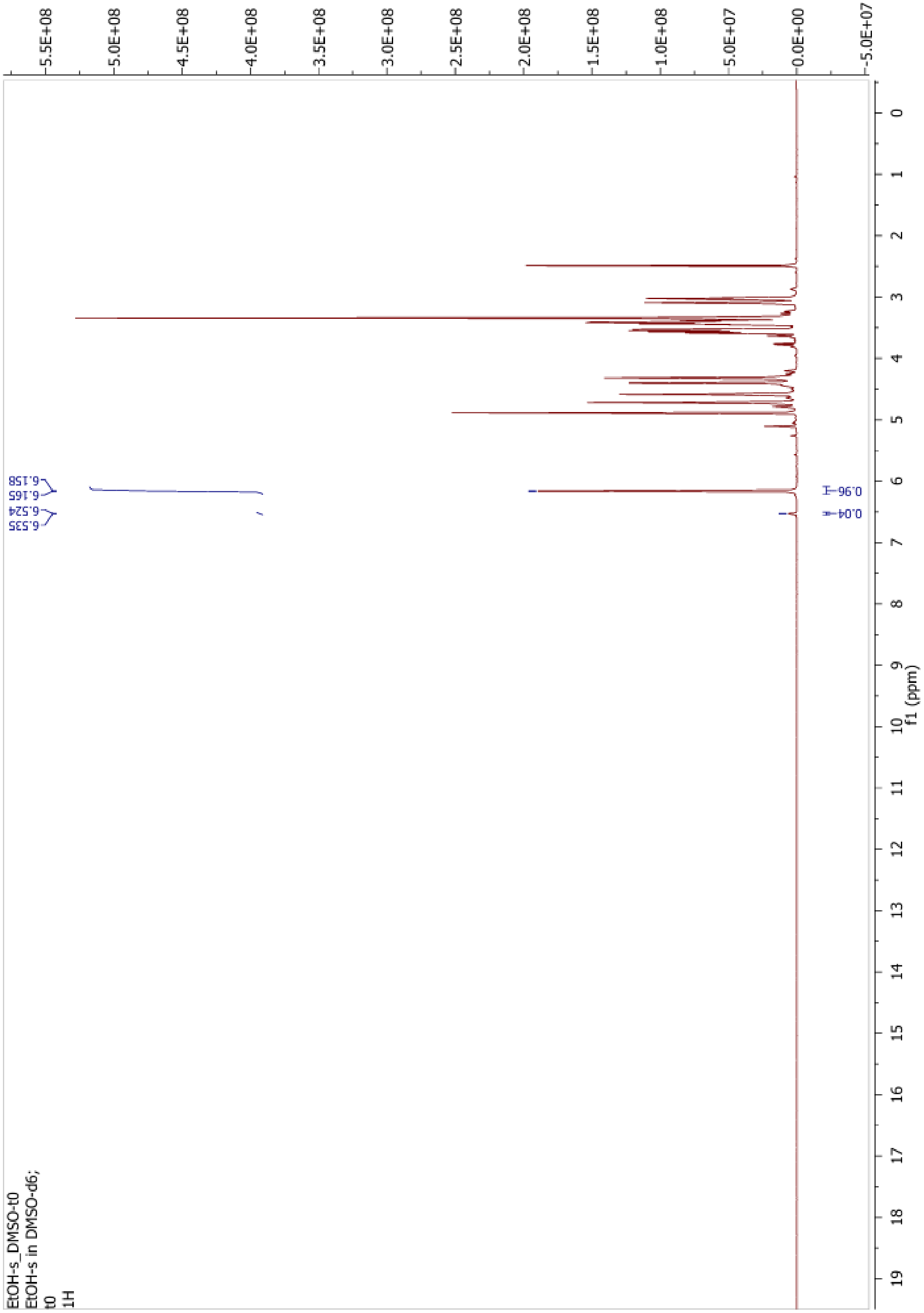

Figure S16. ^{1}H-NMR Spectrum (DMSO-d_6) of the isolated α-D-glucopyranose-rich fraction at time zero (t_0).

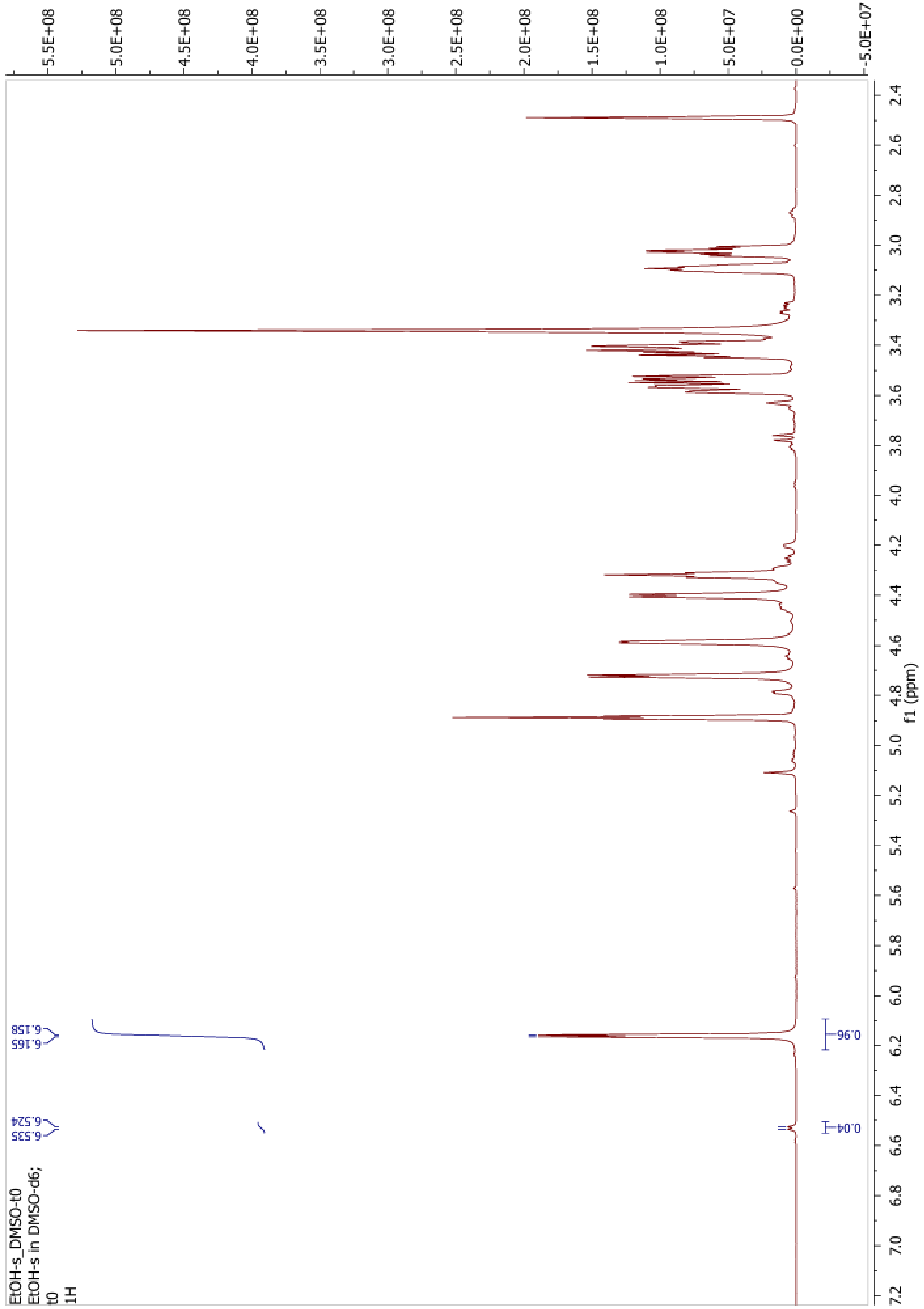

Figure S17. Expanded ^{1}H NMR Spectrum (DMSO-d_6) of the isolated α-D-glucopyranose-rich fraction at time zero (t_0).

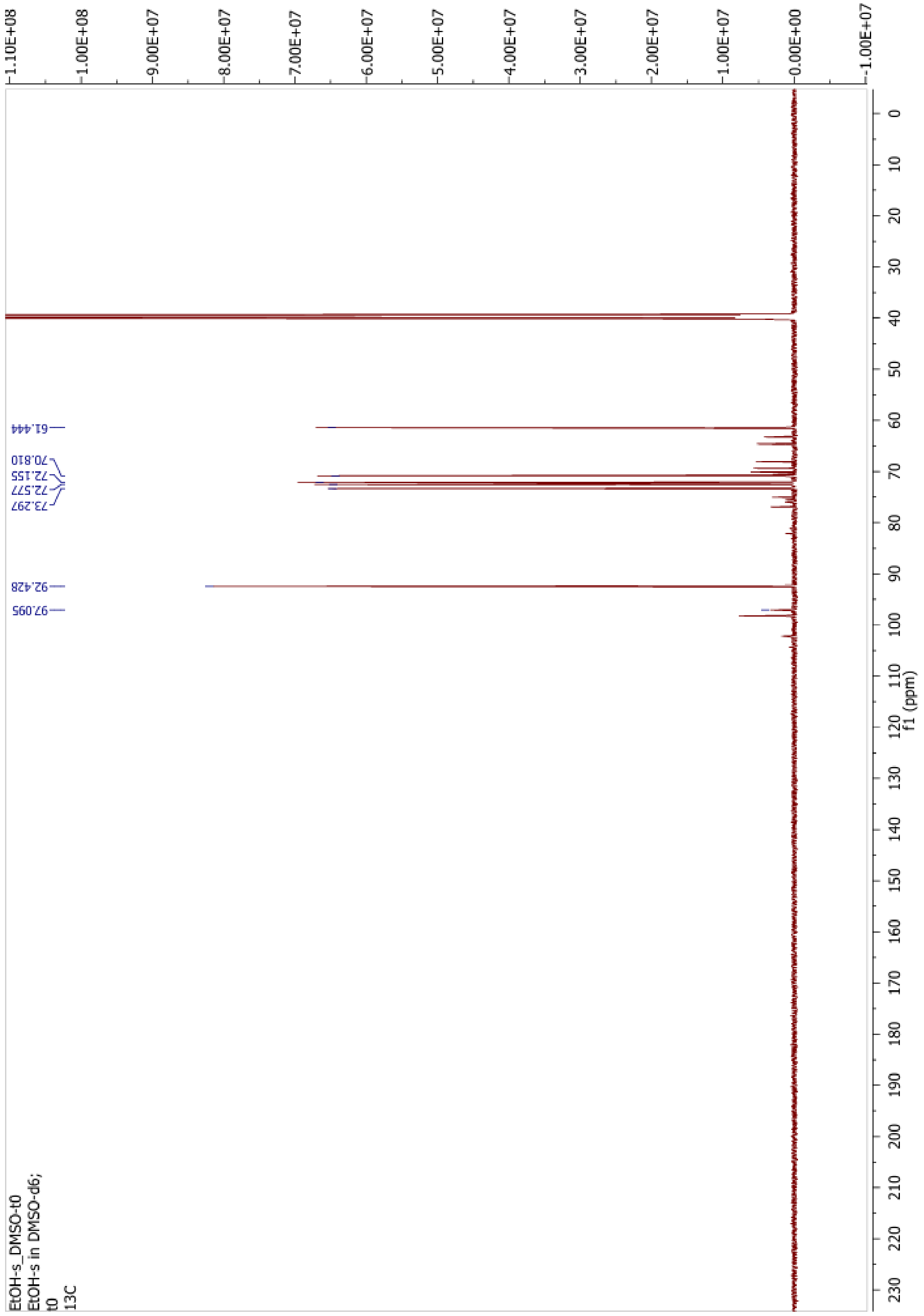

Figure S18. ^{13}C NMR Spectrum (DMSO-d_6) of the isolated α-D-glucopyranose-rich fraction at time zero (t_0).

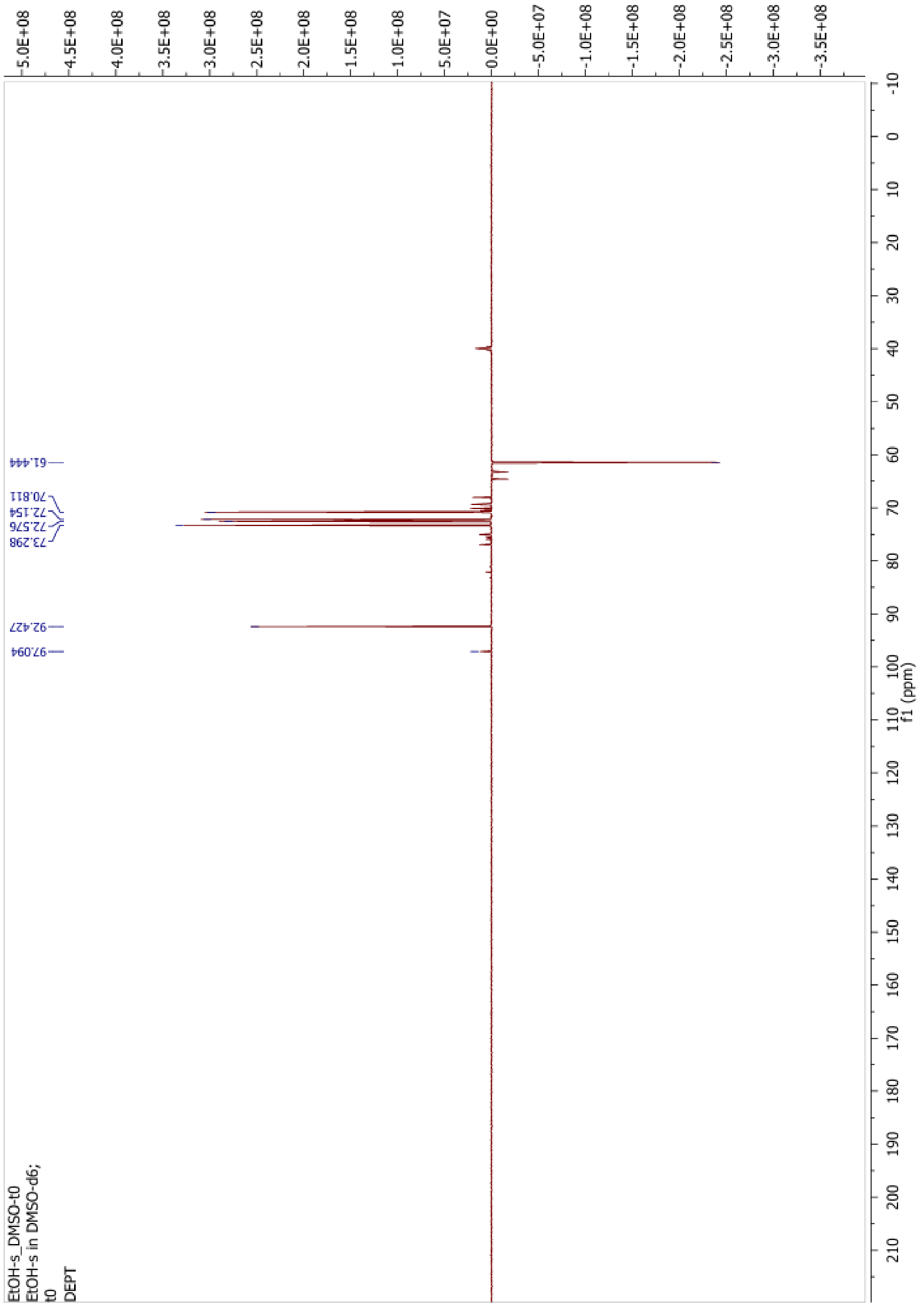

Figure S19. DEPT-135 Spectrum (DMSO-d_6) of the isolated α-D-glucopyranose-rich fraction at time zero (t_0).

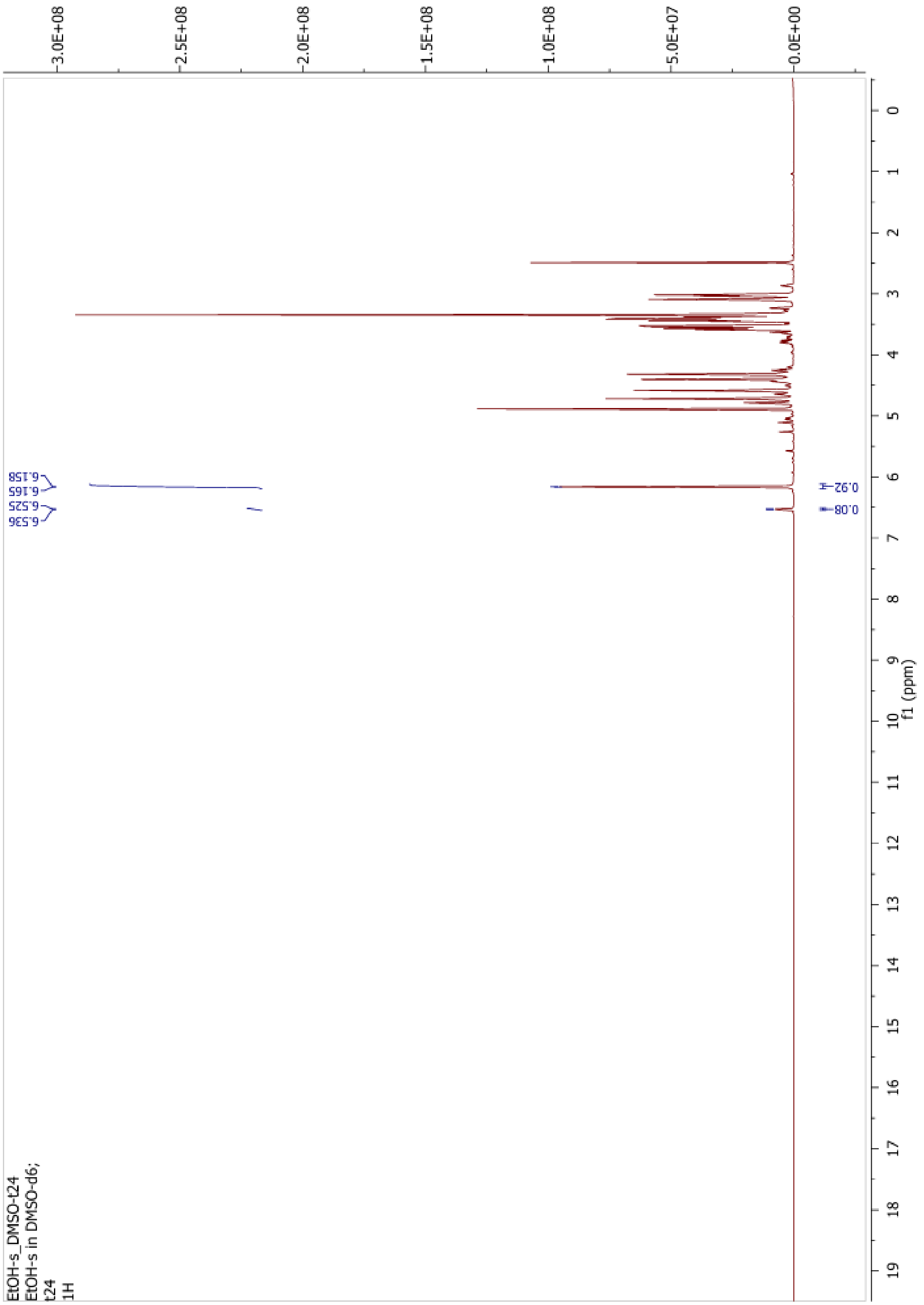

Figure S20. ^{1}H-NMR Spectrum (DMSO-d_6) of the isolated α-D-glucopyranose-rich fraction after 24 hours (t_{24}).

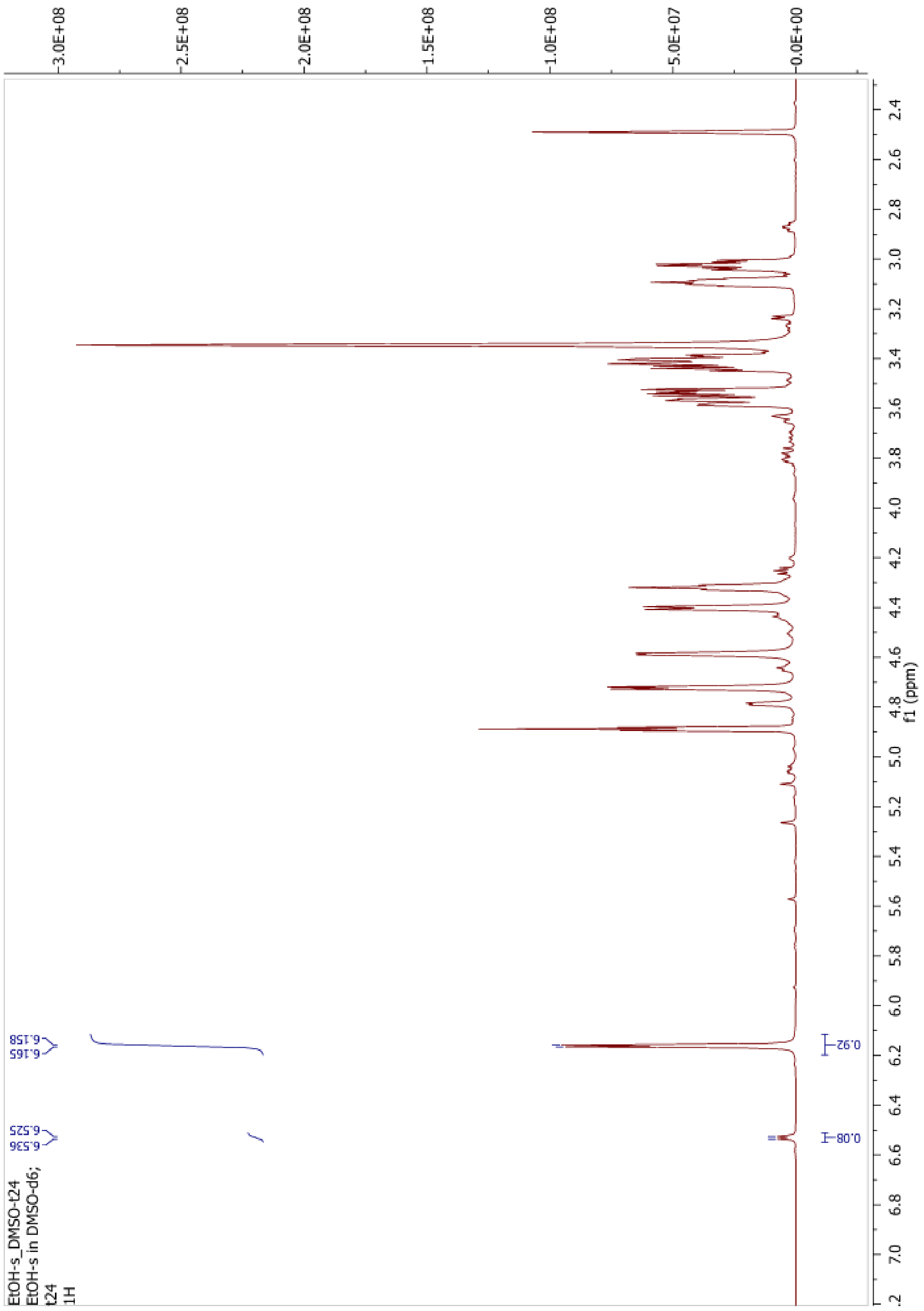

Figure S21. Expanded ^{1}H NMR Spectrum (DMSO-d_6) of the isolated α-D-glucopyranose-rich fraction after 24 hours (t_{24}).

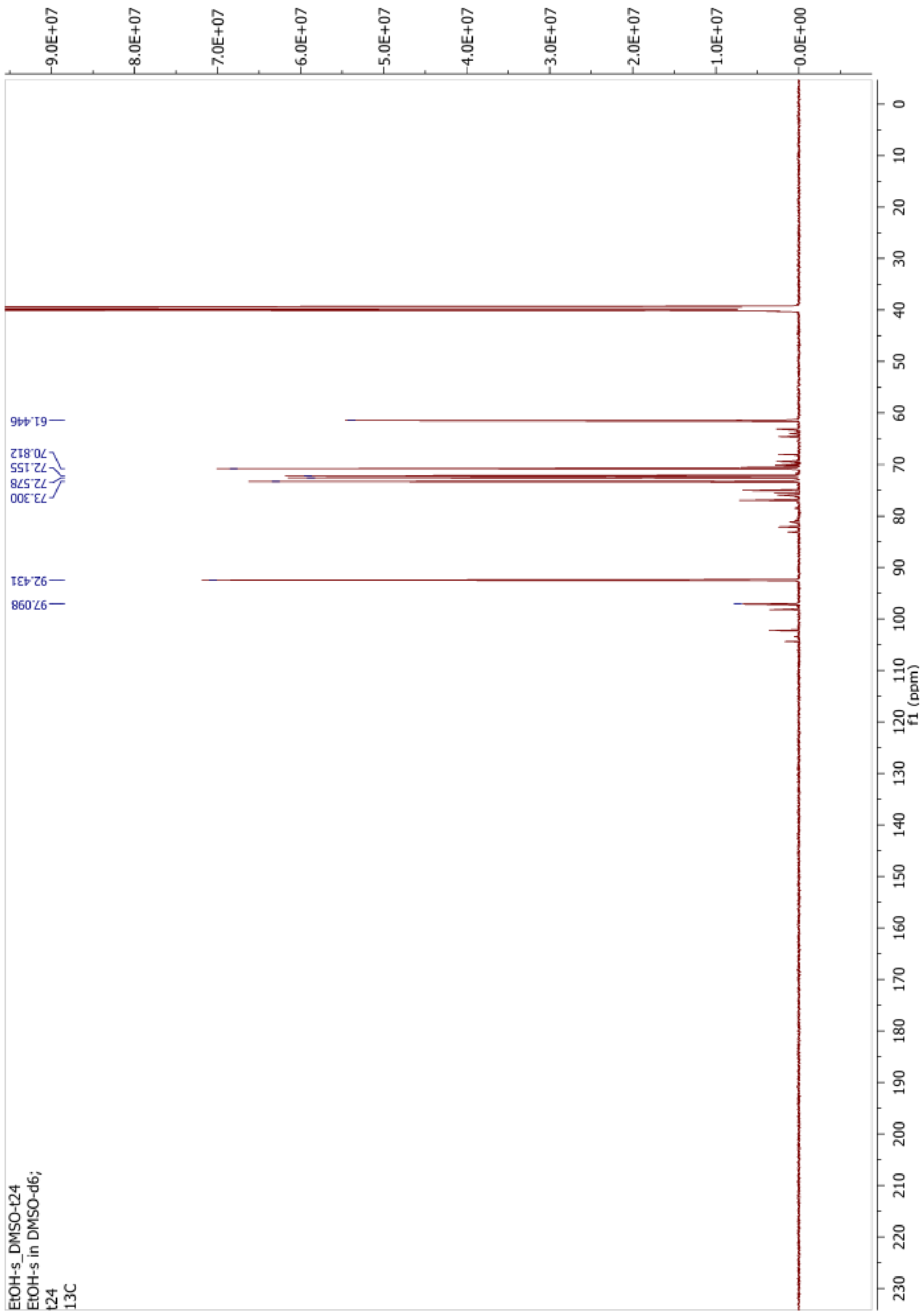

Figure S22. ^{13}C NMR Spectrum (DMSO-d_6) of the isolated α-D-glucopyranose-rich fraction after 24 hours (t_{24}).

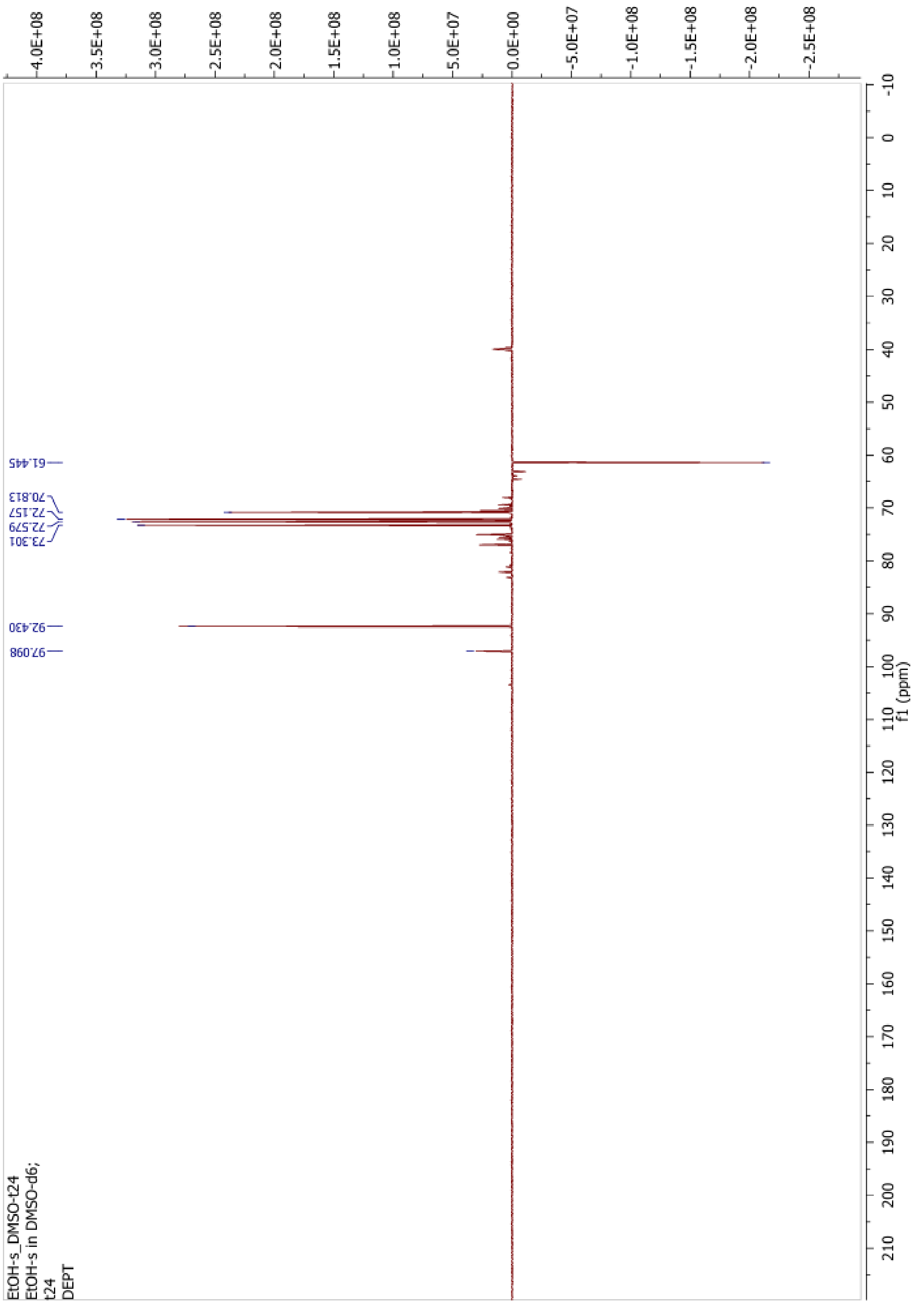

Figure S23. DEPT-135 Spectrum (DMSO-d_6) of the isolated α-D-glucopyranose-rich fraction after 24 hours (t_{24}).

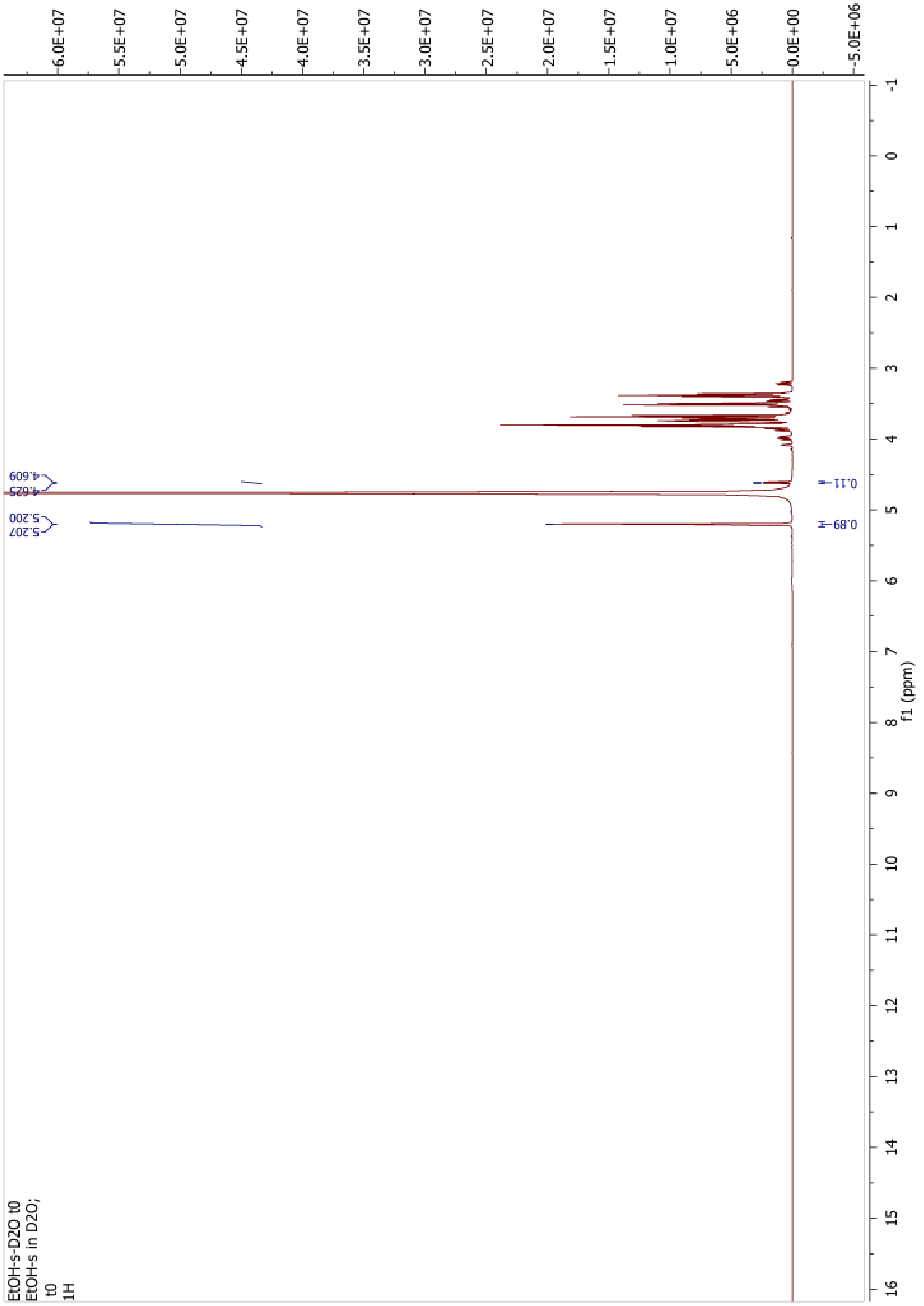

Figure S24. ^{1}H-NMR Spectrum (D_2O) of the isolated α-D-glucopyranose-rich fraction at time zero (t_0).

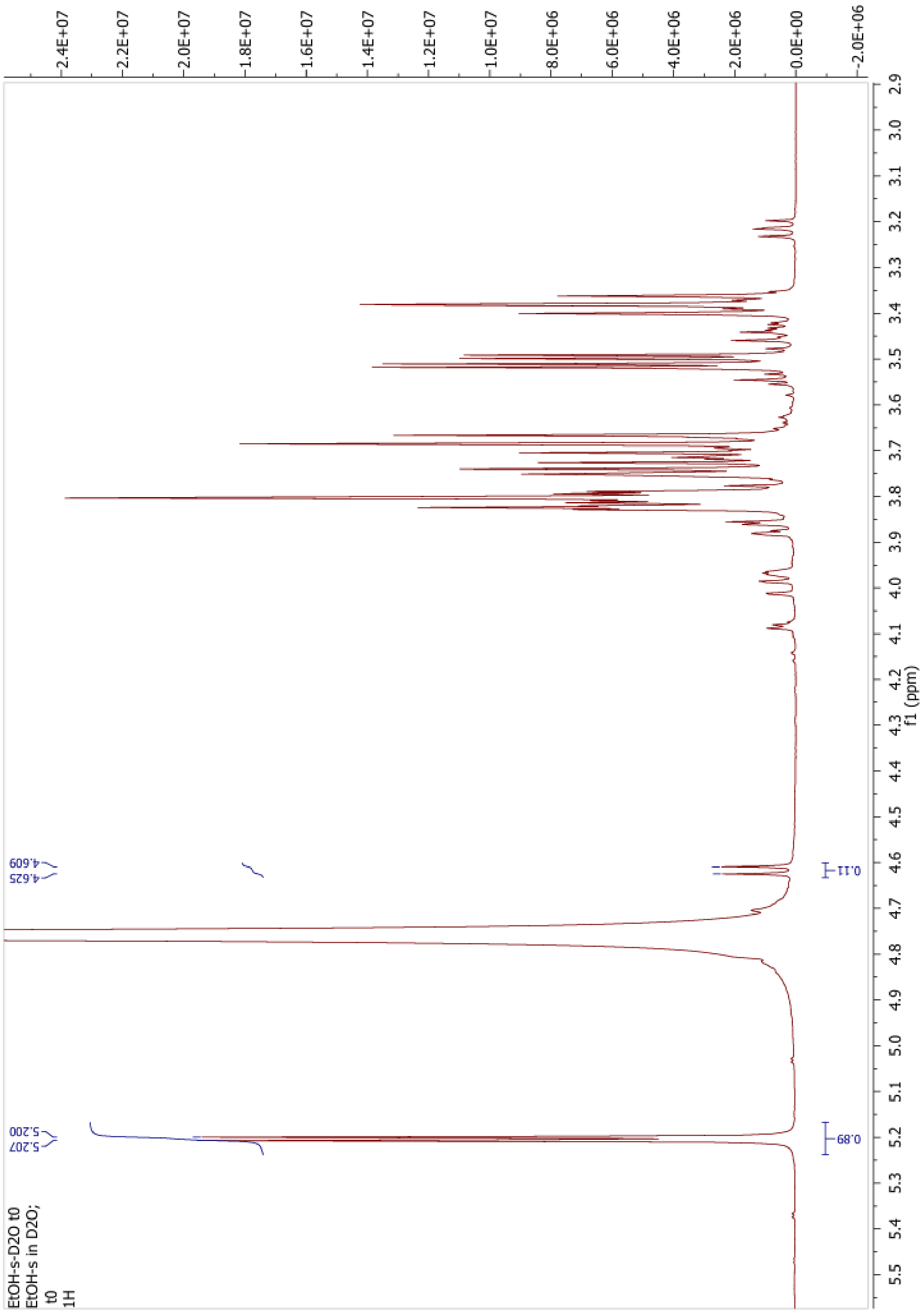

Figure S25. Expanded ^{1}H NMR Spectrum (D_2O) of the isolated α-D-glucopyranose-rich fraction at time zero (t_0).

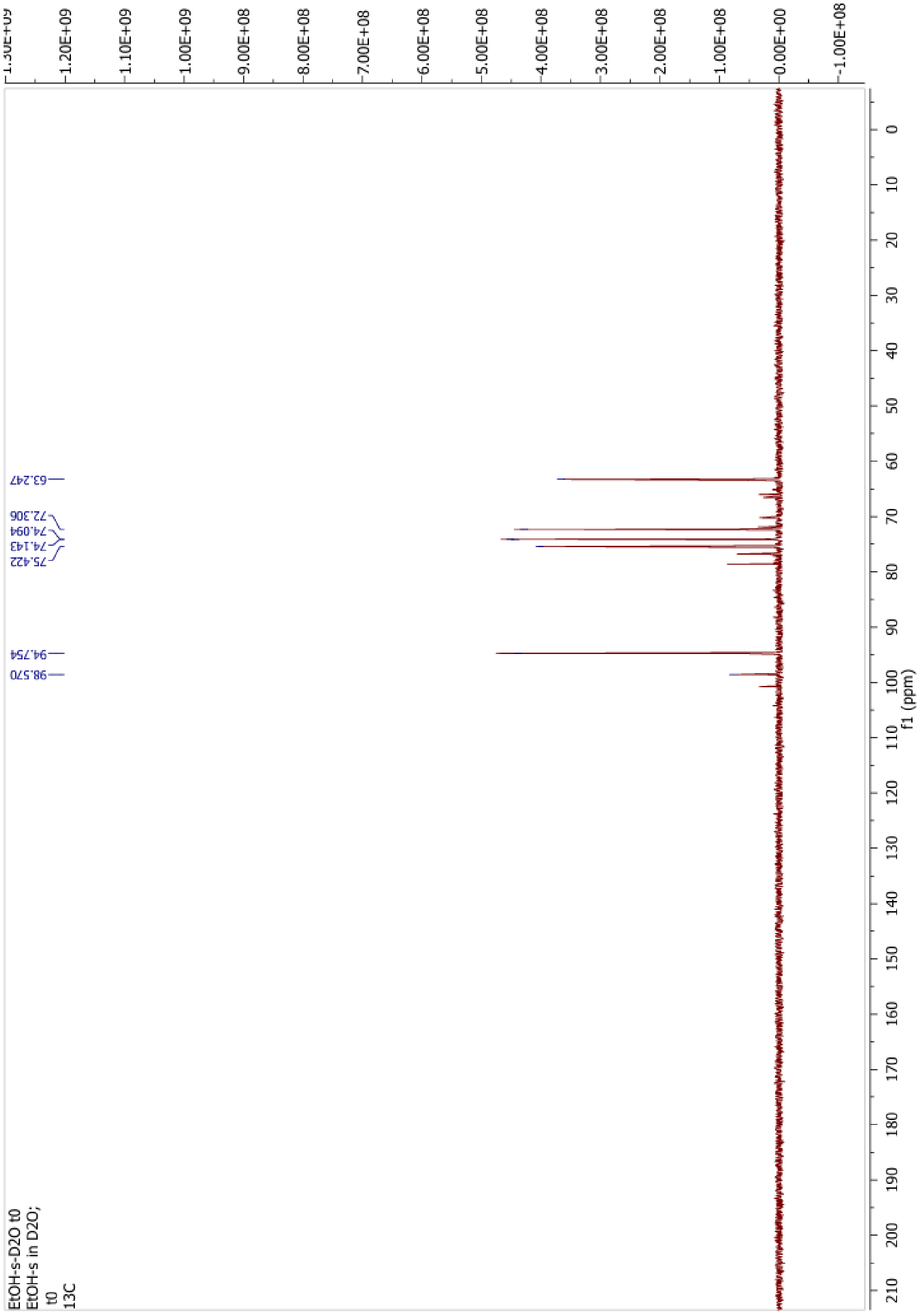

Figure S26. ^{13}C NMR Spectrum (D_2O) of the isolated α-D-glucopyranose-rich fraction at time zero (t_0).

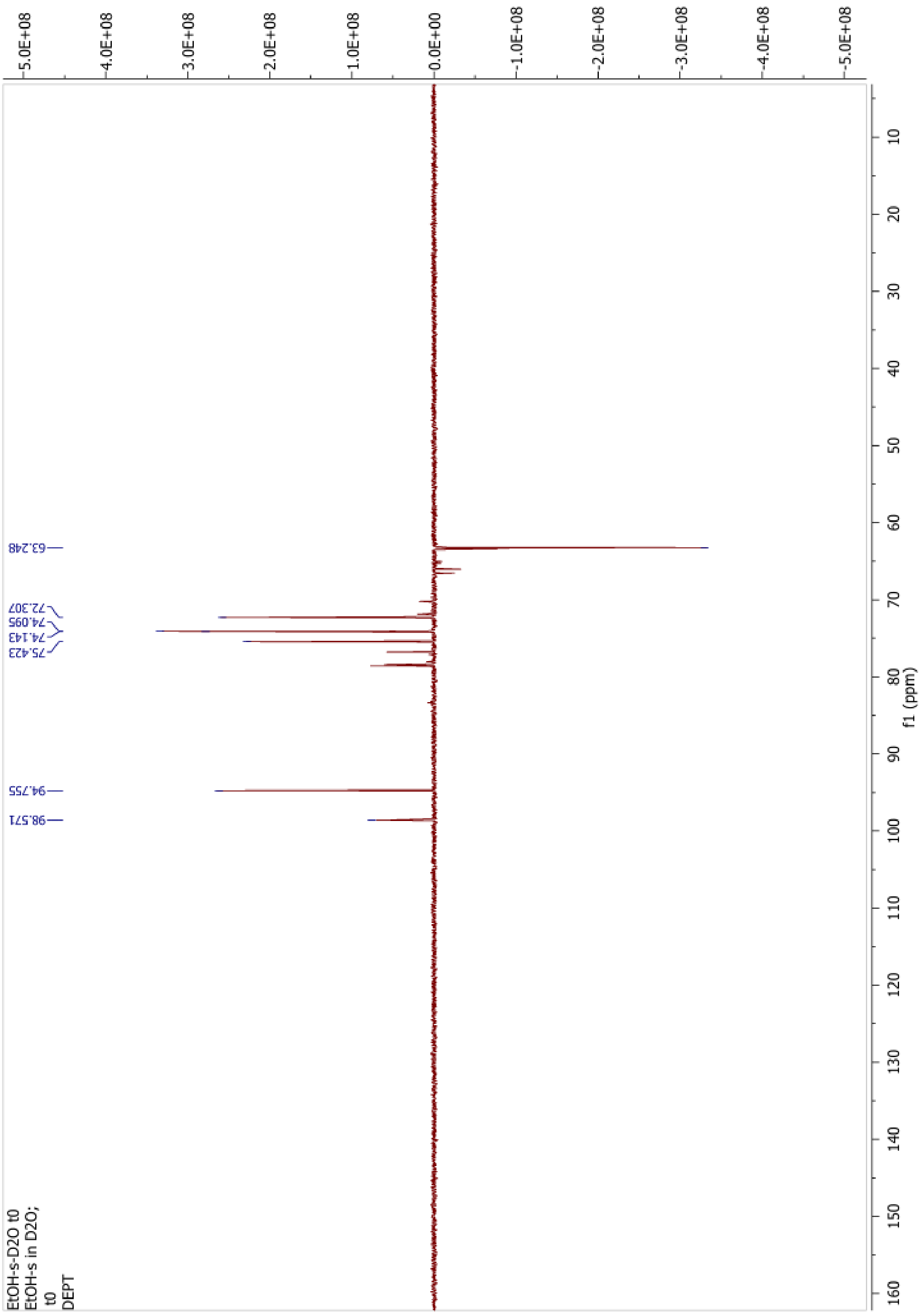

Figure S27. DEPT-135 Spectrum (D_2O) of the isolated α-D-glucopyranose-rich fraction at time zero (t_0).

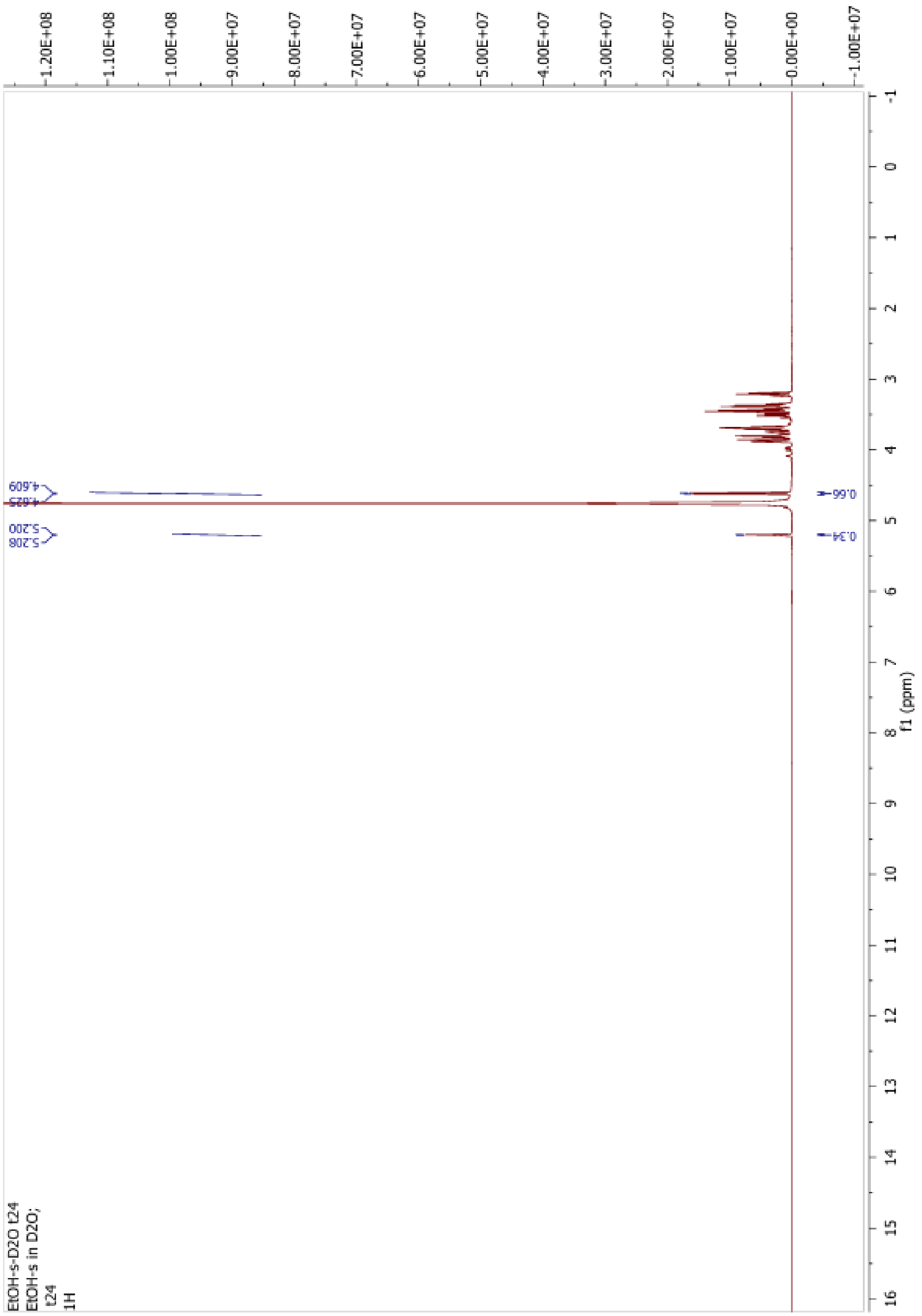

Figure S28. ^{1}H-NMR Spectrum (D_2O) of the isolated α-D-glucopyranose-rich fraction after 24 hours (t_{24}).

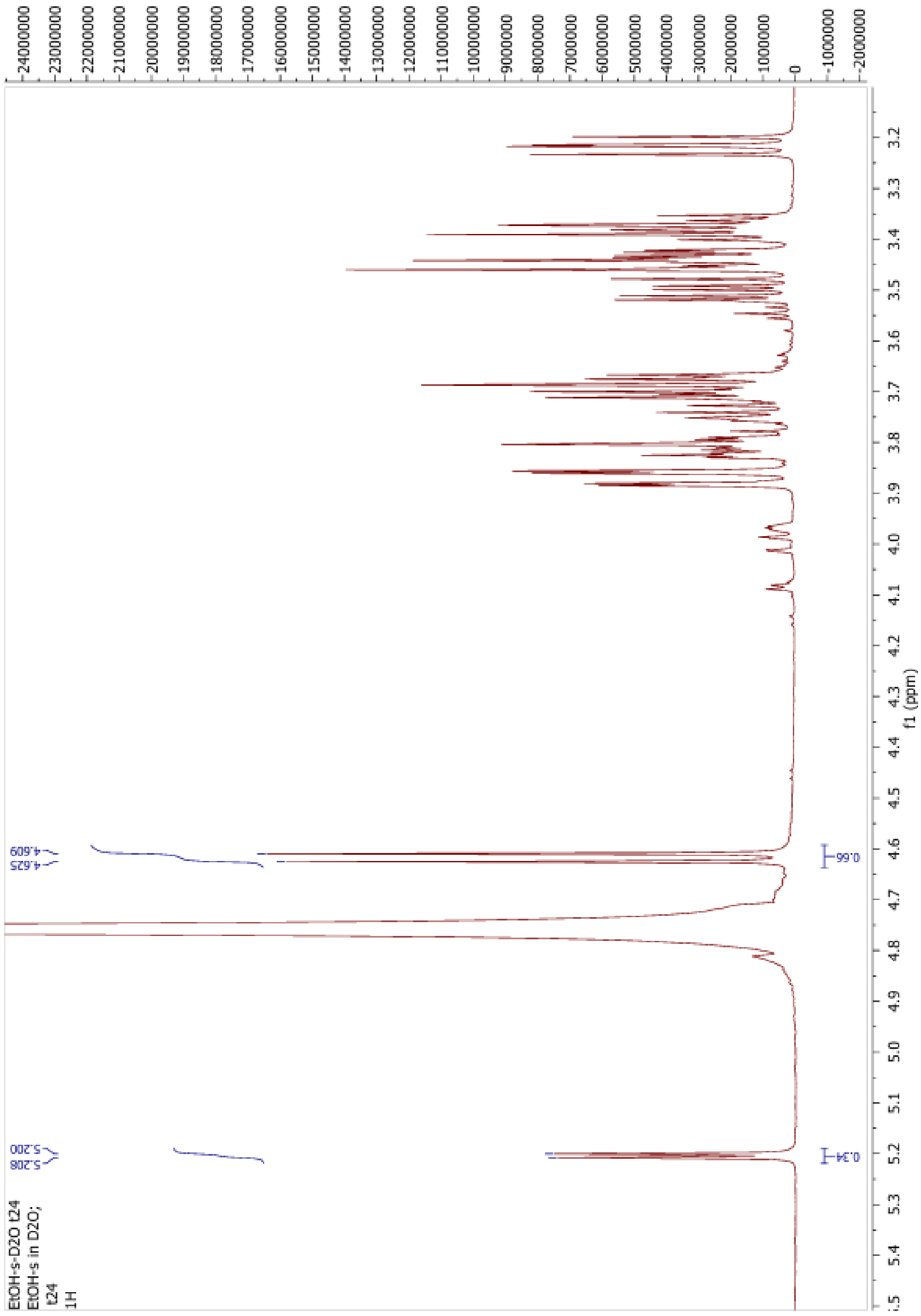

Figure S29. Expanded ^{1}H NMR Spectrum (D_2O) of the isolated α-D-glucopyranose-rich fraction after 24 hours (t_{24}).

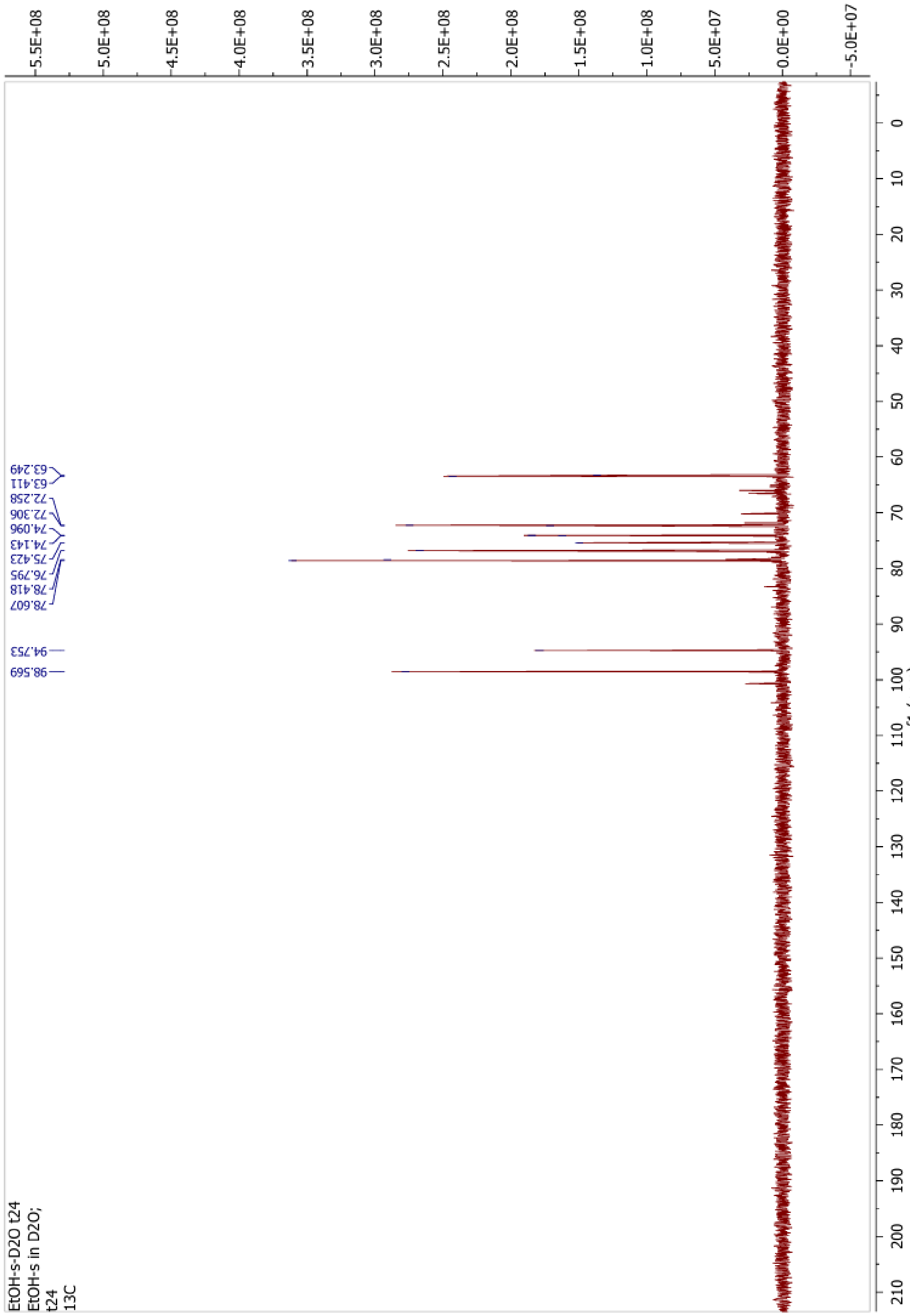

Figure S30. ^{13}C NMR Spectrum (D_2O) of the isolated α-D-glucopyranose-rich fraction after 24 hours (t_{24}).

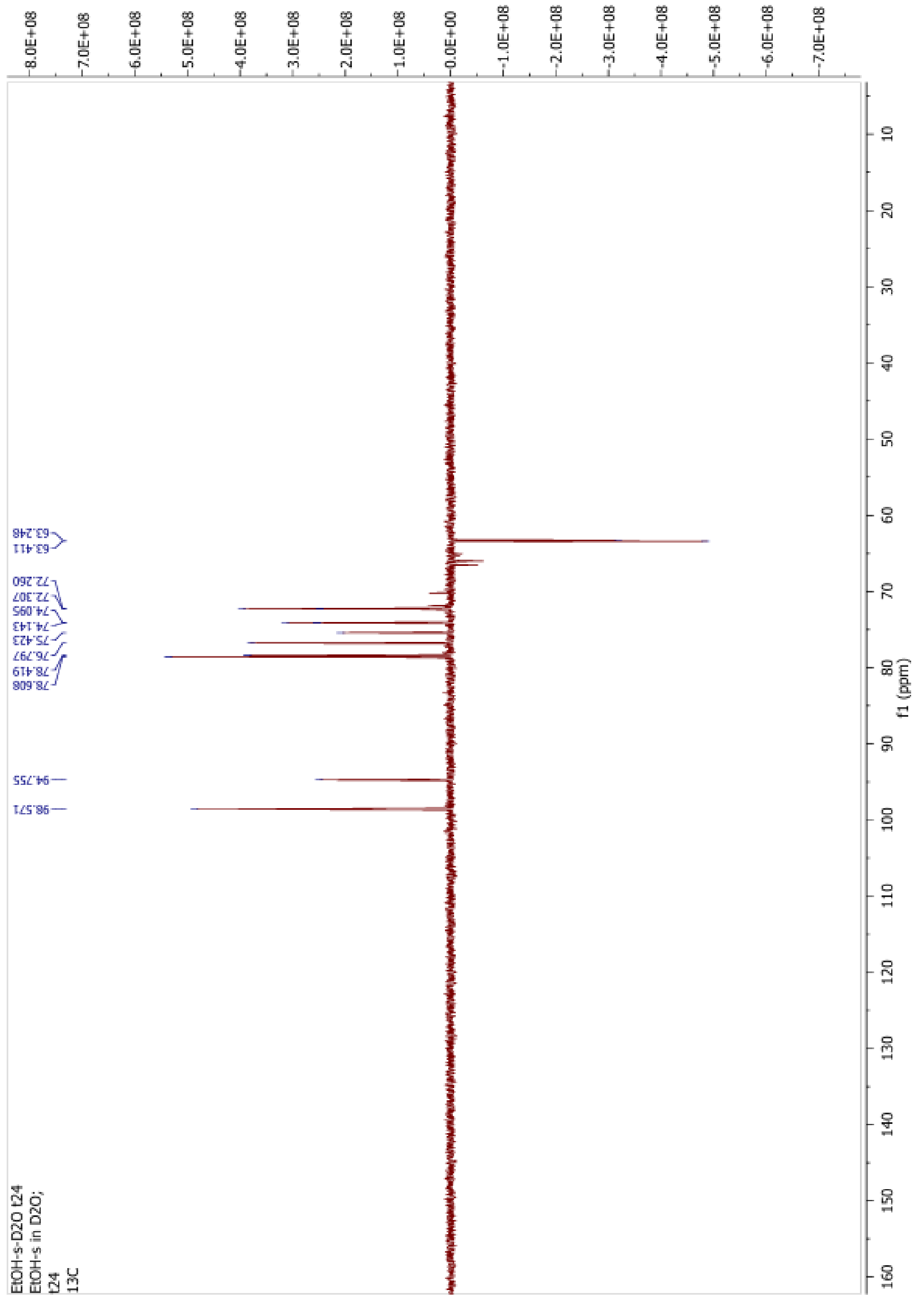

Figure S31. DEPT-135 Spectrum (D_2O) of the isolated α-D-glucopyranose-rich fraction after 24 hours (t_{24}).

Table S1. Optical rotation measurements of the isolated α-D-glucopyranose-rich fraction recorded over time during mutarotation in water at 20 °C.

**Time (min)**	**Observed rotation, α (°)**	**Specific rotation $[\alpha]_D^{20}$ (°)**
0	+0.923	+92.3
5	+0.882	+88.2
10	+0.848	+84.8
15	+0.815	+81.5
20	+0.785	+78.5
25	+0.758	+75.8
30	+0.733	+73.3
35	+0.710	+71.0
110 (1 h 50 min)	+0.518	+51.8
180 (3 h)	+0.465	+46.5
1080 (18 h, equilibrium)	+0.438	+43.8

Measurements were performed in water at 20 °C using a 1 dm polarimeter tube at 589 nm. Sample concentration was 1.0 g/100 mL (1% w/v).

<table>
<tr>
<td>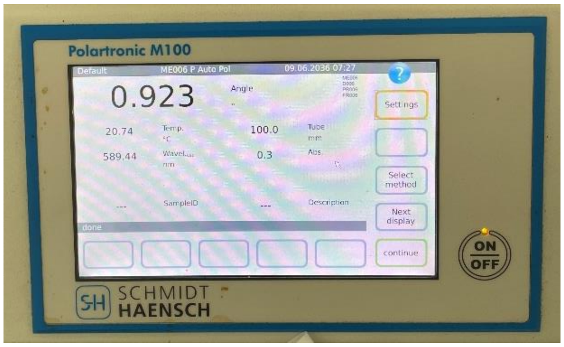
</td>
<td>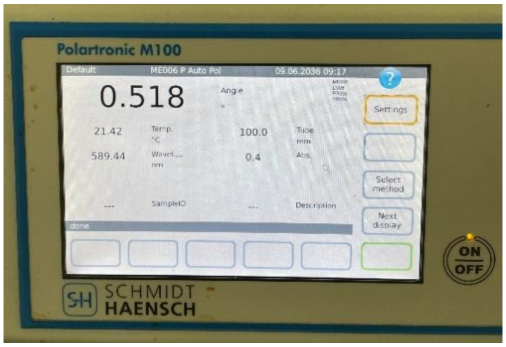
</td>
</tr>
<tr>
<td>Time: 0 h</td>
<td>Time: 1 h 50 min</td>
</tr>
<tr>
<td>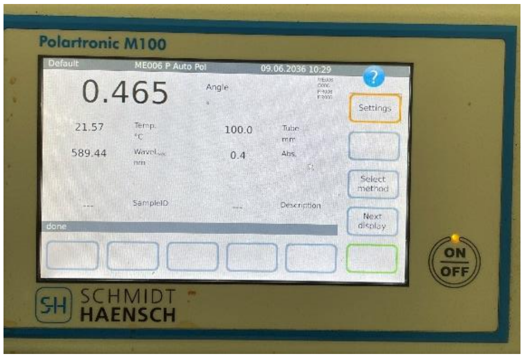
</td>
<td>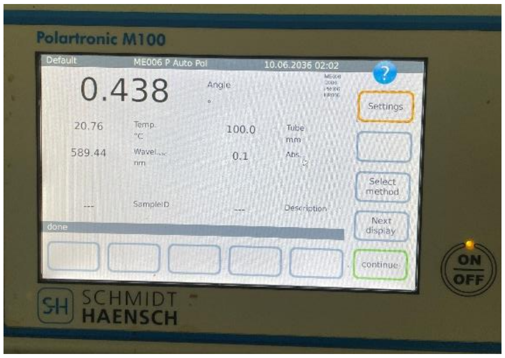
</td>
</tr>
<tr>
<td>Time: 3 h 2 min</td>
<td>Time: 18 h 35 min (equilibrium)</td>
</tr>
</table>

Figure S32. Representative polarimeter display images recorded immediately after dissolution (0 h), (1 h 50 min), (3 h 2 min) and after 18 h 35 min (equilibrium) to monitor changes in optical rotation during mutarotation under the experimental conditions described in the main manuscript.

Table S2. Reproducibility of the isolated α-D-glucopyranose-rich fraction across independent experimental batches.

**Date fruit sample batch**	**Analysis performed**
Batch 1	TLC, LC–MS, ^{1}H NMR, ^{13}C NMR, DEPT-135, specific rotation
Batch 2	TLC, LC–MS, ^{1}H NMR, ^{13}C NMR, DEPT-135
Batch 3	TLC, LC–MS, ^{1}H NMR, ^{13}C NMR, DEPT-135
