# Supplementary material for: Reproducible Ethanol-Induced Precipitation and Systematic Characterization of an α-D-Glucopyranose-Rich Solid from Khalas Date Fruit (Phoenix dactylifera L.)
Source: Foods. 2026 Oct 13;15(20):3632. doi: 10.3390/foods15203632 (PMC13703318; doi:10.3390/foods15203632)
Supplement: Supplementary file 1 [file foods-15-03632-s001.zip › Response to the Editor and Reviewers.pdf]

Response to the Editor and Reviewers

Manuscript ID: foods-4531481 (Resubmission)

Revised Title: *Reproducible Ethanol-Induced Precipitation and Systematic Characterization of an α-D-Glucopyranose-Rich Solid from Khalas Date Fruit (Phoenix dactylifera L.)*

Dear Editor and Reviewers,

We sincerely thank the Editor and the Reviewers for their careful evaluation of our manuscript and for their constructive comments and suggestions. We have carefully considered all comments and revised the manuscript accordingly. All revisions have been highlighted in the revised manuscript. Here is a point-by-point responses to all raised issues.

Response to the Academic Editor

Academic Editor's Comment. A new submission would only be considered if the manuscript is substantially reworked to address the major scientific, methodological, or structural concerns.

Response. We thank the Academic Editor for the opportunity to resubmit our manuscript for consideration in Food.

We have now extensively worked on the manuscript to meet the journal expectations as well as the reviewer's comments. The title and central framing were revised; the novelty and knowledge gap were clarified around the reproducible direct ethanol-induced isolation of an α-D-glucopyranose-rich fraction without prior aqueous extraction and its anomer-specific characterization; additional relevant literature was incorporated; the available characteristics and limitations of the original date material were explicitly stated; the LC–MS methodology and its analytical role were clarified; the low isolated yield was distinguished from total sugar recovery; the D-glucose/D-fructose ratio is now stated explicitly as an approximate 15.1:1 model-based estimate; TLC is presented only as preliminary qualitative support; and claims concerning crystallinity were removed because DSC and XRD were not performed. The Abstract, Introduction, Materials and Methods, Results and Discussion, and Conclusion were revised accordingly, and all changes are highlighted in the revised manuscript using the Track Changes available in the Microsoft Word program.

Reviewer 1 – Round 2

Comment. I believe the authors have adequately addressed the reviewers' comments, and the manuscript is acceptable in its current form.

Response. We sincerely thank the reviewer for the positive assessment of the revised manuscript and for the constructive comments provided during the review process. We greatly appreciate the reviewer's time and consideration.

Reviewer 2 – Round 2

No additional comments were provided in Round 2.

Reviewer 3 – Round 2

Dear Authors,

There are several flaws that need to be addressed by this manuscript and were not attended to in the first round.

Response. We thank the reviewer for the additional comments. The manuscript has been substantially revised to address the remaining concerns and to clarify the scope, novelty, analytical limitations, and interpretation of the results.

Comment 1. What is the novelty of the work?

Response. We thank the reviewer for this important comment. The novelty of the present study lies in the reproducible direct ethanol-induced isolation of an α-D-glucopyranose-rich fraction from Khalas date fruit without prior aqueous extraction, followed by anomer-specific NMR characterization of the isolated material and observation of its subsequent mutarotation in aqueous solution. Previous NMR studies of date sugars either involved aqueous extraction or reconstitution in D_2O-containing media. The Introduction, Abstract, and Conclusion have therefore been revised to clarify this distinction and the specific novelty of the present work.

Comment 2. What is the ratio glucose /fructose?

Response. The estimated composition was 93.8% D-glucose and 6.2% D-fructose, corresponding to an approximate D-glucose/D-fructose ratio of 15.1:1 under the applied binary model. This ratio is now stated explicitly in Section 3.6. As clarified in the manuscript, these values are model-based estimates rather than an independent absolute compositional determination.

Comment 3. Why the low yield obtained (lower than 1%), it is not natural for this raw material. Even if it would be for characterization properties, this could produce confusion in the reader.

Response. The reported yield of 0.2009 ± 0.0062% represents the selectively precipitated fraction relative to the initial date-fruit mass and not the total sugar content or glucose recovery. The objective of the study was to isolate and characterize the precipitated fraction and its anomeric composition rather than to maximize sugar recovery. This distinction has now been clarified in Section 3.2 and in the Conclusion.

Comment 4. Characterization of the original date properties.

Response. Additional information on the original date material has been provided in Section 2.2. The samples were Khalas dates at the Tamer (dried) stage, obtained in Muscat, Oman, from three independent batches collected in September 2016, September 2017, and September 2021. Moisture content, °Brix, and the initial quantitative glucose and fructose contents were not determined at the time of collection, and this is now explicitly stated in the manuscript.

Comment 5. There is a mixture of accurate characterization techniques (NMR, LC-MS) and old ones /TLC) without conclusive results. The authors are treating a mixture of two well-known sugars as if it were an identification of novel metabolites.

Response. We agree that TLC does not provide conclusive identification or compositional determination. It is now explicitly presented only as a preliminary qualitative comparison and is not used to determine purity or composition. LC–MS is used only to confirm the presence of hexose species, while identification of D-glucose as the predominant component and support for the minor D-fructose component are based primarily on NMR spectroscopy; their relative proportions are estimated by polarimetry. We also clarify that the study does not claim glucose or fructose as novel metabolites in date fruit. The novelty concerns the direct isolation procedure and the anomeric characterization of the isolated fraction before subsequent aqueous mutarotation.

Comment 6. The DSC analysis was not performed.

Response. We agree that DSC would be required for detailed thermal or solid-state characterization. DSC was not performed in this study, and the revised manuscript makes no claim based on DSC or regarding a defined crystalline phase. The melting point is now presented only as a supporting physical characteristic and not as evidence of solid-state phase identity.

Comment 7. The authors mention crystallization, but XRD was not made to determine the purity of the samples nor the crystalline degree.

Response. XRD was not performed as the material was precipitated as normal solid, not crystalline. This typos has been corrected throughout the manuscript. The title now refers to an α-D-glucopyranose-rich solid, and neither XRD nor the melting point is used to establish sample purity or crystalline degree. Further solid-state analysis is acknowledged as future work.

We would like again to thank the Editor and all Reviewers for their valuable comments and constructive suggestions. The revised resubmitted version is now substantially improved and we hope it is now more suitable for the *Foods*.

Sincerely yours,

Younis Baqi, PhD
